# Analysis of Aldo–Keto Reductase Gene Family and Their Responses to Salt, Drought, and Abscisic Acid Stresses in *Medicago truncatula*

**DOI:** 10.3390/ijms21030754

**Published:** 2020-01-23

**Authors:** Jie Yu, Hao Sun, Jiaju Zhang, Yiyao Hou, Tiejun Zhang, Junmei Kang, Zhen Wang, Qingchuan Yang, Ruicai Long

**Affiliations:** Institute of Animal Sciences, Chinese Academy of Agricultural Sciences, Beijing 100193, China; yujie090412@163.com (J.Y.); sunhao921023@163.com (H.S.); zhangjjzeus@163.com (J.Z.); tiejunzhang@126.com (T.Z.); kangjunmei@caas.cn (J.K.); wangzhen@hotmail.com (Z.W.)

**Keywords:** aldo–keto reductases, *Medicago truncatula*, expression analysis, NaCl, PEG6000, abscisic acid

## Abstract

Salt and drought stresses are two primary abiotic stresses that inhibit growth and reduce the activity of photosynthetic apparatus in plants. Abscisic acid (ABA) plays a key role in abiotic stress regulation in plants. Some aldo–keto reductases (AKRs) can enhance various abiotic stresses resistance by scavenging cytotoxic aldehydes in some plants. However, there are few comprehensive reports of plant AKR genes and their expression patterns in response to abiotic stresses. In this study, we identified 30 putative AKR genes from *Medicago truncatula*. The gene characteristics, coding protein motifs, and expression patterns of these *MtAKR*s were analyzed to explore and identify candidate genes in regulation of salt, drought, and ABA stresses. The phylogenetic analysis result indicated that the 52 AKRs in *Medicago truncatula* and *Arabidopsis thaliana* can be divided into three groups and six subgroups. Fifteen *AKR* genes in *M. truncatula* were randomly selected from each group or subgroup, to investigate their response to salt (200 mM of NaCl), drought (50 g·L^−1^ of PEG 6000), and ABA (100 µM) stresses in both leaves and roots. The results suggest that *MtAKR*1, *MtAKR*5, *MtAKR*11, *MtAKR*14, *MtAKR*20, and *MtAKR*29 may play important roles in response to these stresses.

## 1. Introduction

Biotic and abiotic stresses can disrupt the balance between endogenous levels of reactive oxygen species and antioxidant-induced injury to plants [1,2]. Drought and soil salinity are the most destructive abiotic stresses affecting agriculture [3]. Soil salinity is one of the main factors limiting crop yield in arid and semiarid regions [4,5]. Climate changes might be leading to more severe abiotic stress [6]. The effect of abscisic acid (ABA) to mediate drought and salt stress has been extensively studied. ABA facilitates plant regulation by inducing the expression of genes encoding proteins and enzymes involved in cell dehydration tolerance [7]. Plants have to respond to periodic and unpredictable environmental stresses during growth and development. Plants try to detoxify reactive oxygen species—reactive aldehydes induced by stress to less toxic alcohols or carboxylic acids by upregulating expression a few of enzymes with oxido-reductase activity. There are three protein super families capable of oxidoreduction of a wide range of aldehyde and keto substrates were convened in these reactions: Short-chain dehydrogenases/reductases (SDRs), long-chain alcohol/aldehyde dehydrogenases (ADHs), and aldo–keto reductases (AKRs) [8,9]. The AKRs, part of the oxido-reductase super family, are stress-regulated genes and play an important role in the cellular response to electrophilic, osmotic, and oxidative stress, depending on the presence of coenzyme NAD (P)(H) (Nicotinamide Adenine Dinucleotide Phosphate) [10]. AKRs are present in prokaryotes and eukaryotes, with 18 protein families in identified phyla [11]. Several AKRs have been reported to effectively detoxify cytotoxic aldehydes from abiotic-stress-related glycolysis, to improve plant stress resistance. For example, aldo–keto reductase can eliminate reactive carbonyls in *Synechocystis* [12]; cadmium tolerance was increased in tobacco transformed with *IbAKR*, which was cloned from sweet potato (*Ipomoea batatas*) [11]; a novel aldo–keto reductase gene, *PpAKR1*, from peach (*Prunus persica*) confers higher tolerance to salt in *Arabidopsis* [13]; and transgenic tobacco expressing *ALDRXV4* from *Xerophyta viscosa* has increased resistance to drought and salt [14]. However, systematic studies of AKR family with stress resistance in a single species have not been reported.

As the foundation for dairy and meat production, leguminous forage is used with industrial raw materials and is the second most used forage product in the world, after the human food Gramineae [15,16]. *Medicago truncatula* is an annual herb and leguminous model plant used for forage due to its compact genome size (~500 Mb) [17,18], high seed-setting rate, high genetic transformation rate, and extensive collection of mutants [19]. *M. truncatula* also exhibits a wide cultivation adaptability to varying environmental conditions, including barren soil and saline soils [20]. With the characteristics mentioned above, *M. truncatula* has been widely used as a model plant of leguminous forage in studies of salt tolerance [21], nitrogen fixation [22], drought, and re-watering [23]. Recent developments in comparative genomics methods have facilitated the utilization of genomic discoveries in this model plant for study of other legumes, including most members of the agriculturally important Papilionoideae subfamily [24,25,26]. Bioinformatics studies of the sequenced *M. truncatula* suggest that this genome can serve as a reference genome for closely related Papilionoid species and may enable studies of orthologous genes and gene families both within *Medicago* and among more different plants [20,27].

In this study, the comprehensive identification and characterization of all putative *AKR* genes in *M. truncatula* were performed. The general characteristics of the identified *AKRs*, including their gene structure, common protein features, and phylogenetic relationship, were determined. The expression profiles of *AKR* genes in leaves and roots subjected to control conditions, salt, dehydration, and ABA treatments were also investigated, to provide insight into the potential roles of AKRs in the abiotic-stress regulation of plants. Studies have shown that resistant breeding of genes with significant changes in transcription levels under stress may be of great significance [28,29,30]. The main purpose of this study is to explore and identify candidate genes in regulation of salt, drought, and ABA stresses and provide the basis for the further elucidation of the biological function of *MtAKRs* and their regulation in abiotic stresses.

## 2. Results

### 2.1. Identification of AKR Genes in Medicago Truncatula

The members of the *M. truncatula* AKR family were identified by using the Pfam database (http://www.pfam.xfam.org/ European Bioinformatics Institute, Hinxton, Cambridgeshire, UK.) by searching for the AKR domain (PF00248). After removal of sequences with similarity less than 40% [20], sequences lacking an NADP-dependent oxidoreductase domain (cd06660), and functional redundancy, a total of 30 genes were obtained. The gDNA length, CDS length, protein ID, protein length, protein mass (kDa), *pI* (isoelectric point), GRAVY (the grand average of hydropathy value), and predicted subcellular localization of these genes are listed in Table 1, and the sequences of CDS and gDNA are listed in Supplementary Material S1. The gDNAs and CDSs of the *MtAKR*s range from 1220 to 5971 bp and 765 to 1536 bp, respectively, and the genes encode polypeptides 254 to 511 amino acids in length. The mass (molecular weight) of these proteins ranges from 28.053 to 56.964 kDa, with *PI* values that range from 5.25 to 9.2 (Table 1). The negative grand average of hydropathy values (GRAVY) indicates these MtAKRs are hydrophilic, with values between −0.462 and −0.096 (Table 1). The subcellular localization analysis predicted localization of four MtAKRs in chloroplasts, three MtAKRs in the mitochondrion, and one MtAKR in the secretory pathway (Table 1). The strongest predictions were for localization of MtAKR1 and MtAKR6 to chloroplasts and MtAKR22 and MtAKR30 to the secretory pathway and mitochondrion, respectively.

### 2.2. The Distribution of MtAKR Genes on the Chromosome

**Figure 1:**
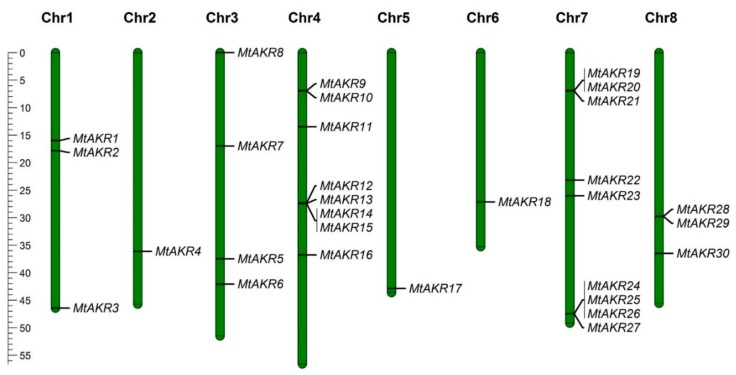
The chromosomal locations of the *MtAKR* genes.

The chromosomal location and length information for each *MtAKR* from Ensembl Plant (MedtrA17_4.0) (http://plants.ensembl.org) were visualized by using Mapchart (v 2.32) (Figure 1). The *MtAKR*s are distributed on each of the eight chromosomes. Five groups of genes (*MtAKR*9/*MtAKR*10, *MtAKR*12/*MtAKR*13/*MtAKR*14/*MtAKR*15, *MtAKR*19/*MtAKR*20/*MtAKR*21, *MtAKR*24/*MtAKR*25/*MtAKR*26/*MtAKR*27, and *MtAKR*28/*MtAKR*29) on the chromosome are tightly linked on chromosome 4, chromosomes 7, and chromosomes 8, respectively. Chromosomes 2 and 6 each contain one *MtAKR* gene. Three, four, and three *MtAKR* genes are respectively distributed on chromosomes 1, 3, and 8.

### 2.3. Structural Analysis of the MtAKR Genes

**Figure 2:**
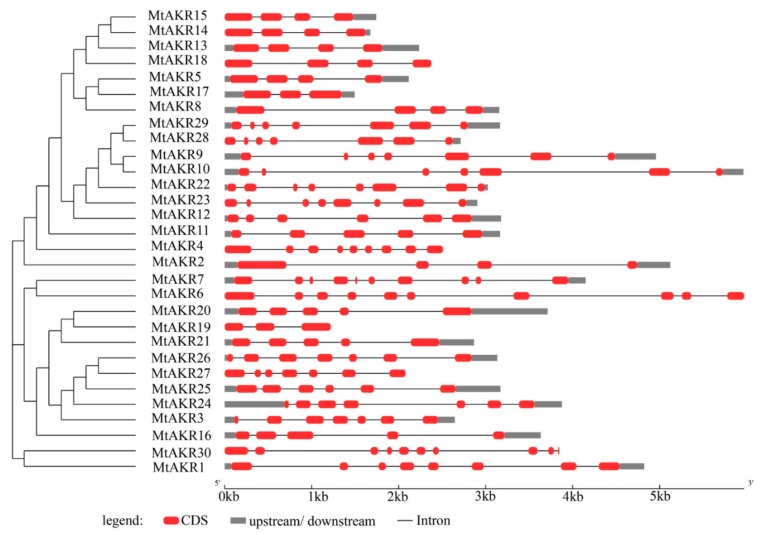
Structural and phylogenetic analyses of 30 identified *MtAKR* members. An unrooted tree of the *MtAKR*s constructed by neighbor-joining method, using MEGA 7. The gene organization of *MtAKR*s was explored by using the online tool GSDS 2.0 (http://gsds.cbi.pku.edu.cn/ Center for Bioinformatics, Peking University, Beijing, China).

The sequences of the thirty MtAKRs were aligned, and a phylogenetic tree was created by using the neighbor-joining method in MEGA 7, to characterize their relationships, based on the full-length protein sequences (Figure 2). The phylogenetic tree showed that these 30 MtAKRs could be divided into three classes. The first one contained the most proteins (MtAKR15, MtAKR14, MtAKR13, MtAKR18, MtAKR5, MtAKR17, MtAKR8, MtAKR29, MtAKR28, MtAKR9, MtAKR10, MtAKR22, MtAKR23, MtAKR12, MtAKR11, MtAKR4, and MtAKR2). The second one contained eleven MtAKRs (MtAKR7, MtAKR6, MtAKR20, MtAKR19, MtAKR21, MtAKR26, MtAKR27, MtAKR25, MtAKR24, MtAKR3, and MtAKR16). The third one was consisted of only two MtAKRs (MtAKR30 and MtAKR1). All the *MtAKR* genes contain introns, with exon numbers ranging from 3 to 10 (Figure 2). *MtAKR*17 and *MtAKR*19 each contain three exons. *MtAKR*2, *MtAKR*5, *MtAKR*8, *MtAKR*13, *MtAKR*14, *MtAKR*15, and *MtAKR*18 each contain four exons. *MtAKR*11, *MtAKR*16, *MtAKR*20, and *MtAKR*21 contain five exons. *MtAKR*12 and *MtAKR*25 each contain six exons. *MtAKR3*, *MtAKR*9, *MtAKR*10, *MtAKR*24, *MtAKR*26, *MtAKR*27, *MtAKR*28, and *MtAKR*29 each contain seven exons. *MtAKR*1, *MtAKR*22, and *MtAKR*23 contain eight exons. *MtAKR4* contains nine exons. *MtAKR*6, *MtAKR*7, and *MtAKR*13 each contain ten exons. The close family members typically had the same number of exons and genetic structures.

### 2.4. Conserved Motif in MtAKR Proteins

**Figure 3:**
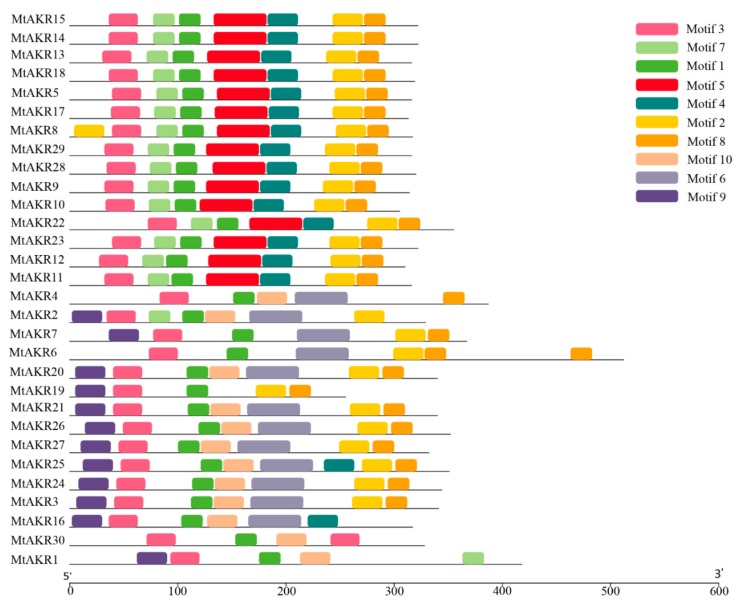
Distribution of the conserved motifs in different *MTAKR*s and the logos for each motif.

A total of 10 motifs ranging from 21 to 50 amino acids were identified by MEME analysis, as shown in Table 2. The obtained 10 motif sequences were submitted to Pfam search (http://pfam.xfam.org/search) for functional identification. Motif 1, motif 3, motif 5, motif 6, and motif 10 all describe aldo/keto reductase domain. Motif 1 and 3 are both present in all MtAKR proteins (Figure 3). The other motifs were designated as unknown motifs. Motif 2 is rich in Q (Gln) and was present in all MtAKR proteins except for MtAKR 1, MtAKR 4, MtAKR 16, and MtAKR 30. Similarly, motif 4 and motif 8 are both rich in Q (Gln), and motif 8 was present in all MtAKR proteins except for MtAKR1, MtAKR2, MtAKR16, and MtAKR30. Motif 7 is rich in D (Asp), and Motif 9 is rich in M (Met).

The motif information was input into the online software WebLogo (http://weblogo.berkeley.edu/ Department of Plant and Microbial Biology, University of California, Berkeley, US), and the motif logo was redrawn (Figure S1). In general, a sequence logo provides a richer and more precise description of a binding site than a consensus sequence [31,32]. For example, comparison of the logo to the consensus sequence shows a difference in the fourth amino acid in the first motif, with S or T at this site (presented here as S).

### 2.5. Cis-Acting Regulatory Elements in the Promoter of MtAKR Genes

**Figure 4:**
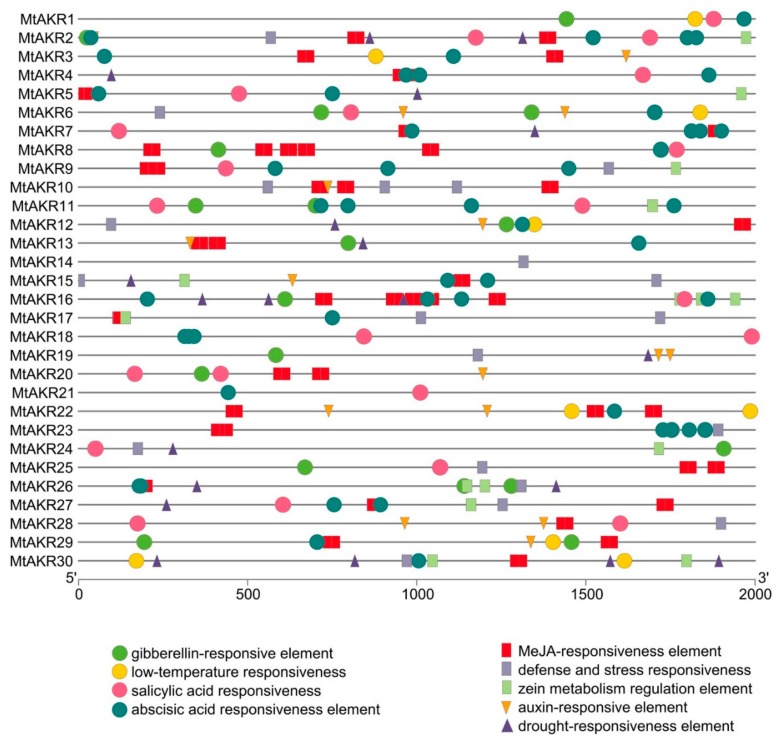
Distribution of major stress- and hormone-related cis-acting regulatory elements in the promoters of putative *M. truncatula AKR* genes. TC-rich repeats (defense and stress), MBS (drought), and LTR (low-temperature responsive) elements, TGA- element/AuxRR-core (auxin), O2-site (zein metabolism), TCA-element (salicylic acid), ABRE (abscisic acid), GARE-motif /P-box /TATC-box (gibberellin), and CGTCA-motif /TGACG-motif (MeJA responsive element) are represented by different symbols, as indicated in figure key at the bottom.

Cis-acting elements in promoter is important to determine the possible regulation mechanisms of *M. truncatula AKR* genes in the hormone and abiotic stress responses. The promoter sequences (the 2 kb of sequence upstream of the initiation codon ATG) were analyzed to identify potential cis-regulatory elements, using the PlantCARE database (http://bioinformatics.psb.ugent.be/webtools/plantcare/html Laboratoire Associé de l’Institut National de la Recherche Agronomique (France), Universiteit Gent, Gent, Belgium). Nine types of hormone- and stress-related cis-acting regulatory elements were identified in the promoters of *AKR* genes in *M. truncatula* (Figure 4). Three stress-related cis-acting elements were identified, including TC-rich repeats (defense and stress), MBS (drought), and LTR (low-temperature responsive) elements. Six hormone-related cis-acting elements were identified, including TGA-element/AuxRR-core (auxin), O2-site (zein metabolism), TCA-element (salicylic acid), ABRE (abscisic acid), GARE-motif/P-box/TATC-box (gibberellin), and CGTCA-motif/TGACG-motif (MeJA responsive element) (Figure 4). All *MtAKR* genes contained one to seven cis-acting elements related to response to stresses or hormones. ABA-responsive elements were detected in 23 *MtAKR* genes. Among them, drought responsive elements were detected in 11 common *MtAKR* genes, and 13, 13, 14, 17, and 23 *MtAKR* genes contained responsive elements for auxin, zein metabolism, gibberellins, salicylic acid, and MeJA, respectively. results indicate the potential for significant regulation of the expression of *MtAKR* genes in response to various environmental factors.

### 2.6. Phylogenetic Analysis of AKRs in M. Truncatula and A. Thaliana

**Figure 5:**
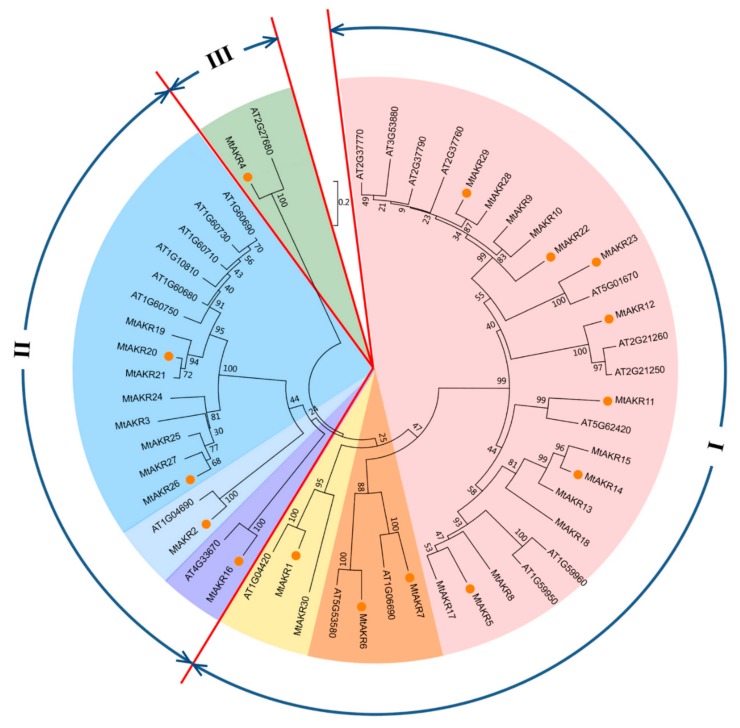
Phylogenetic tree of AKRs from *M. truncatula* and *A. thaliana*. The deduced full-length amino acid sequences were aligned by using ClustalW, and the phylogenetic tree was constructed by using the neighbor-joining (NJ) method in MEGA 7.0, with 2000 bootstrap replicates. Three groups and six subgroups are indicated. The genes marked with orange dots were subjected to expression analysis.

To analyze the evolutionary relationship of AKRs between *M. truncatula* and *A. thaliana*, an unrooted neighbor-joining tree was constructed in MEGA7.0, using the protein sequences of 22 AtAKRs and 30 MtAKRs (Figure 5).

Results showed clustering of the fifty-two AKRs into three groups by genetic distance (Figure 5). Groups I and II were each further divided into three subgroups (shown in different colors). Each group or subgroup contains at least one member from *A. thaliana* and one member from *M. truncatula*. Ten AtAKRs (AT2G37770, AT3G53880, AT2G37790, AT2G37760, AT2G21260, AT2G21250, AT5G62420, AT1G59960, AT1G59950, and AT1G06690) and fifteen MtAKRs (MtAKR5, MtAKR8-MtAKR15, MtAKR17, MtAKR18, MtAKR22, MtAKR23, MtAKR28, and MtAKR29) were included in the I-1 subgroup, suggesting that these twenty-five AKRs are closely related proteins. Two AtAKRs (AT5G53580 and AT1G04420) and two MtAKRs (MtAKR6 and MtAKR7) were included in the I-2 subgroup. One AtAKR (AT4G33670) and two MtAKRs (MtAKR1 and MtAKR30) were included in the I-3 subgroup. The II-1 and II-2 subgroups each contained one AtAKR and one MtAKR, with AT4G33670 and MtAKR16 in II-1, and AT1G04690 and MtAKR2 in II-2. Six AtAKRs (AT1G60750, AT1G60680, AT1G10810, AT1G60710, AT1G60730, and AT1G60690) and eight MtAKRs (MtAKR3, MtAKR19–MtAKR21, and MtAKR24–MtAKR27) belonged to subgroup II-3. Group III contains only two members (AT2G27680 and MtAKR4), which suggests that the sequences of these two proteins are significantly different from the other proteins.

### 2.7. Expression Profiles of the MtAKR Genes in M. truncatula Leaves and Roots under ABA, PEG, and NaCl Treatment

The expression levels of selected *AKR* genes were assayed by real-time PCR, to explore the potential response of *M. truncatula AKRs* to ABA, PEG, and NaCl stress. A total of fifteen *MtAKR* genes were randomly selected from each group or subgroup for PCR analysis. Expression analysis was carried out of samples of leaves and roots of *M. truncatula* plants exposed to ABA, PEG, or NaCl treatment.

**Figure 6:**
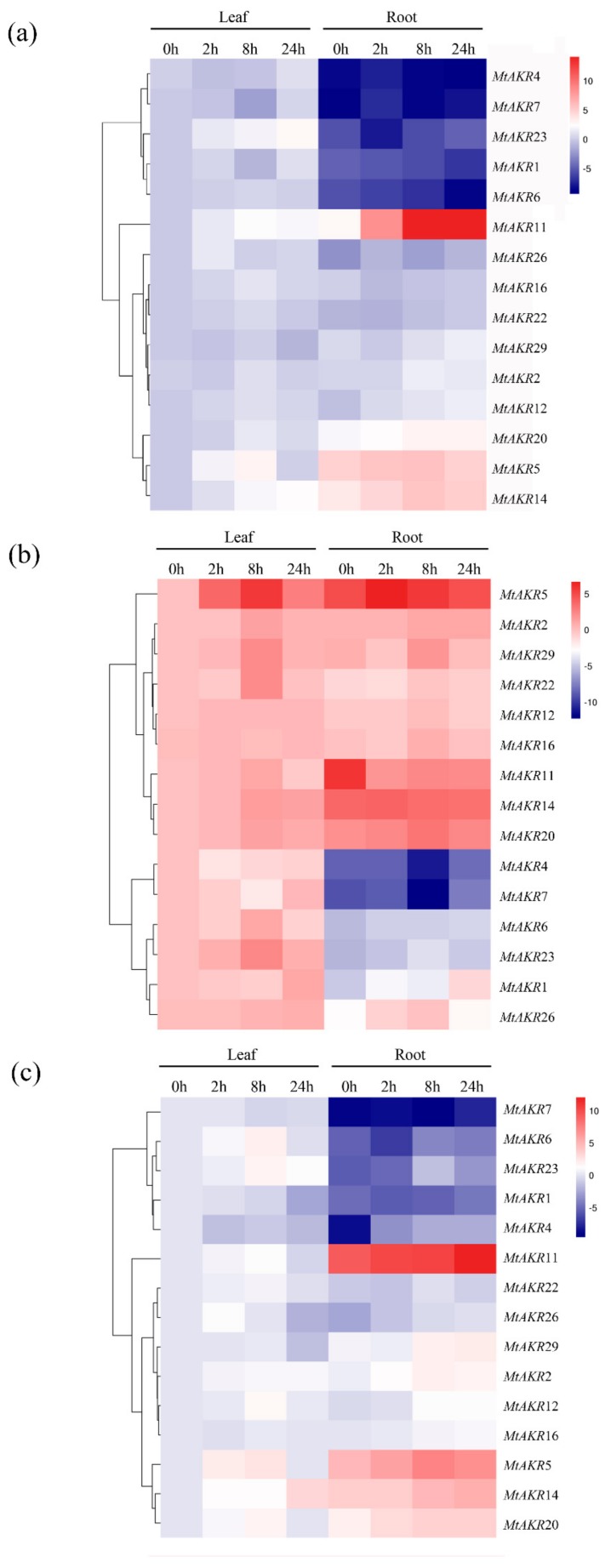
Hierarchical clustering and heatmap representation of leaf and root *MtAKR* genes expression profiles in ABA (**a**), PEG (**b**), and NaCl (**c**) conditions. The data obtained by quantitative RT-PCR correspond to the levels of *MtAKRs* in total RNA samples extracted from leaves and roots (four technical replicates). The expression levels of genes were used 2^−ΔΔ^*^Ct^*, and the heatmap was presented in log_2_ values.

The heatmap representation of the overall expression patterns revealed clustering of the *MtAKR* genes (Figure 6). Results indicated that most of the *MtAKR* genes were regulated to different degrees by ABA in both leaves and roots (Figure 6a). In general, the highest expression of each gene appears at 8 or 24 h after the start of ABA stress application. The fifteen expression patterns could be divided into two groups. Five genes (*MtAKR*1, *MtAKR*4, *MtAKR*6, *MtAKR*7, and *MtAKR*23) exhibited lower expression levels in roots than in leaves. Compared to 0 h, the expression levels of MtAKR1 and MtAKR6 decreased in roots. The other group included *MtAKR*2, *MtAKR*5, *MtAKR*11, *MtAKR*12, *MtAKR*14, *MtAKR*16, *MtAKR*20, *MtAKR*22, *MtAKR*26, and *MtAKR*29 genes, and most of them exhibited higher expression levels in roots than those in leaves. The expression levels of *MtAKR*5, *MtAKR*11, and *MtAKR*14 were significantly upregulated compared to 0 h in roots. This shows that these genes are more sensitive to ABA treatment in roots.

According to the expression pattern in plants subjected to PEG stress, the 15 *MtAKR* genes can be divided into three groups (Figure 6b). The first one included *MtAKR*2, *MtAKR*5, *MtAKR*11, *MtAKR*12, *MtAKR*14, *MtAKR*16, *MtAKR*20, *MtAKR*22, and *MtAKR*29 genes. Except for *MtAKR*11, the other eight genes displayed upregulated transcription in both leaves and roots. The highest expression level of *MtAKR*11 was detected at 0 h in roots. The expression of *MtAKR*5, *MtAKR*22, and *MtAKR*29 was significantly upregulated in both roots and leaves. The second group included *MtAKR*4 and *MtAKR*7 genes and exhibited downregulation at the transcriptional level in both leaves and root at 8 h. The remaining genes in leaves in the third group, including *MtAKR*1, *MtAKR*6, *MtAKR*23, and *MtAKR*26, exhibited significantly higher expression level than that in roots. PEG stress increased expression levels of these genes in leaves. In roots, *MtAKR*1 was significantly upregulated at 24 h.

As shown in Figure 6c, the expression patterns of the fifteen genes in response to NaCl treatment can be divided into two groups. The genes in the first group, which includes *MtAKR*1, *MtAKR*4, *MtAKR*6, *MtAKR*7, and *MtAKR*23, exhibited higher expressions in leaves than in roots. The responses were consistent with that treated by ABA in roots. *MtAKR*1, *MtAKR*4, *MtAKR*6, and *MtAKR*7 genes were downregulated in leaves but upregulated in roots at 24 h. The expression level of *MtAKR*2, *MtAKR*5, *MtAKR*11, *MtAKR*14, *MtAKR*20, and *MtAKR*29 in another group showed significant upregulation in roots.

Overall, expression analysis indicated that *MtAKR*5 and *MtAKR*14 were largely induced both in leaves and roots, and *MtAKR*1, *MtAKR*11, *MtAKR*20, and *MtAKR*29 were significantly induced in roots when seedlings were subjected to ABA, PEG, and NaCl treatment. Results showed that these genes mentioned above may play a vital role in resisting to abiotic stresses.

## 3. Discussion

The AKR superfamily contains 18 families and more than 190 annotated proteins, but only few AKRs have been studied in plants [11]. The AKRs were identified and named based on sequence similarity. Subfamilies contain proteins with more than 60% amino acid sequence, and members with less than 40% shared amino acid sequence were placed in different families [11,33]. Focusing on the structure and expression patterns of *AKR* family members which responded to salt, drought, and ABA stress in *M. truncatula* was helpful in identifying candidate genes involving these abiotic stresses. The genome sequence was mined to identify gene families, analyze genetic relationships, and determine gene family distribution across the chromosomes. Phylogenetic analysis used in this study can be performed to group genes into subfamilies and will facilitate analysis of gene function [34].

### 3.1. Characterization and Analysis of the MtAKR Gene Family

In the past 30 years, *M. truncatula* has become a widely studied model organism [35,36,37]. *M. truncatula* is always used to address biological questions by using genome sequencing and genomics approaches similar to *A. thaliana* and *Oryza sativa* [38]. The study of *M. truncatula AKR*s can provide a foundation for the study of legume forage *AKR* genes. The MtAKRs have an average of 336 amino acids and an average protein mass of 37 kDa in MtAKRs, which is consistent with the previous published that most AKRs contain approximately 320 amino acids, with proteins 33–37 kDa in size [8,39,40]. The differences in transcriptional induction can be attributed to the quality and numbers of the cis-elements present in the promoters [41]. The presence of a response element allows regulation of the gene expression levels by the corresponding transcription factors or proteins [42]. Abscisic acid or drought or defense and stress response elements were identified in five of the six *MtAKR*s most strongly induced by ABA, salt, and drought stress treatments (*MtAKR*1, *MtAKR*5, *MtAKR*11, *MtAKR*14, and *MtAKR*29; *MtAKR*20 was not found) (Figure 4). Two and four ABA response cis-elements were identified in *MtAKR*5 and *MtAKR*11. *MtAKR*20 is abundant in salicylic acid and MeJA responsive element (Figure 4), but was clearly induced by ABA, salt, and drought stresses in this study, plotted in Figure 6. Therefore, the response of this gene to these abiotic stresses may be indirectly regulated by other mechanisms. The presence of a large number of auxin and abscisic acid response elements in the promoters of *MtAKR*s indicate potential functions in plant growth and responses to environmental stresses [43].

### 3.2. The Phylogenetic Analysis of Close Relationship between A. thaliana and M. truncatula AKR Genes and MtAKRs Expression Patterns

In this study, 22 genes in *A. thaliana* (Simpson et al. reported that the *A. thaliana* genome includes at least 21 *AKR* genes [34]) and 30 genes in *M. truncatula* that putatively encode AKR proteins were identified. A phylogenetic tree was constructed to explore the phylogenetic relationships of *AKR* genes from *A. thaliana* and *M. truncatula*. The expression of *MtAKR* genes in different stresses is helpful to identify candidate genes for subsequent detailed characterization [43]. The phylogenetic analysis revealed that five MtAKRs (MtAKR9, MtAKR10, MtAKR22, MtAKR28, and MtAKR29) and four *A. thaliana* AKRs (AT2G37770, AT3G53880, AT2G37790, and AT2G37760) were grouped in the same cluster in subgroup I-1 (Figure 5). These four AtAKRs are the members of AKR4C subfamily and named AKR4C8, AKR4C9, AKR4C10, and AKR4C11. Analysis of the *A. thaliana* AKRs suggested the function in reduction sugar-derived reactive carbonyls, as well as showed upregulation under salt and drought stress in three days [34]. In this study, the expression of *MtAKR*22 and *MtAKR*29 was detected. Results showed that the highest expression levels of *MtAKR*22 and *MtAKR*29 were present at 8 or 24 h after salt, drought, and ABA stresses, both in roots and leaves (Figure 6).

AT1G59950 (AtA6PR1) and AT1G59960 (AtA6PR2) in subgroup I-1 were previously proposed to be related to the aldose 6-phosphate reductase pathway, and they both exhibited response to cold and salt stresses [40]. The sequences of MtAKR5, MtAKR8, and MtAKR17 are in the same cluster with these genes (Figure 5). These *MtAKR* genes contain only three or four exons (Figure 2) and the same motif in amino acid sequence (Figure 3). These suggest the similar function of MtAKR5, MtAKR8, and MtAKR17. The expression pattern of *MtAKR5* was detected in this study (Figure 6) had a similar response to that of AtA6PR1 under NaCl treatment [40].

AT1G04690 was reported as a K^+^ channel β subunit and is classified in the AKR6C subfamily [44]. It can be regulated by auxin, ethylene, and jasmonic acid [45]. McLoughlin and Yao [46,47] reported that AT1G04690 plays a role in salt-stress regulation. MtAKR2 is the only MtAKR clustered with AT1G04690 in subgroup II-2. The *MtAKR*2 promoter contains both auxin and MeJA response elements (Figure 4). Transcription of this gene can be influenced by exogenous NaCl, PEG, and ABA. MtAKR2 is therefore presumed to be a member of AKR6C subfamily and plays a role in salt-stress regulation.

Plant AKRs are regulated by many abiotic stresses, including drought, low temperature, and salt. However, a few AKRs have been studied, such as cadmium-stress-tolerance gene IbAKR [11], the drought-stress-tolerance gene MsALR [48], and the heat-stress-tolerance gene OsAKR [41]. OsAKR1 and EcAKR4-1 can detoxify glyphosate and increase herbicide resistance in plants [49,50]. PpAKR1 can be induced by ABA, salt, cold, and oxidative stresses [13]. AKR4C1 and AKR4C2 can be induced by ABA [51,52]. Therefore, AKRs are induced to protect plants from abiotic stress. Studies have been conducted to show that ABA in response to water stress is a cell-signaling process, and ABA content changes under both salt and drought stresses [53,54]. In this study, *MtAKR*5, *MtAKR*11, *MtAKR*14, and *MtAKR*20 showed significant upregulation in response to the ABA (Figure 6a) and NaCl (Figure 6c) treatments in both leaves and roots, indicating that these four *MtAKR*s may regulate *M. truncatula* response to osmotic stress in ABA-dependent manner. This conclusion is consistent with that reported by Tran [55]. *MtAKR*1, *MtAKR*6, and *MtAKR*7 exhibited similar responses to salt, drought, and ABA treatments (Figure 6). The gene expression of *MtAKR*1, *MtAKR*6, and *MtAKR*7 under ABA and PEG treatments showed upregulated with the induction of time and reached the highest value at 8 or 24 h in leaves (Figure 6a,b). The gene expression of *MtAKR*1 and *MtAKR*7 showed downregulated during the induction of time in salt stress. The highest value of these two *MtAKR*s in leaves occurred at 0 or 2 h (Figure 6c). The phylogenetic analyses showed that MtAKR1, MtAKR6, and MtAKR7 were in the same group (Figure 5). MtAKR1, MtAKR6, and MtAKR7 are predicted to locate in chloroplast (Table 1). The same-cluster genes in *A. thaliana* (AT1G04420, AT5G53580, and AT1G06690) were predicted to locate in chloroplasts, according to the UNIPROT online tool (https://www.uniprot.org/uniprot European Bioinformatics Institute, Cambridgeshire, UK). AT1G06690 and AT5G53580 genes were reported to be involved in the PLP salvage pathway and play a role in osmotic-stress resistance [56], indicating that *MtAKR*1, *MtAKR*6, and *MtAKR*7 may also be involved in resisting osmotic stress, as with drought and salt stress.

Some *AKR* genes have been considered as potential breeding targets for developing stress-tolerant varieties. The selection of candidate *AKR* genes is of great significance to the breeding of legume forages in the future. The characteristics identification and prediction of the selected 30 *MtAKR* members indicated that they are aldo–keto reductase superfamily members. The expression pattern of 15 *MtAKR* genes showed that *MtAKR*5, *MtAKR*11, *MtAKR*14, and *MtAKR*20 in roots under ABA and NaCl stress, and *MtAKR*1, *MtAKR*5, and *MtAKR*29 in both leaves and roots under PEG stress were induced significantly (Figure 6). These results suggest that the six genes can be further studied in *M. truncatula* as candidate genes for stress-resistance regulation.

## 4. Materials and Methods

### 4.1. Identification, Chromosomal Localization, and Preliminary Analysis of the AKR Genes in Medicago truncatula Genome

The members of the *M. truncatula* AKR family were identified by using the Pfam database (http://www.pfam.xfam.org/), by searching for the AKR domain (PF00248). The full-length protein sequence of each identified *M. truncatula* AKR family member was obtained from the phytozome database (https://phytozome.jgi.doe.gov/pz/portal). Functionally redundant sequences were removed by the online tool CD-HIT (http://weizhong-lab.ucsd.edu/cdhit_suite J. Craig Venter Institute, California, US), sequences with low similarity were removed (<40% similarity with major AKRs) by using ClustalW (v 2.1) (https://www.genome.jp/tools-bin/clustalw Institute for Chemical Research, Kyoto University, Kyoto, Japan), and sequences that lacked the AKR conserved domain were eliminated by searching the Ensembl Plant database (MedtrA17_4.0) (http://plants.ensembl.org/index.html).

### 4.2. Analysis of the Protein Features of the Medicago Truncatula AKRs

The CDS and genomic DNA sequence were downloaded from Ensembl Plant. General features of the AKRs, including their theoretical isoelectric point (*pI*) and molecular weight (mW), were predicted by using the ExPASy-Protparam tool (http://www.web.expasy.org/protparam/ The SIB Swiss Institute of Bioinformatics, Lausanne, Switzerland (appeared before)) [57]. The subcellular localization of AKRs was explored by using the TargetP (v 1.1) online tool (http://www.cbs.dtu.dk/services/TargetP/) [58].

### 4.3. Structural Analysis of the AKRs

The gene organization of the *AKR* genes was explored by the online tool GSDS (v 2.0) (Gene Structure Display Server) (http://gsds.cbi.pku.edu.cn/ Center for Bioinformatics, Peking University, Beijing, China(appeared before)) [59]. The full-length protein sequences were used to construct phylogenetic tree and performed the bootstrap neighbor-joining analysis in MEGA 7 software [60]. The parameters were taken as gap open penalty of 10.0, gap extension penalty of 0.2, and bootstrapping with 2000 replicates.

### 4.4. Motif Composition Analysis of MtAKR Proteins

The MEME 5.0.5 online program (http://meme-suite.org/index.html National Institutes of Health, Bethesda, US) [61] was used for motif identification of the 30 *M. truncatula* AKR proteins. The following optimized parameters of MEME were applied: Number of repetitions, any; maximum number of motifs, 10; and optimum width of each motif, between 6 and 50 residues. TB tools (v 0.6669) [62] and the online tool WebLogo (http://weblogo.berkeley.edu/) were used to reconstruct the motif composition and identify a logo sequence.

### 4.5. Prediction of Cis-Acting Elements in MtAKRs

Putative hormone-related, stress-response cis-acting elements were identified in promoter sequences by analysis of the 2kb of sequence upstream of the initiation codon ATG of each of the *MtAKRs* genes, using the PlantCARE database online tool (http://bioinformatics.psb.ugent.be/webtools/plantcare/html Laboratoire Associé de l’Institut National de la Recherche Agronomique (France), Universiteit Gent, Gent, Belgium) [63]. TB tools (v 0.6669) was then used for data visualization.

### 4.6. Phylogenetic of AKRs in Medicago truncatula and Arabidopsis thaliana

Sequences of AKRs in *M. truncatula* were collected from NCBI (http://www.ncbi.org/ National Center for Biotechnology Information, National Library of Medicine, Bethesda, US), using BLASTP analysis, with all of the *Arabidopsis* AKR protein sequences as queries. A confirmed AKRs extracted from *Arabidopsis thaliana*, Pfam (PF00248), was used to verify putative *M. truncatula* AKRs in the phytozome database. An unrooted neighbor-joining tree was constructed by MEGA (v 7.0). The parameters were taken as gap open penalty of 10.0, gap extension penalty of 0.2, and bootstrap analysis of 2000 replicates.

### 4.7. Plant Growth Condition and Treatments

Plump seeds of *M. truncatula* (wild type R108 from our lab: The Institute of Animal Science, the Chinese Academy of Agricultural Sciences) were scarified by sandpaper, surface sterilized with 75% alcohol for 10 min, washed five times with distilled water, and then germinated on plates with paper and distilled water. Seedlings (when seed cotyledons opened) were transferred to 1/2 Hoagland nutrient solution in a plant-growth chamber (GXZ-430C), with conditions of day 16 h, 24 °C, and dark 8 h, 20°C. The nutrient solution was changed every five days, and 25-day-old seedlings were then subjected to salt (200 mM of NaCl), drought (50 g·L^−1^ of PEG 6000), and ABA (100 µM) treatments for 0 (9:00 a.m.), 2, 8, and 24 h. Leaf and root were separately harvested and processed for RNA isolation and cDNA synthesis after the treatments. Four biological replicates (one plant per biological replicate) were used in this experiment.

### 4.8. RNA Preparation, cDNA Synthesis, and Expression Analysis by qRT-PCR

RNA extraction and cDNA synthesis were performed by using the Promega total RNA Kit and TaKaRa PrimeScript RT kit with gDNA Eraser, respectively. The specific primer pair for each *AKR* gene was designed by using Primer 5 software, and the primer sequences are listed in Table S1. The qRT-PCR was performed in an ABI 7300 instrument, with the following conditions: 95 °C for 10 min, 40 cycles of 95 °C for 15 s, and 60 °C for 60 s. All reactions were run in quadruplicate. Relative fold changes were calculated based on the comparative *Ct* method, and the *Mt*-*Actin*7 (*MTR3g095530*) gene was used as an internal standard. Heatmap representation was performed by using the normalized (2^−ΔΔ^*^Ct^*) value. The data of gene expression in qRT-PCR are listed in Tables S2 and S3. The data visualization was performed with OmicShare tools (http://www.omicshare.com/tools Gene Denovo, Guangzhou, China). Relative expression levels (FC) in roots and leaves of *Medicago truncatula* under ABA, PEG, and NaCl stresses are shown in Table S4.

## Figures and Tables

**Table 1:** The fundamental characteristics of candidate gene.

No.	Gene Name	gDNA Length ^a^	cds Length ^a^	Gene ID ^b^	Protein Length ^c^	Mass ^c^ (kDa)	PI ^c^	GRAVY ^c^	TargetP ^d^
1	MtAKR1	4822	1254	MTR1g042730	417	46.65	7.58	−0.251	C*
2	MtAKR2	5122	987	MTR1g047250	328	36.48	7.57	−0.251	M
3	MtAKR3	2646	1023	MTR1g102750	340	38.02	5.50	−0.250	_
4	MtAKR4	2510	1161	MTR2g085125	386	43.44	8.15	−0.300	M
5	MtAKR5	2116	948	MTR3g083130	315	35.16	5.70	−0.175	_
6	MtAKR6	5971	1536	MTR3g092140	511	56.96	9.20	−0.109	C*
7	MtAKR7	4149	1101	MTR3g449790	366	40.25	8.98	−0.303	C
8	MtAKR8	3157	951	MTR0374s0050	316	35.58	5.40	−0.184	C
9	MtAKR9	4958	942	MTR4g021350	313	34.77	6.10	−0.301	_
10	MtAKR10	5964	915	MTR4g021410	304	33.97	6.00	−0.290	_
11	MtAKR11	3166	948	MTR4g036845	315	36.00	5.40	−0.419	M
12	MtAKR12	3177	930	MTR4g072060	309	34.82	5.88	−0.213	_
13	MtAKR13	2235	948	MTR4g072320	315	35.84	5.96	−0.314	_
14	MtAKR14	1674	966	MTR4g072350	321	36.27	6.27	−0.260	_
15	MtAKR15	1742	966	MTR4g072360	321	36.55	6.42	−0.274	_
16	MtAKR16	3633	951	MTR4g092750	316	34.29	5.44	−0.096	_
17	MtAKR17	1494	939	MTR5g097910	312	34.89	5.93	−0.231	_
18	MtAKR18	2376	957	MTR6g073110	318	35.62	7.09	−0.160	_
19	MtAKR19	1220	765	MTR7g021670	254	28.05	5.90	−0.321	_
20	MtAKR20	3713	1020	MTR7g021680	339	37.40	5.77	−0.285	_
21	MtAKR21	2867	1020	MTR7g021850	339	37.66	5.60	−0.318	_
22	MtAKR22	3024	1065	MTR7g063580	354	39.51	6.46	−0.106	S*
23	MtAKR23	2904	966	MTR7g070500	321	36.20	6.02	−0.462	_
24	MtAKR24	3877	1032	MTR7g114970	343	37.95	6.02	−0.181	_
25	MtAKR25	3171	1053	MTR7g114980	350	38.36	6.13	−0.222	_
26	MtAKR26	3134	1056	MTR7g114990	351	38.35	5.64	−0.165	_
27	MtAKR27	2080	996	MTR7g115010	331	36.33	5.25	−0.191	_
28	MtAKR28	2713	960	MTR8g070095	319	35.69	5.73	−0.206	_
29	MtAKR29	3165	948	MTR8g070115	315	34.92	6.46	−0.242	_
30	MtAKR30	3848	984	MTR8g088160	327	36.83	6.71	−0.223	M*

^a^ The length of gDNA and coding sequence were acquired from Ensembl Plant (http://plants.ensembl.org/index.html European Bioinformatics Institute, Hinxton, Cambridgeshire, UK.). ^b^ The protein IDs were obtained from phytozome (https://phytozome.jgi.doe.gov/ U.S. Department of Energy, Washington, US). ^c^ Protein length, mass (molecular weight, kDa), *PI* (isoelectric point), and the grand average of hydropathy value (GRAVY) were analyzed by using the ExPASy-Protparam tool (http://www.web.expasy.org/protparam/ The SIB Swiss Institute of Bioinformatics, Lausanne, Switzerland). ^d^ Subcellular localization of the MtAKRs was predicted by using TargetP (v 1.1) (http://www.cbs.dtu.dk/services/TargetP/ Department of Health Technology, Lyngby, Denmark).C:Chloroplast. S: Secretory pathway; M: Mitochondria; _: Any other location; superscript * indicates highly reliable prediction (reliability class, from 1 to 5, where 1 means the strongest prediction. RC is a measure of the size of the difference (‘diff’) between the highest (winning) and the second highest output scores. There are 5 reliability classes defined as follows: (1) diff > 0.800; (2) 0.800 > diff > 0.600; (3) 0.600 > diff > 0.400; (4) 0.400 > diff > 0.200; (5) diff < 0.200. Thus, the lower the value of RC, the safer the prediction. Superscript * denotes the value of diff is 1).

**Table 2:** Amino acid composition and function of different motifs.

Motif Number	Length (Amino Acid)	Best Possible Match	Function
Motif 1	21	CEASLKRLQLDYIDLYYIHWP	Aldo/keto reductase domain
Motif 2	29	CTPAQIALRWGYQQGHDVCPKSFNTERMN	unmatched
Motif 3	28	ISAIHHAIKAGYRHFDTADIYGNHEENG	Aldo/keto reductase domain
Motif 4	29	PAVNQVEMHPSWHQDKLREFCNQKGIHVS	unmatched
Motif 5	50	FKAEDMTPFDMKGTWKAMEECYRS GLARAIGVSNFSIKKLQDLLEYARIP	Aldo/keto reductase domain
Motif 6	50	IRRAHAVHPITAVQMEWSLWTRDIE EEIIPLCRELGIGIVPYSPLGRGFF	Aldo/keto reductase domain
Motif 7	21	NRDDLFITSKLWCTDHHPEDV	unmatched
Motif 8	21	QNIGAFDWKLTQEDMRKISQI	unmatched
Motif 9	29	PRMKLGTQGMEVSKQGFGCMGMSGFYNPP	unmatched
Motif 10	29	DTSVPIEDTMGELKKLVEEGKIKYIGLSE	Aldo/keto reductase domain

## Supplementary Materials

The following are available online at https://www.mdpi.com/1422-0067/21/3/754/s1. Figure S1: The logo of ten *MtAKRs* motif construction. Each logo consists of stacks of symbols, one stack for each position in the sequence. The conservation at each position is indicated by the overall height of the stack. The height of symbols within the stack is proportional to frequency of each amino acid at that position. Table S1: The qRT-PCR primers of *MtAKRs*. Table S2: The data of gene expression in qRT-PCR (leaf). Table S3: The data of gene expression in qRT-PCR (root). Table S4: Relative expression levels (FC) in roots and leaves of *Medicago truncatula* under ABA, PEG and NaCl stresses. Material S1: The sequences of *MtAKR* genes’ CDS and gDNA.
