# Supplementary material for: Analysis of Aldo–Keto Reductase Gene Family and Their Responses to Salt, Drought, and Abscisic Acid Stresses in Medicago truncatula
Source: Int J Mol Sci. 2020 Jan 23;21(3):754. doi: 10.3390/ijms21030754 (PMC7037683; doi:10.3390/ijms21030754)
Supplement: Supplementary file 1 [file ijms-21-00754-s001.zip › Material S1.docx]

MtAKRS' CDS sequences:

>Medtr0374s0050

ATGTCTGCTTCAAAAATCCCTCAAGTAGTGCTGAAATCTTCATCCAACCAATCCAACATGCCAGTGATAGCATTTGGTACAGCAGCTGTAACAAACAACGATGGAGAAATCACAAAAGTAGCAGTAATTGAAGCTATCAAATCAGGTTACAGACACTTTGATACTGCATCAATATATGGCTCAGAAGAGGCTTTAGGAGAAGCTATAGAAGAAGCTCTTCAACTTGGTCTAATTGGATCAAGAGATGAACTCTTTATCACCTCTAAACTTTGGGTTACTGATAATTTTCCTCATCTTGTTCTTCCTGCTCTTCAGAAATCACTCCAGACCCTGAAATTAGAATATTTGGACCTTTATTTGATCCACTGGCCAATCAGTGTGAAGCCTGGAAATTGGGAATTGCCGTATGCTGAAGAGTTAATAACCACATTTGATTTAAAAGGAGTGTGGACATCAATGGAAGAATGCCAGAAACTAGGCCTTACAAAATATATAGGAGTCAGTAACTTCACTCGCAAGAAACTCGAAGATCTACTCTCATTTGCTATCATTCCTCCTTCAGTGAATCAAGTGGAGATGAATCCAGTTTGGCATCAGAAGAAGCTAAAAGAATACTGTGAAGCAAAGGGTATAATCATAACTGCTTTCTCTCCTTTAGGAGCCAAAGGGGCCAGTTGGGGTAGTAATGAAGTTATGGACAGTGAAATACTCAAGCAAATTGCAGAGGAACATGGCAAAAATATTGCTCAGGTATGTCTTAGATGGTTGTATGAGCAAGGGGTGACTATGGCTGTGAAGAGCTACAACAAGGAGAGAATGAAGCAAAACATGGAAATATTTGATTGGTCACTTGCAAAAGATGATCATGAAAAGATTGATCAAATCAAACAAATTCGTGTCAACAATGGGCCAGTTGTGTTCATTCCTAACCTTTGGGATGGGGAAACTTAA

>Medtr1g042730 ATGGCCGGTGTCTGCTCTTTCTTCTCTACCACTTCCTCTGACGTTGTTGCCATTTCACATTCTCTTACACCATCACTGTCTCTCAACAACACTACACTTTCAACAACAAGTAAAAGAAGATGCATTCTGAGGAGGACGAGAACTGTGAGGCCTCTTGTTTGTGCAGCCATCAACAATCCATTACAATACCGGAAACTTGGTGACTCTGACCTTAATATCAGTGAAATCACTCTTGGTACTATGACCTTTGGGGAGCAAAACACAGAAAAAGAATCTCATGACATACTCAATTATGCATTTGAGAATGGCATTAATGCTCTTGATACTGCTGAGGCATACCCGATTCCAATGAAGAAAGAGACACAAGGAAAAACTGATCTCTATATAGCTAGCTGGCTGAAATCTCAATCTCGTGACAAGATTATCATAGCAACAAAAGTTTGTGGTTATTCTGAGAGATCGAGTTACTTACGCGACAATGCAGACATTTTGCGGGTTGATGCGGCAAATATCAAAGAAAGTGTGGAGAAAAGTCTTAAGCGTCTTGGGACTGATTATATTGATTTGTTGCAAATTCATTGGCCAGATCGCTACGTTGCACTATTTGGTGAATATTCTTATGATCCTTCAAAATGGAGGCCTAGTGTGCCATTTGTTGAACAGTTGCAAGCTTTTCAAGAACTTATCAATGAAGGAAAGGTACGTTACCTAGGTGTTTCAAATGAGACTTCATATGGAGTGATGGAATTCGTCCATGCTGCTAAAGTTGAGGGACTTCCAAAGATTGTCAGTATCCAAAACAGCTATAGCTTGCTCGTAAGAAGTCGTTTTGAAGTTGATCTTGTGGAGGTTTGTCATCCAAAGAATTGTAACATTGGCTTGCTGTCCTATTCCCCACTAGGCGGCGGAACACTCTCAGGAAAATATATAGATATAAATTCTAAAGCTGCAAAAAGTGGAAGATTGAACCTCTTCCCTGGCTACATGGAAAGATATAACCAATCGATTTCACGGGAAGCAACTATTAAGTATATTGAGTTGGCCGAGAAACATGGTCTAACTCCTGTCCAGCTCGCGCTTGGATTTGTGAGAGATCGTCCATTCATGACGAGTTCAATTATCGGTGCAACTTCTGTGGACCAACTAAAAGAAGACATAGATGCTTTTACAACAACCGAACGACCTTTGCCAGCAGAAGTCATGACAGGAATTGAAGCTATCTTCAAGAGATACAAAGATCCTAGCATCCTTTAA

>Medtr1g047250 ATGCAATACAACAACTTAGGCCGGTCAGGTCTAAAAGTCAGCCAACTCTCCTACGGAGCATGGGTAAGTTTCGGCAACCAGCTCGACGTCAAAGAGGCAAAATCTCTTCTCCAATGCTGCCGCGACCACGGCGTAAACTTCTTCGACAACGCAGAAGTCTACGCCAATGGTCGCGCAGAGGAGATCATGGGGCAGGCAATCAAAAGCCTTGACTGGAAACGGTCTGACATCGTTGTTTCGACAAAGATCTTCTGGGGAGGACAAGGACCGAATGATAAGGGGCTGTCAAGGAAGCATATTGTGGAAGGGACGAAAGCGTCGTTGAAGAGGCTGGGGATGGAGTATGTGGATGTTGTTTATTGTCATAGGCCGGATGTTTGTACACCGATTGAGGAGACTGTAAGAGCAATGAATTTTGTTATTGATCAAGGTTGGGCTTTTTATTGGGGGACTAGTGAGTGGTCTAGTCAGCAGATTACTGAAGCTTGGGCTGTTGCTAATAGGTTGGATTTGGTTGGACCGGTTGTTGAACAGCCTGAGTATAACCTCTTGAACAGGCACAAGGTTGAGTCTGAGTATCTTCCCCTGTATTCTAGTTATGGAATTGGTCTCACCACTTGGAGTCCACTTGCGTCTGGAGTCCTTACTGGAAAATACAAGAAAGGAGTTATTCCCCCAGACAGCCGTTTTGCTTTGGAAAATTACAAGAATCTTGCATCTCGATCACTGGTTGATGATGTGCTCAAAAAGGTTGATGGTCTGAAGCCAATAGCAGATGAACTTGGTGTGCCATTAGCACAACTCTCAATTGCATGGTGTGCTGCTAATCCTAATGTTTCGTCAGTTATCTGTGGCGCTACAAAAGAATCTCAGATTCATGAGAATATGAAGGCTATTGAAGTCGTTAAACTACTAACTCCTGCTGTGATGGAGAAGATTGAGGCTGTTGTTCAAAGCAAACCAAAGCGCGCTGATTCATACAGATGA

>Medtr1g102750 ATGGAGATACAAGTCCCAAGAGTCAAACTGGGTACCCAAGGTTTAGAGGTGTCTAGGTTGGGATTTGGATGTGGAGGATTATCTGGAATTTATAATGCTCCTCTCTCACATGAAGATGGATGTTCAATCATTAAGGAAGTATTCAATAGGGGAGTTACCTTTTTTGATACATCAGATCTCTATGGACACAAACATGATAATGAAATCATGGTTGGTAAGGCATTAAAAGAACTTCCCCGCGAAAAAGTACAATTGGCTACGAAGTTTGGCGTTACTTTATCTGATGAGATGGTTTTGGGGGTAAAAGGTACCCCTGAATATGTGAGGGAGTGCTGTGAAGCTAGTCTGAAGCGGCTTGATGTCAAGTATATTGATTTATACTATCAACACAGAGTTGACACTTCAATTCCAATTGAAGACACCATGGAGGAACTCAAACAACTTGTAAATGAAGGAAAGATAAAATATATCGGGTTGTCAGAGGCTAATGCTGATACTATAAAGAGAGCACATGCAGTTCATCCTATTACTGCTTTGGAAATGGAGTATTCCTTATGGTCCCGTGACATTGAAGAAGAAATAATTCCACTCTGCAGAGAGCTTGGCATTGGAATTGTAGCATACAGCCCCCTTGGTCGCGGCTTTTTTGCAGGAAAGTCAGTGGTAGAGGCTTTGTCTAGTCAGAGCTTGCTGGCTATGCATCCAAGGTTCACAGGAGAAAATTTGGAAAAGAACAAGCTTTTTTATGAACGAATCAATGACTTGGCTTCAAAGCATGCATGCACTCCTTCTCAATTAGCATTAGCTTGGCTTCTGCATCAGGGAAATGACATAATCCCTATCCCTGGGACTACTAAACTGAAGAACTTTGAAAACAACATTGGATCTCTGAATGTGAAGCTTACACAAGAAAATTTGAAGGAAATTTCTGATGCAGTTCCCGTTGAGGAAATTGCTGGGGAGCGAGAATATGATAGTATATCACAATATCTTTGGAAGTTTGCAACTACGCCGCCAAAGTAA

>Medtr2g085125 ATGACTGGGTTATTGCAGCATTCTCTGAGCAAATTCACTTATTACAATTCAAACAGAACACCAACAAGAAGAGGAATTGTAACAAGAGAATATAAGCATTTGAGACCATGTCAGTGTGTGACTACCCAGGAGGATAATCGGCGCATTGTGGTCAGTAATGGGAAAGATTCATTGGATATAACTCGGGTTGTGAATGGAATGTGGCAAACGAGTGGTGGATGGGGGAAAATTGATAGGGATGAGGCTGTTGATTCTATGCTTAAATACGCTGATGCTGGATTAACCACTTTTGATATGGCGGATATATATGGGCCAGCTGAAGATCTATATGGGATTTTTATCAATCGAGTTCGTCGTGAGCGTCCACCAGAATTTTTGGAACAGGTCAGAGGTCTCACTAAGTGGGTGCCACCGCCAGTTAAGATGACAAGTAGCTTTGTAAGAGACAACATTAATGTGTCAAGGAAGAGAATGGACGTGGAATCCTTGGATATGCTTCAGTTCCATTGGTGGGATTATTCAAATTCAGGATACCTTGATGCACTAAAACACCTCACAGACTTGAAAGGAGAAGGTAAAATCAAAACTATAGCTTTAACAAATTTTGATACCGAGAGATTACAAATTATTCTTGAAAATGAGATTCCCATTGTGAGCAATCAGGTACAACATTCACTTGTTGATATGCGTCCCCAGCAGAAAATGGCAGAGCTTTGTCAGCTTACAGGAGTCAAACTGATAACGTATGGGACAGTAATGGGTGGTCTGTTGTCTGAGAAGTTCCTTGACACCAACATAAACATCCCTTTTGCTGGACCTGCAATAAACACTCCATCCCTCTCAAAATACAAAAGGATGGTGGATGCTTGGGGAGGATGGGGTTTGTTCCAAGGACTTCTTCGAACTCTTAAACAAGTATCTACTAAGCATGGTGTTTCCATTGCAACTGTTGCTGTGAAGTATATACTTGACCAGCCTGCTGTAGCAGGATCAATGGTTGGTGTAAGACTTGGCTTGTCAGAACATATACAAGACTGCAATGCTATCTTTTCACTTGTTCTTGATGAAGAAGATGTAAACAGCATAAGGGAAGCAGCAGGGAAAGGCAAGGATCTTCTTAAAGTGATTGGTGATTGTGGAGATGAGTACAGGCGTGCATGA

>Medtr3g083130 ATGGCTGCTACACCTACAGTCCCTGAAGTAGTCCTCCCCTCCTCCACCGGACAACGGAAAATGCCGGTGATGGGCCTCGGCACGGCACCCGAAGCAACCAGTAAGGTTACTACAAAAGATGCTGTCCTTGAAGCCATCAAACAAGGTTATAGACATTTTGATGCTGCTGCTGCATATGGTGTGGAAAAATCTGTTGGAGAAGCTATTGCAGAAGCACTTAAACTTGGACTTATTTCATCTAGAGATGAACTCTTTGTTACTTCTAAATTATGGGTTACTGATAATCATCCTGAGCTCATTGTTCCTGCTCTGCAGAAATCTCTAAGGACTCTTCAACTAGAAAACTTAGACCTCATTTTGATCCATTGGCCAATTACTACTAAACCAGGTGAAGTTAAATACCCTATTGAAGTATCAGAGATTGTGGAATTTGATATGAAAGGTGTGTGGACATCATTGGAAGAATGTCAAAAACTTGGTCTCACGAAAGCCATTGGAGCCAGCAACTTCTCAATCAAGAAGCTTGAAAAATTGCTATCCTTTGCAACCATCCCTCCTGCTGTGAATCAAGTGGAAGTCAACCTTGGATGGCAACAAGAGAAACTTAGAGCCTTCTGCAAGGAAAAGGGTATTGTGGTAACTGCCTTCTCACCCCTTAGGAAGGGTGCTAGCAGGGGTGCTAATTTAGTGATGGACAATGATATACTCAAAGAATTGGCTGATGCTCATGGCAAGACTATAGCTCAGATTTGTCTTCGATGGCTATATGAGCAAGGATTGACTTTTGTGGTTAAGAGCTATGACAAGGAGAGGATGAACCAAAACTTGCAAATCTTTGATTGGTCATTGACTGAGGATGATTACAAGAAAATTAGTGAAATCCATCAAGAGAGACTCATCAAAGGACCAACCAAGCCTCTTCTTGATGATCTATGGGATGAAGAATGA

>Medtr3g092140 ATGGCACTCTCATCTTGCTGCAGTTTTGTTGTTTGTTCCTCAAACATTAACAACCCTTTACCATCTTTTACTTTCAAATTCCCTTCCCTATGGCCATCCACCCAAAACCAAAAGCTCAAGATGGGGCCTCTAAGTGTTTCTCCAATGGGTTTTGGAACTTGGGCATGGGGTAATCAGCTTCTTTGGGGGTATCAAGAATCCATGGATGATGACCTACAGCAAGTGTTTAACATGGCTGTTGAGAATGGTGTTAATCTTTTTGATACTGCTGATTCTTATGGTACTGGAAGGTTTAATGGTCAGAGTGAGAAACTTCTTGGGAAGTTCATTCGAGATTTTCGAGAGAAAAAAGGGAGCCAGAATGAAATTGTGATTGCAACAAAATTTGCTGCTTATCCATGGCGCCTTACACCAGGGCAGTTTGTCGATGCTTGCAGGGCATCACTAGATAGGATGCAGATTGAGCAAATTGGGATAGGACAACTGCATTGGTCAACTGCAAATTATGCTCCTTTTCAAGAATTAGCTCTTTGGGATGGTTTAGTGGCAATGTATGATAAGGGTTTAGTTCAAGCTGTTGGAGTAAGTAATTATGGACCAAAGCAGCTTTTGAAGATACATGATTACTTGAAAGACCGCGGAGTTCCGTTATGCTCAGCCCAGGTGCAATTTTCTTTGCTAAGCATGGGGGAAGATCAACTAGAGATCAAGAGTATATGTGATTCTTTAGGTATTCGCGTAATTGCTTATAGTCCTCTAGGACTAGGAATGCTTACTGGGAAATACTCAGCATCCAAACTTCCAACTGGACCAAGGGGATTGCTATTTAAGCAAATACTTCCTGGACTGGATCCTTTATTGAGTTCCCTGAGAGATATTGCAAATAAAAGACGCAAAACCATGTCACAGGTTGCAATAAATTGGTGCATATGCAAAGGTACAATTCCAATTCCTGGAGTCAAGTCAATAACACAAGCAGAGGAAAATTTGGGTGCTCTGGGTTGGCGTCTCTCCTCAAACGAGCTACTTCAGTTGGAGGATGCAGCACAGCAATCGCCTCGCAGGATGATCCAAAACATTTTTCAAACCAGGTGGTTCAAAGCAATTTTATCGGTGGAAATCGAATTTGGTACCTCGAAGTTTACAACATACATACATATATTGTGCGCTAGACCTTCGTGGTGGTTTAAGGCGTTGAATACACTGGTTGCTTCATTTTGCCTATTTCTATCCGATAATTTATTTTCCTCACAATCATCTGAAAGGAGCATTCCAGCTACCAAAATCTATAGTTATCACATTAAGCATGCGCATGGTTACAGTTCCATCTACTTTTGCAAAGGTACAACTCTAATTCCTGGAGTCAAGTCAATAGCAAAGGCAGAGGAAAATTTGGGTGCTCTTGGTTGGCGTCTTTCATCAGACCAGCTGCTTCAGTTGGAGTATGCAGCACAGAAGTCACCTCCCAGGATTAAAGGATTTTCCAAACCTTCAGTTGGAGCTGCTTGGAGACAAAACATTTTCCAAACCATGTGA

>Medtr3g449790 ATGGCTCTTCATATAAACAGTGGTTGTTTTACTGTGATGGGTCACTGTAGAGTTCAAAGAGTAAAAGCTGTTGCTTCAGAGGGTTCTGCTAATGTTACAGTTGAAGATAAGTTGAAATTGGGTGGTTCTGATTTGAAGGTGTCAACTATTGGAATTGGAGCTTGGTCTTGGGGTGATACTACTTACTGGAACAATTTTCAATGGAATGATAGGAATGAGAAAGCTGCTAGAGATGCATTCAATACAAGTATTGATGGGGGTCTAACCTTTATCGACACAGCTGAAGTTTATGGCTCTGGGCTCGCTTTCGGAGCTGTAAATTCAGAAACTCTTCTAGGAAGATTTATTAAAGAACGAAAACAAAAGGATCCAAATGTTGAAGTTGAGGTTGCGACCAAGTTTGCTGCACTACCATGGAGATTAGGCCGTGAAAGTGTTCTAAGTGCACTTAAAGATTCCCTTGATCGACTCGAGATGACTTCTGTGGATCTGTATCAGCTTCATTGGCCTGGAGTATGGGGAAATGAAGGGTATATTGATGGTCTAGGTGATGCTGTTCAAAAAGGACTTGTAAAGGCTGTTGGTGTTTCAAACTATAGTGAAAACCGACTACGCGAAGCATATAAACAGCTCAAGAAGAGAGGTATTCCATTGGCTTCAAACCAAGTAAACTACAGCCTCATTTATAGGGTCCCTGAAGAAAATGGTGTGAAGAAAGCCTGTGATGAACTTGGGATTACATTGATTGCATATTCACCAATAGCTCAAGGTGTTCTTACTGGAAAGTATACTCCACGAAATCCTCCATCTGGGCCTCGAGGTAGAATTTATACTCCAGAATTCTTAACAAAGCTTCAACCACTGCTAAAGAAGATCACTGAAATAGGAGAGAAATACAATAAAACTTCTACACAGGTATCACTGAACTGGCTGGTAGCTCAAGGAAATGTTGTACCAATTCCTGGTGCTAAGACAGCAGAACAAGCTGAAGAATTTAAAGGTGCATTGGGATGGAGATTGACTAATGAAGAGGTAGCTGAGCTACGAAGTTTGGCGTCGAAAATTAAGCCTGTTATTGGCTTTCCTGTTGAAAAACTCTGA

>Medtr4g021350 ATGGCCACAGCAATCAAATTCTTTCAGTTGAACACTGGTGCTAAAATCCCTTCTGTTGGTTTAGGTACTTGGCAAGCTGAACCCGGTGTTGTTGCCAAAGCTGTCACCACTGCTGTTCAGGTTGGATACAGACATATTGATTGTGCTGAAGCTTATAAGAATCAATCAGAGATTGGTTCAGCTCTCAAAAAGCTTTTTGAGGATGGTGTGATTAAGCGCGAGGAATTATGGATCACCTCGAAACTATGGTGTTCAGATCATCATCCAGAAGATGTGCCAAAAGCATTGGATAAAACTTTAAATGATTTGCAACTTGATTACCTTGACCTCTATCTGATCCACTGGCCAGTGAGCATGAAAAGGGGAACAGGTGAATTTAAGGCTGAAAATCTCGATCGTGCAGACATACCAAGCACATGGAAAGCATTGGAAGCACTATATGACTCTGGCAAGGCTAAAGCTATAGGAGTCAGCAATTTCTCTACAAAGAAACTTCAAGACTTGTTGGATGTAGCAAGAGTGCCTCCAGCTGTTAATCAGGTGGAATTGCACCCTGGATGGCAGCAGGCAAAGTTGCATGCATTTTGTGAATCTAAAGGAATTCATGTATCTGGATATTCTCCTCTAGGCTCACCAGGAGTACTCAAAAGTGACATTCTTAAGAATCCAGTTGTCAAAGAAATTGCAGAGAAATTAGGGAAGACACCAGGACAAGTTGCCCTTCGATGGGGTCTACAAGCAGGACACAGCGTGCTACCTAAGAGCACTAATGAGGCTAGGATCAAGGAAAATCTTGACGTATACGATTGGTCGATTCCGGAAGACCTGTTTCCTAAGTTTTCTGAAATTAACCAGGATAAGCTAATCAAGGGTACCTTTTTCGTTAACGACACCTATGGTGCTTTTAGGACCATCGAAGAACTCTGGGATGGTGAAGTATGA

>Medtr4g021410 ATGGGAAGTGAGGATCTGAGATTGTTTGAATTGAACACAGGAGCTAAGATTCCATCTGTTGGTTTAGGCACTTGGCTTGCTGCACCTGGTGTTGTCTATGATGCTATATCCACTGCTGTCAATGTTGGTTATCGTCATATAGATTGTGCTCAAATTTATGGCAATGAAAAGGAGATTGGTGATGCCTTGAAGAAGTTGTTTGCTAATGGAGTAGTTAAGCGTGAAGAGATGTGGATCACGTCCAAGCTATGGTGTACTGATCACTTGCCAGAAGACGTCCCAAAGGCATTTGATAGAACTTTACGCGATCTGCAGCTGGACTATCTTGATCTCTACCTTATTCACTGGCCAGTGAGCATAAAAAATGGACATCTTACAAAACCTGACATACCAAGCACATGGAAAGCAATGGAGGCACTCTATGATTCCGGAAAGGCTCGAGCTATAGGAGTCAGCAATTTCTCTGTAAAGAAGCTTCAGGATCTGTTGGATGTGGGACGTGTGCCTCCAGCCGTTAACCAAGTGGAATTGCATCCTCAATTGCAGCAGCCAAATTTACATACTTTCTGTAAATCCAAGGGAGTGCACTTATCTGCTTATTCACCACTAGGCAAAGGACTTGAGAGCAATATTCTTAAAAATCCGGTTCTGCACACGACTGCTGAGAAATTAGGGAAGACTCCGGCCCAAATAGCCCTTCGATGGGGACTACAAATGGGTCACAGTGTACTTCCAAAGAGCACAAATACAGCGAGGATTAAAGAAAACATTGATATATTTGACTGGTCTATACCAGAAGATTTGCTAGCTAACTTCAATGAATTTCAGCAGGAAAGAGTAGTTCCAGGCGAACAATTTGTTAGTCAGACATCTCCTGGTTACAAAACAATTGCAGAACTCTGGGATGAAGAGTAA

>Medtr4g036845 ATGAGGTGCAATCATGTGCGTTTGAATTGTGGCATTACAATGCCTCTCATTGGATTTGGCACCTATTCCTACCCAAATGATAAGAAGACAACAGAAATTGCAGTCCACAATGCCCTTGAGGTGGGTTATAGACATTTTGATACAGCAAAAATTTATGGTTCTGAGCCAGCATTAGGCAATGCCATAAATAAGGCAATCTACAGGGGAGAAGTAGAAAGGGAAGATATTTTCTTGACATCCAAATTGTGGGGAAGTGATCACCATGATCCTGTTGCTGCATTGAAACAAACTCTAAAGAATCTGGACATGGACTATTTGGATATGTACCTAGTGCATTGGCCTGTAAAGTTAAAACCATGGGTTAACTACCCTGTTCCTAATGAAGATGACTTTGAAAATCTTGATCTTGAGACAACATGGGTAGGGATGGAAAAATGTCTTGAAATGGGGTTGTGTAGGTCTATTGGAGTTAGTAATTTCTCTAGCAACAAGATTGAGTGGCTCTTAGATTTTGCTAATACACCTCCAGTTGTTAATCAGGTGGAAATGCATCCTATGTGGAGGCAGAGAAAGCTAAGAAAGACATGTGGAGAGCACAAGATCCATGTAAGTGCCTACTCACCACTTGGTGGACCAGGAAATGCATGGGGATCTGCTGCTGTTGTTAATCATCCAATTATACAATCAATTGCCTTCAAACACCAAGCAACTCCAGCACAGATTGCATTGAAATGGGGACTATCAAAGGGCTCAAGTGTGATTGTGAAAAGTTTCAATGAAGAAAGAATGGAGGAAAATATGGGATCATTTGATCTAAACTTGGATGATGAAGATATCTTTGAGATTGAAAAATTGGAAGAAATGAAGATTATGAGAGGGGAATTTCATGTTAATCAAACCACAAGTCCCTACAAAACAATTGAAGAACTTTGGGATGATGAAATTTGA

>Medtr4g072060 ATGGCAATCACACTGAACAGCGGATTCAAGATGCCAATCATTGGACTTGGAGTTTGGCGCATGGAAGGACAAGCAATCAAAGACTTAATTATCAATTCCATAAAAATCGGTTATCGTCATTTTGATTGTGCTGCTGACTACAAGAACGAAGCAGAAGTTGGAGAAGCACTTAAAGAAGCTTTTGACACTGGACTTGTGAAGAGGGAGGATCTTTTCATTACCACCAAGCTTTGGAATTCTGATCATGGACATGTTGTTGAGGCTTGCAAAGATAGTCTTAAGAAGCTTCAGTTAGATTATCTGGATTTATATCTTGTTCACTTTCCTGTAGCCACTAGGCACACTGGGGTTGGTACTACTGATAGTGCTTTGGGTGAAGATGGGGTGCTGGACATAGATACAACCATATCCCTGGAAACTACCTGGCATGCGATGGAAGGCCTTGTTTCATCGGGCTTGGTTCGCAGCATAGGAATCAGCAACTATGACATCTTTCTGACTAGAGATTGCTTAGCATATTCCAAGATAAAGCCTGCTGTAAATCAGATTGAAACTCATCCATACTTCCAGCGTGAGTCTCTAGTCAAATTTTGTCAGAAGCATGGAATTTGTGTAACAGCCCACACTCCACTTGGAGGTGCTGCAGCAAACAAAGAATGGTTTGGTACAGAGTCATGTTTGGATGAGCAAATTCTCAAAGGTCTAGCTGAAAAATACAAAAAGACTGCTGCCCAGATTTCTCTTCGCTGGGGGATTCAAAGGAACACTGTTGTCATTCCTAAAACATCAAAACTGGAGAGATTGAAAGAGAACTTCCAGGTATTTGATTTTGAGTTGTCTAAAGAGGACATGGAGCTCATCAGCAGTATGGACAGGGAATATAGAACTAATCAACCGGCCAAGTTTTGGGGCATAGATCTTTATGCATGA

>Medtr4g072320 ATGGAAACAAAGAAAGTTCCAGAGGTGGTACTAAATTCAGGGAAAAAGATGCCAATGATAGGCTTTGGAACAGGAACAACTCCTCCTCAACAAATTATGCTTGATGCTATTGACATTGGCTACAGACATTTTGATACTGCTGCTCTTTATGGTACTGAAGAACCTCTAGGCCAAGCTGTGTCAAAGGCTTTAGAGCTAGGCCTCGTTAAAAATCGCGATGAACTCTTCATTACTTCCAAGTTATGGTGTACTGATGCTCAACATGACCTTGTTCTCCCAGCTCTCAAAACCACCCTCAAAAATCTGGGATTGGAGTATGTTGATCTCTATTTGATTCACTGGCCAGTAAGGTTGAAACAAGATGCTGAAAGCTTAAAATTTAAAAAGGAGGATATGATTCCCTTTGATATAAAAGGAACATGGGAAGCTATGGAAGAATGTTATAGATTGGGCCTAGCAAAGTCTATTGGTGTCAGTAACTTTGGTGTCAAAAAGCTCTCCATACTCTTAGAAAATGCCGAAATCGCTCCTGCAGTTAATCAGGTGGAGATGAACCCATCATGGCAACAGGGGAAACTAAGAGAATTCTGCAAGCAGAAAGGAATCCATGTGAGTGCATGGTCACCACTAGGAGGATACAAACTAAGTTGGGGTTCGCCTACAGTGATGGAGAATCCAATTCTGCACGAAATTGCAGAGGCTAGAAAGAAGAGTGTAGCCCAGATAGCACTAAGATGGATATACCAGCAAGGGGCAATTCCCATTGTGAAAAGCTTCAACAAGGAAAGGATGAAACAAAACATTGAAATATTTGATTGGGAATTGAACCAAGAAGAATTAGACAAAATCAGTCAGATTCATCAAAGCAGATTTCAAAAGGCAGAAATATTTGTATCTGATAATGGACCTTACAAAACCTTGGAAGAGCTGTGGGATGGTGATGTTTGA

>Medtr4g072350 ATGGAAATAAACAAAGTTCCAGAGGTGATACTAAATTCAGGGAAAAATATGCCAATGATAGGTTTAGGGACATCAACAAGTCCCTCTCCACCACATGAAGTCCTCACCTCAATTTTGGTTGATGCCATTAAAATAGGCTACAGGCATTTTGATACTGCTTCTATCTATAATACTGAAGAACCTCTAGGCCAAGCTGTGTCAAAAGCTTTAGAGCTAGGCCTCGTTAAAAATCGCGATGAACTGTTCGTTACTTCCAAGTTATGGTGTACTGATGCTCACCATGACCTTGTTCTCCCAGCTCTCAAATCCACTCTCAAAAATCTGGGATTGGGGTATGTGGATCTCTATTTGATTCACTGGCCAGTAAGGTTGAAACAAGACGTTGAAGGCCATAACTTTAAAGGTGAGGATACAATTCCTTTTGACATAAAAGGAACATGGGAATCTATGGAAGATTGTTATAGATTGGGCATAGCAAAGTCTATTGGTGTCAGCAACTTTGGTATCAAAAAGCTCTCCATGCTCTTAGAAAATGCCGAAATTGCTCCTGCAGTTAACCAGGTGGAAATGAACTCATCATGGCATCAGGGGAAACTTAGAGAATTCTGCAAGCAGAAAGGAATCCATGTGAGTGCATGGTCACCACTAGGAGGCTACAAACTAAGTTGGGGTTCGCCTGCAGTGATGGAGAATTTGATTCTGCGCAAAATTGCTGAGGCTAGAAAGAAGAGTGTAGCCCAGATAGCACTAAGATGGATATACCAGCAAGGGGTAATTCCCATTGTGAAGAGCTTCAACAAGGAGAGGATGAAACAAAACATTGAAATATTTGATTGGGAATTGAACCAAGAAGAATTAGACAAAATTAATCAGATTCCTCAATGCAGACTGCTAAAGGCAGAAATGTTTGTATCTGATAATGGACCTTACAAGTCATTGGAAGAGCTGTGGGATGGCGACCCTTAA

>Medtr4g072360 ATGGAAGCAAAGAAAGTTCCAGAGGTGATACTAAATTCAGGGAAAAAGATGCCAATGATAGGTTTTGGAACATCAGAAAATCCCTCTCCACCACATGAAGTCCTCACCTCAATTTTGGTTGATGCCATTGAAATAGGCTACAGGCATTTTGATACTGCTTCTGTCTATAATACTGAAGAACCTCTAGGCCAAGCTGTGTCAAAAGCTTTAGAGCTAGGCCTCGTTAAAAATCGCGATGAACTGTTCGTTACTTCCAAGTTATGGTGTACTGATGCTCACCATGACCTTGTTCTCCCATCTCTCAAAACTACCATCAAAAAACTGAAGTTGGACTATGTGGATCTCTATTTGATTCACTTTCCAGTGAGGTTGAAACAAGATGTTGAAGGCTATAACATTAAAAGTGAGGATATAATTCCTTTTGATATAAAAGGAACATGGGAAGCTATGGAATATTGTTATAGATTGGGCTTAGCAAAGTCTATTGGTGTTAGCAACTTTGGTATCAAAAAGCTCTCCATGCTCTTCGAAAGTGCCAAAATCTATCCTGCAGTTAATCAGGTGGAAATGAACTCATCATGGCATCAGGGGAAACTCAGAGAATTCTGCAAGCAGAAAGGAATTCATGTGAGTGCATGGTCACCACTAGGAGGATACAAACTAAGTTTTGGTTCGCCTGCAGTGATGGAGAATTCAATTCTGCGAGAAATTGCTGAGGCTAGAAAGAAAAGTGTAGCCCAGATAGCACTAAGATGGATATACCAGCAAGGGGCAATTCCCATTGTGAAAAGCTTCAACAAGGAAAGGATGAAACTAAACACTGAAATATTTGATTGGGAATTGAACCAAGAAGAATTCGACAAAATCAATCAGATTCCGCAATGCAGACTGCAAAAGGTAGAAATGTTTGTATCTGATAGTGGACCTTACAAAACCTTGGAAGAGCTGTGGGACGGTGACGCTTGA

>Medtr4g092750 ATGGAGTTACGTGAACTTGGAAGAACTGGACTTAAACTAAGCTCCGTCGGGTTCGGAGCTTCACCTCTGGGCAACGTCTTCGGTTCCGTTTCAGAAGAACAAGCAAACGCCTCTGTTCGTATCGCTTTTCAATCCGGCATCAACTTCTTCGACACTTCTCCATACTACGGAGGAACACTGTCTGAGAAAGTGCTCGGAAAGGCGTTGAAAGCGTTGAACGTTCCGAGAAGTGAATACATTGTAGCAACTAAGTGCGGAAGGTACAAAGAGGGTTTTGATTTTAGTGCAGAGAGAGTGACCAGAAGTGTAGACGAGAGTTTGGAAAGGTTGCAACTTGATTACGTTGATATTCTTCAGTGTCATGATATTGAATTTGGATCGTTAGATCAGATTGTTAACGAGACAATTCCTGCACTCCAGAAATTGAAGGAAGCAGGGAAGACTCGTTTCATTGGGATTACAGGACTTCCTTTGGAGGTATTTACTTATGTTCTTGATAGGGTTCCGCCTGGAACATTGGATGTGATACTTTCCTATTGCCATCACTCTATCAATGATTCGACTTTGGAGGATATAGTGCCCTATCTGAAGTCCAAGGGTGTTGGCATTATCAGTGCTTCTCCGTTGGCGATGGGTCTGCTCACTGAGGCTGGCCCTCCTGAATGGCATCCAGCTTCACCGGAACTTAAGTCTGCTTGTAGAGCTGCTGCAACTTATTGCAAAGAAAATGGAAAAAACGTTTCAAAGTTAGCAATGCAGTACAGCTTGTTAAATAAAGAAATCACATCGGTGCTTGTTGGCATGAGATCTGTCGAACAGGTGGAGGAAAATGTTGCTGCCGCAAGAGAACTTGCAGCTTCTGGAATCGACGAAGCGGCTCTGTCAGAAGTCAGAACCATTCTAAAGCCTGTCAAAAATCAGTCATGGCCTAGCGGAATCCAGCAAAGTTGA

>Medtr5g097910 ATGGGCAGTGTTGAAATCCCAACAAAGGTTCTTACAAACACATCTAGTCAATTGAAGATGCCAGTGGTTGGAATGGGATCAGCCCCTGACTTCACATGTAAGAAAGACACAAAAGATGCAATCATTGAAGCCATCAAACAAGGTTATAGACACTTTGATACTGCTGCTGCATATGGCTCAGAACAAGCTCTTGGTGAAGCTTTGAAAGAAGCAATTGAACTTGGTCTTGTCACTAGACAAGACCTTTTTGTTACTTCTAAACTTTGGGTTACTGAAAATCATCCTCATCTTGTTATTCCTGCTCTTCAAAAATCTCTCAAGACTCTTCAATTGGACTACTTGGATTTGTATTTGATTCATTGGCCACTTAGCTCTCAGCCTGGAAAGTTTACATTTCCAATTGATGTGGCAGATCTCTTACCATTTGATGTGAAGGGTGTTTGGGAATCCATGGAAGAAGGATTGAAACTTGGACTCACCAAAGCTATTGGAGTTAGTAACTTCTCTGTCAAGAAACTTGAAAATCTTCTCTCTGTTGCCACTATTCTTCCTGCAGTCAATCAAGTGGAGATGAACCTTGCATGGCAACAAAAGAAGCTTAGAGAGTTTTGCAACGCAAACGGAATAGTGTTAACTGCATTTTCACCATTGAGGAAAGGTGCAAGCAGAGGACCAAATGAAGTTATGGAGAATGATATGCTTAAAGAGATTGCAGATGCACATGGAAAGTCTGTTGCACAAATTTCTCTAAGATGGTTATATGAACAAGGAGTCACTTTTGTTCCCAAGAGTTATGATAAGGAGAGAATGAACCAAAATTTGTGTATCTTTGATTGGTCATTGACAAAGGAGGATCATGAGAAGATTGATCAAATTAAGCAAAATCGTTTGATCCCTGGACCAACCAAACCAGGCATCAATGACCTCTATGATGATTAA

>Medtr6g073110 ATGGAAACAAAATCAATTCCAGAAGTTGTGCTTAATTCAGGCCACAAAATGCCAATGCTAGGGTTTGGCACTGGAACAGTACCTTTACCACCACATCATGAACTCATTCCAGCATTCATCAATGCAATAAAAGTTGGCTATAGACATTTTGATACTGCAGCTTATTATGGTTCAGAGGAATCTCTTGGTCAAGCCATAGCACAAGCATTAGAACAAGGTCTTATTAAAAATCGTAGTGACATTTTTGTCACTACTAAACTATGGTGTACTGAGTCACACCCTGGTCTTGTTTTCCCTGCACTGAAAAATTCATTGAAGAGGTTGGGCCTAGAATATGTGGATCTATACCTAATCCATTTTCCAGTGAGGTTGAGACAAGGGGTAAAAGCGATCAACTACACCAATGAAGACATTCTTCCTTTTGATATGAAAGGGACATGGAAAGATATGGAACAATGTGCTAATCTGGGCTTGGCTAAGTCTATTGGTCTTAGCAATTTTGGTGTGAAAAAAATTTCAGAAATTCTAGAATATGCAACTATTCCTCCTGCTCTTGTCCAGGTGGAAATGAATGCAGCATGGCATCAAGAATATCTGAGAAAGTTCTGCAAAGAGAAAGGGATTCATGTGAGTGCATGGTCCCCTTTGGGTGCTAATGGAGCACCGTGGGGTTCACTTGCTGTTATGGACAATCCAATAATAAAAGACATTGCAATCTCATCAGGAAAAGCTATAGCTCAGGTTGCATTAAGATGGCTAATAGAGCAAGGTGCTACTCCAATTGTGAAGAGCTTCAACAAGGAGAGAATGAAAAAAAATATTGAACTATTTGATTGGGAGCTAAGTGAGGTTGATTTGGAGAAGATTAAACAAGTATCACAATGCAGGGGTTTTAAAGGAGAACGTTTTATCACTGAAAATGGACCTTACAAAACTACTGAAGATCTTTTTGACTGA

>Medtr7g021670 ATGGCAACAGTAGGAAGAATGAAGTTAGGATCACAAGGGATGGAAGTATCCTTACAAGGACTTGGTTGCATGAGCATGTCAGCTTTCTATGGTCCTCCTAAGCCTGAATCTGATATGATTTCTCTCATCCACCATGCTATACAATCTGGTGTCACTTTTCTTGACACTTCTGATATTTATGGCCCTCACACCAATGAAGTTCTTCTTGGAAAGGCTTTGAAGGTAGTTAGAGAGAAAGTTGAATTGGCTACAAAATTTGGAGTCAGAGCTGGTGATGGAAAAGTTGAGATCTGTGGTGATCCAGGCTATGTGAGAGTAGCTTGTGAGGGCAGCTTGAAGAGACTTGATATTGATTGTATTGATCTCTATTATCAACATCGTATTGAAAGCGAAGAGAAAGTGGTAACCTTTCCAAGAAGAACTTCATTGGATTTGCCTAGATTTCAACCTGAGAACCTGCAACAGAATCAGACCATTTTTGACAAAGTTAATGAACTGGCCACAAAGAAGGGATGTACTCCATCCCAGCTTGCATTGGCATGGCTTCATCACCAAGGAAACGACGTGTGCCCGATACCTGGAACCACCAAAATTGAGAACTTTAATCAAAACATCGGTGCTCTATCTGTGAAACTAACACAAGAAGAAATGGTAGAACTTGAGTCTTTAGCAGATTCTGTTAAGGGTGGTAGATATGTAGAGGATAAAAGTACATGGAAGTACTCTGATACTCCACCACTCTCTTTTTGTAAAGCTGCACAATGA

>Medtr7g021680 ATGGCAACAGTAGGAAGAATGAAGTTAGGATCACAAGGGATGGAAGTGTCCTTACAAGGACTTGGTTGCATGAGCATGTCTGCTTTCTATGGTCCTCCTAAACCTGAACCTGATATGATTTCTCTCATCCACCATGCTATACAATCTGGTGTCACTTTTCTTGACACTTCTGACATTTATGGCCCTCACACCAATGAAGTTCTTCTTGGAAAGGCTTTGAAGGGTGTGAGAGAGAAGGTTGAATTGGCTACTAAGTTTGGAGTCAGAGCTGGTGATGGAAAATTTGAGATCTGTGGTGATCCAGGTTATGTGAGAGAAGCTTGTGAGGGTAGCTTGAAGAGACTTGATATTGATTGTATTGATCTCTATTATCAACATCGTATTGATACTCGTCTTCCGATTGAAGTCACGATCGGAGAGCTTAAAAAACTTGTTGAAGAGGGAAAAATAAAATACATTGGTTTGTCTGAGGCCTCAGCTGCAACAATCAGAAGAGCACATGCAGTTCATCCAATAACAGCTGTGCAGTTGGAGTGGTCACTATGGTCAAGAGATGTCGAGGAAGACATAATTCCAACTTGCAGGGAACTGGGTATTGGAATAGTTGCATATAGTCCTCTTGGGCGAGGATTCTTTTCAACAGGAACAAAGTTACTCGACAACTTGCCACAGGATGATTACCGGAAGCATTTGCCTCGATTTCAAACTGAAAACCTGCAGCAGAATCAGACTATATTTGACAAAGTTAATGAATTGGCTACAAAGAAGGGATGTACTCCACCTCAGCTTGCACTAGCCTGGCTTCATCACCAAGGAAATGATGTGTGCCCAATACCTGGAACCACCAAAATTGAGAACTTGAATCAAAACATCGGTGCTCTATCTGTGAAACTAACACAAGAAGAAATGGTAGAACTTGAGTCTTTAGCAGATGCTGTTAAGGGTGGTAGATATGGGGATGAAATAAGTACATGGAAGAATTCTGATACTCCACCACTCTCTTCTTGGAAAGCTGTGTAA

>Medtr7g021850 ATGGCAACAGTAGGAAGAATGAAGTTAGGATCACAAGGCATGGAAGTATCCTTACAAGGACTTGGTTGCATGAGCATGTCTGCTTTCTATGGCCCTCCTAAACCTCAAACTGACATGATTGCTCTCATCCACCATGCCATACAATCTGGTGTCACTTTTCTTGATACCTCTGACATATATGGCCCTCACACCAACGAACTCCTTCTTGGAAAGGCTTTGAAAGGAGGAGTTAGAGAGAAGGTTGAATTGGCTACAAAGTTTGGAGCCAAATATACTGAAGGGAAATTTGAGATTTGTGGTGATCCAGCTTATGTGAGAGAAGCTTGTGAAGCTAGCTTGAAGAGACTTGATATTGATTGCATCGATCTCTATTATCAACATCGTATTGATACTCGTCTTCCAATTGAAATCACGATTGGGGAGCTTAAGAAACTCGTTGAAGAGGGAAAAATAAAATACATTGGTTTGTCTGAAGCCTCAGCTTCAACAATCAGAAGAGCACATGCAGTTCATCCAATAACAGCTGTGCAATTGGAGTGGTCGCTATGGTCAAGAGATGTGGAAGAAGAAATAATTCCAACTTGCAGGGAACTTGGTATTGGAATTGTTGCATATAGTCCTCTTGGGAGAGGATTCTTTTCATCAGGAACAAAGATTGTTGAGAACTTTACAAAGGATGATTACCGCCAGTATATGCCGAGATTTCAACCTGAAAACCTGCAGCAAAATCAGACTATATTTGAGAGGGTTAATGAACTGGCTGCTAAGAAGGGATGTACTCCATCTCAGCTTGCATTGGCATGGCTTCATCACCAAGGAAACGACGTGTGCCCGATACCTGGAACCACCAAAATAGAGAACTTTAATCAAAACATTGGTGCTTTATCTGTTAAACTAACACAAGAAGAAATGGCAGAAATTGAATCCTTGGCAGATCTTGTTGAGGGTGACAGAACCGGAAAGGAACCTACATGGAAGGAATCTGACACTCCACCACTTTCGTCTTGGAAAACTGCGTAA

>Medtr7g063580

ATGATCCACCTTTTATGCCGAACCTTTTGCTTTATTTCACATTTATATTTATCTTCAACAATTTTACTATGCAACATTTATAGCAAAATTATCTCAAGAAGGGAGAAGATGGAAAAAGAAAATATACATGGTCCTCAACATTTTGATCTGAATACTGGTGCAAATATACCATCAGTTGGTCTTGGAACATGGAAAGCTTCTCCTGGTGTTGTTGGTGATGCTGTTGTTGCTGCAGTCAAGGCTGGCTACAGACATATCGATTGTGCTCGGGTATATGATAATGAAAAAGAGATAGGCGAGGCGTTGAAGACACTGTTTTCTGCTGGGGTTGTACAGCGTGGTGAAATGTTCATCACATCAAAGCTATGGATTAGTGACTGTGCACCTGAAGATGTCTCGAAGGCACTGGCTAGGACTCTAGAAGACCTGCAGCTTGACTACATCGATTTATATCTGATACACTGGCCGTTTAGGACGAAGTCGGGATCAAGGGGTTGGGACCCTGAGGTCATGGTTCCCTTATGTCTTTCAGAGACATGGAATGCAATGGAAGGTTTATTCGCCTCAGGTCAAGCACGTGCGATTGGTGTCAGCAACTTTTCAACTAAGAAGCTTCAAGACTTACTCGGATATGCTAAGATTCCGCCAGCAGTTAACCAAGTTGAATGCCATCCTGTTTGGCAACAACCAGCTCTTCATAATTTGTGCAATTCTACTGGTGTTCATCTCACGGCATATTCTCCTCTCGGTTCTCCAGGATCATGGGTTAAGGGAGAAATCTTGAAGGAACCAATTTTGATTGAAATTGCTGAAAAACTTAACAAGTCTCCGGCACAAGTGGCTCTAAGGTGGGGTATCCAAAGTGGTCACAGTGTCCTTCCAAAGAGTGTAAATGAATCTAGGATCAAAGAGAATCTTAGCTTATTTGATTGGTGTATCCCTCCAGAACTCTTCTCAAAATTCTCACAGATTCACCAGCAAAGGCTACTTAGAGGGGACTTTGCAGTCCATGAAAGTTGTAGTCCATACAAAAGTCTTGAAGATTTGTGGGATGGAGAAATATGA

>Medtr7g070500 ATGGCACAAACAGTTAAGCCACATGAACCAAAGACAAAGTCATTTGATCTGTTGAGTGGACATAGCATTCCTGCTATTGGATTAGGCACATGGAAATCTGGTTCACAAGCTATCAATTCTGTCTTCACAGCCATTACTGAGGCTGGATATAGACATATTGACACTGCTGCTCAGTATGGAGTTCAAGAAGAGGTTGGACATGCACTTCAATCTGCCATGCAAGCAGGAGTGGAAAGGAAGGATCTATTCATCACCTCCAAGATATGGTGCACTGACTTGACCCCTGAAAGGGTAAGACCTGCCCTAAACAACACCCTTCAAGAACTCCAACTTGACTACCTTGATCTTTACTTGGTTCACTGGCCATTTCTATTGAAAGATGGGGCAAGCAGGCCTCCTAAAGCAGGAGAAGTGTCGGAGTTCGACATGGAAGGAGTTTGGAGAGAAATGGAGAAGCTTGTCAAGGAAAATCTTGTTAGAGACATTGGAATATGCAACTTCACTCTTACTAAACTGGATAAGCTAGTCAATATTGCTCAAGTTATGCCTTCTGTATGCCAGATGGAGATGCATCCTGGGTGGAGAAATGATAAGATGCTCGAGGCTTGCAAGAAGAATGGCATCCATGTCACGGCCTATTCACCACTTGGATCACAAGATGGTGGGAGAGATCTCATCCATGATCAAACGGTTGATAGGATAGCCAAGAAGCTGAACAAGAGTCCAGGGCAAGTGTTGGTGAAGTGGGCCATGCAGAGAGGGACAAGTGTCATTCCCAAATCAACCAACCCAAATAGGATCAAAGAGAATGTGGTTGTCTTCAATTGGGAACTTCCAGATAATGACTTCAACAAACTTAGCAAAATACCAGATCAGAGGAGAGTCCTCGACGGTGAAGACCTCTTTGTGAACAAGAGTGAAGGGCCATTCAAGAGTGTAGAAGATATCTGGGACCATGAAGATTAG

>Medtr7g114970

ATGGGAGATACTATTCATATTCCTCGAGTGAAGCTTGGAAGCCAAGGCCTAGAAGTTTCTAAGCTTGGATATGGATGTATGGGCCTCACTGGAGTATACAACGCTGCTGTTCCAGAAGATGTTGCCATATCTTTGATCAAACATGCTTTCTCCAAAGGAATCACTTTCTTTGACACTGCTGATTTTTATGCTGCACATACCAATGAAGTTTTTGTCGGAAAGGCACTTAAGGACATACCACGAGATCAAATTCAGATTGCTACAAAGTTTGGGATTGTCAAAATGGAATCTGGTAACGTTGTAGTAAATGGTAGTCCTGAATATGTTCGATCATGTTGTGAGGGTAGTCTTCAACGTCTTGGGGTGGATTACATTGATCTCTATTATCAGCACCGTATTGACACCACTGTTCCCATTGAGGACACTATGGGAGAGCTTAAGAAGTTGGTTGAAGAGGGAAAGATTAAGTACATAGGATTATCTGAGGCTAGTACTGATACAATCAGAAGGGCACATGCTGTTCATCCCATTACTGCTGTTCAAATGGAATGGTCTCTTTGGACTCGTGAAATTGAGCCAGATATCATTCCCCTTTGCAGGGAACTTGGCATTGGAATAGTACCGTACAGTCCCCTTGGCCGTGGATTTTTTGGAGGCAAGGCTATTACAGAAAGTGTACCTGCAGACAGTTTTCTGGCAATCCAACCAAGGTTACAAGGGGAAAACTTTGACAAGAACAAGATCTTTTATCATCGGATGGAAAAGTTGGCACAAGAGAAGCATGAATGTACATCTTCACAACTTGCTCTTGCATGGATTCTTCATCAAGGAGACGATGTAGTGCCCATCCCTGGAACAACTAAGATAAAAAATCTTGAAAGTAATATCAGTTCGTTTAAAGTGAAACTCAACAAAAATGATTTGAAGGAGATTGAAGATGCTGTGCCAATATCTGAGGTGTCAGGGGATCGGACAACTGGTGCTTTTGTTCAATGTTCTTGGAAGTTTGCTAATACTCCAACAAAATCATAG

>Medtr7g114980 ATGGCCACAACACAAACTGAATTAATTCCTCCTCCTCAGGTCCCACTTGGAACCCAAGGCTTTCAGGTTTCAAAATTGGGTTTCGGGTGTATGGGACTCACTGGAGCTTACAATGATCCTCTTCCTGAACAAGATGGTATTTCCGTAATTAACTATGCTTTCAGTAAAGGCGTTACTTTTTTTGATACTGCTGATATCTATGGAGGTAGTGGTGCTAATGAAATTTTACTTGGAAAGGCATTAAAGCAATTGCCCAGAGAAAAGATCCAGTTGGCTACAAAATTTGGTATATCTAGAAGAGACGTTTCTCGTCTCGCTGATGTGACCATCAAGGGTTCACCTGAGTACGTGCGCTCGTGTTGTGAAGCTAGCTTGAAACGTCTCGATGTTGAATACATTGATCTCTATTATCAGCACAGAATTGATACTTCTGTGTCTATTGAGGATACAGTAGGTGAACTTAAGAAACTGGTGGAAGAGGGAAAAGTTAAGTATATTGGACTATCTGAAGCCAGCCCTGATACAATAAGGAGAGCGCATGCTGTTCATCCCATCACTGCTGTACAAATAGAGTGGTCTCTTTGGACTCGCGACATTGAGGAGGAGATAGTTCCTCTCTGCAGAGAGCTTGGTATTGGAATTGTACCATATAGTCCTCTTGGTCGTGGTTTCTTTGGTGGCAAAGGGGTTACGGAAAATGTGCCTGCTGTTAGCTCCCTGACTTCTCATCCCCGCTTCCAAGCTGAGAACTTGAACAAGAACAAAAACATATATGATAAGATCGAAAGTCTTGGCAAAAAGCATGGGTGCACTCCTGCTCAGTTAGCATTAGCATGGGTACTCCAACAAGGAAAGGATGTTGTGCCTATTCCCGGAACAACGAAGATTAAGAATCTGGATCAAAACCTTGGTGCCTTAGCAGTGAAACTATCAGAAGAGGACCTGAGAGAAATTTCTGCAGCGGTTCCTGTGGATGATATAGCAGGTAGTAGATACTACAATGGATTTGATCATATTTCCTGGAAGTTTGCTAACACACCTCCAAAAGTTTGA

>Medtr7g114990 ATGGCCGCAACACAAACACAAACACAAACTGAACTAATTCCTCATGTCTCACTTGGAACCCAAGGCTTTCAGGTTTCAAAGTTCGGTTTAGGGTGTATGGCCCTCAGTGGAGGATACAATGATCCTCTTCCTGAAGAAATTGGCATTTCCGTAATTAATCATGCATTCAGTAAAGGCATCACTTTTTTTGACACCGCTGATGTTTATGGACTCGATGGTGGGAATGAAATTTTGGTTGGAAAGGCTTTAAAGCAACTGCCCAGAGAAAAGATCCAGGTGGCTACAAAATTTGGTATATCCAGAAGTGGCGGTGGTATGGGCATCAAAGGTTCACCGGAGTATGTGCGTTCAAGCTGCGAAGCTAGCTTGAAACGTCTCAATGTTGAATACATTGATCTCTATTATCAGCACAGAGTCGATACAACCGTGCCTATTGAGGATACAGTAGGTGAACTTAAGAAACTGGTGGAAGAGGGAAAAGTTAAATATATTGGACTATCTGAAGCCAGCCCCGATACAATAAGGAGAGCACATGCTGTTCATCCTATCACAGCTTTACAAATAGAGTGGTCTCTCTGGACTCGCGACATTGAGAACGAGATAGTTCCTCTCTGCAGAGAGCTTGGTATTGGAATTGTACCATATAGTCCACTTGGTAAAGGTTTTTTTGCTGGCAAAGGAGTTATCGAAGATGTGCCGAGCTTCATGACTTCTTTTCCCCGCTTCCAAGCTGAGAACTTGGTCAAGAACAAGGTTATATATGATAGGATTGAAAGTCTTAGCAAGAGGCATGGATGCACTACTGCTCAATTAGCATTAGCATGGGTACTCCAACAAGGCAAGGATGTTGTGCCTATTCCTGGAACAACCAAGATTGAGAATCTGGATCAAAACCTTGGTGCATTAGCAGTGAAACTATCAGAAGAGGACATGCGGGAAATTTCTGCTGCAGTTCCTGAAGATGATATAGCAGGTAGTAGATACTACAATGGAATGGATAGTTTATCCTGGAAGTTTGCTAACACACCTCCAAAAGTTTCAACGGTCTCAACATGA

>Medtr7g115010 ATGGCAACAATGCAAACCGAGCTAATTCCTCATGTTACACTTGGAACCCAAGGCTTTCAGGTTTCAAAAATGGGATTTGGGTGTATGGGACTCGGTGGAGCCTACAGTGATCTTCTTCCTGAGCAAGATGGTATTTCCATAATTAAGTATGCATTTAGTAAAGGCATCACTTTATTTGATACTGCTGATGTTTATGGAGTCGATGGTGGTAATGAAATATTGGTTGGAAAGGCTTTAAAGCAACTACCTAGAGAAAAGGTCCAGGTGGCTACAAAATTCGGTATAGCCAGAAGCGATGATTCTGCTAGCTTGAAACGTCTCGATGTCGAATACATTGATCTGTATTATCAGCACAGAGTGGATACATCTGTGCCTATAGAGGATACGGTAGGTGAACTTATGAAGTTGGTGGAAGAAGGAAAAGTGAAGTATATAGGGCTATCTGAAGCTAGCCCAGATACAATAAGGAGAGCGCACACCATTCATCCCATCACAGCTGTACAAATGGAGTGGTCTCTTTGGACTCGTGACATTGAGGATGAGATAGTTCCACTATGCCGAGAGCTTGGTATTGGAATTGTAACATATAGTCCTCTTGGTCGTGGTTTCTTTGGTGGCAAAGGAGTTACGGAAAATGTTTCAGCAGTTAGCTCCCTGGCTACTCATCCTCGCTTCCAAGCCGAGAACTTGGACAAGAACAAAAGTTTATATGATCGAATTGAAAGTCTTGCCAAGAAACATGAGTGCGCTTCTGCTCAGTTAGCATTAGCATGGATACTCCAACAAGGCCATGATGTTGTGCCTATTCCTGGAACAACTAAGATTAAGAATCTTGATCAAAACCTTGGTGCCTTAGCCGTGAAACTATCAGAAGAGGACATGAGAGAAATTTCTACAGTGTTTTCCATTGATGATATAGCTGGTGGTAGACACTATGATGGATTGGATCAATCATCCTGGACCTGGCAATCTGCTAACACACCTCCAAAAGTTTAA

>Medtr8g070095

ATGTCCAAGGCAATTCAATTCTTTGATCTCAACACCGGAGCCAAGATCCCTTCCGTTGGCTTGGGAACTTGGCAAGCCGAGGACGATCCTGGCCTCGTTGCTGAAGCCGTCGCTACTGCCATCAAGGCCGGTTACCGTCACATTGATTGTGCTCAATTATATGGCAATCAGAAGGAGATTGGCTTGGTGTTGAAGAAGTTGTTCGATGAGGGCGTAGTGAAGCGCGAAGATTTGTGGATTACCTCTAAACTCTGGAATACTGATCATGCTCCAGAAGATGTACCACTAGCATTGGAAAAGACTTTGGCAGAATTCCAGCTTGATTATGTTGATTTGTATCTTATCCACTGGCCAGTTGCGATAAAGAAAGGACCAATAGGCGCCGAATTCACGGCTAAGGCTGAAGATCTTTTGATACCTAACTTAGCCGGCACATGGAAGGCAATGGAAGCATTGTATGATTCTGGCAAGGCAAGGGCTATAGGTGTAAGCAATTTCTCTATCAAGAAGTTGGGAGATTTGCTTGAGGTTGCTCGTGTTCCACCTGCTGTCAATCAAGTGGAATGCCATCCTTCCTGGAGGCAGGACAAACTCCGCGATTTCTGCAATTCCAAAGGTGTTCACTTCTCTGGTTATTCACCTTTGGGCTCCCCAACCTGGCTTCACACTGATGTCCTTAAGCATCCAATTCTAAATGAGGTTGCTGAGAAACTAGGCAAGACTCCTGCTCAGGTAGCTCTTCGTTGGGGATTACAAATGGGTCATAGTGTGCTTCCCAAGAGTGCAAATGAAAAAAGGATAAAAGAAAACTTTGATGTTTTTGATTGGTCTATACCTGAACACTTGTTTGCTAAATTTGCTGAGATTGAACAGGCAAGATTACTGAGGGGTGACATATTTGTTCATGAAACCTATGGTGCCTACAGATCTGTTGAAGAACTTTGGGATGGTGAAATCTAA

>Medtr8g070115 ATGTCAAACGAGATTCGATTCTTCAGTCTCAACACCGGAGCCAAGATCCCTTCCGTCGGCTTGGGTACTTGGCAGTCTGATCCTGGCCTCGTCGCTCAAGCCGTTGCCGCCGCGATCAAGGCTGGTTACCGTCACATTGATTGTGCTCAAGTCTATGGCAATGAGAAGGAGATTGGCTCTATTTTGAAGAAATTGTTTGCCGAAGGCGTAGTGAAACGTGAAGATTTGTGGATTACCTCTAAACTCTGGAATACTGATCATGCTCCAGAAGATGTGCCGCTAGCATTGGACAGGACTTTGACAGACTTACAGCTTGATTATGTTGATTTGTATCTTATCCACTGGCCTGCTCCGATGAAGAAAGGATCAGTAGGCTTCAAAGCTGAAAATCTAGTGCAACCTAACTTAGCCAGCACATGGAAGGCAATGGAAGCACTCTATGACTCAGGCAAGGCACGGGCTATAGGTGTAAGCAATTTCTCTTCCAAGAAGTTGGGGGATTTGCTCGAGGTTGCCCGTGTTCCTCCTGCTGTCAATCAAGTAGAATGCCATCCTTCCTGGAGGCAGGACAAGCTGCGTGATTTCTGTAATTCCAAAGGTGTCCACCTCTCTGGATATTCACCTTTGGGTTCCCCTGGAACAACCTGGCTTCAAAGTGATGTCATTAAGCATCCAGTTCTTAACATGATTGCTGGGAAACTAGGCAAGACTCCTGCTCAGGTATCTCTTCGCTGGGGATTGCAAATGGGTCATAGTGTACTTCCCAAGAGTACAAATGAAGCAAGGATAAATGAAAATTTTGATGTTTTTGATTGGGCTATACCTGAAGACTTGTTTGCTAAATTTTCTGAGATTCAACAGGCAAGATTACTCCGGGGTGCCTCATTTGTTCATGAAACTTATGGTGGCTACAGATCTGTTGAAGAACTTTGGGATGGTGAAATCTAA

>Medtr8g088160 ATGTTGGTTACGTGGCTAAACCTAATTCGTAGATATTTTAACCATAGCCTCTCATTCATTCATCTCTCTCAAAGAAGTATAGAGAAAGTTGAATTGATGATGTCAGGTGGAGGAGGAGTTCCTGTCTTCAACCTTGCTCCTAATCTCAATGTTTCAAGGCTGTGTTTGGGAACCATGACATTTGGGGAACAGAACACGTTGTCTCAGTCATTTCAGCTTCTCGATGAAGCCTACCATGCTGGAATCAACTTCTTTGACTCCGCAGAAATGTATCCAGTGCCTCAACGTGCTCAGACTTGGGGAATGAGTGAGGAGTATTTTGGCCATTGGATTAAACACCGGAATATCCCCAGGGACCGTCTCGTTATTGCAACTAAGGTTGCTGGACCATCTGGGCAGATGACTTGGATTAGAGGTGGTCCTAAATCTTTGGATGCCACCAATATTAGTCAAGCTATAGATAATAGTTTGTTGCGGATGCAATTGGATTATATAGATCTTTATCAAATTCATTGGCCTGATCGGTATGTTCCAATGTTTGGAGAAACCGAATATGATCCAGTCCAACAATACTCTTCAATTAGTATAGATGAACAACTTGACGCTCTTAGCAGAGCAGTGAATGACGGGAAGATCAGATACATTGGTCTTAGTAATGAAACACCGTATGGCTTGATGAAGTTTATTCAGGTTGCAGAAAAAAGTTCTTCCTACCCAAAGATAGTTTCTTTGCAGAATTCATATAGCTTGCTATGTAGGACTTTTGATTCTGCAATGGCTGAGTGCTGTCATCAGGAAAGTATTAGCCTGTTGGCCTACAGTCCTCTAGCAATGGGTATTCTTTCAGGCAAATATTTTTCCCATGGTAATGGTCCAGCAGATGCTCGTTTAAATCTTTTCAAAGGAAAGTATTCAGAAGGAGAATCCAGATACAACTTGTCCAATAAAGCTATACAAGCAGCTGCTAGGCTTATTGGTATGTAA

>Medtr8g088170 ATGGGAATGCTATTGTTACACCAAGATTGGTTAGTTTTCCCATTTTTCTTCTTCTTCCTTGCAATTTACCTAATCGGTTACTCCATTATCTTCCGTAACAAAAACCCCAAAATCCGATCCGAATTCTCCAGCTGTTTAATCTCCCTCTTTCACGGCACACCCGCCGCAATCTTCGGCGCCATCTCCATCTTCTCCGACCCCAACAGCGGCTTCGCATCCCTCAACACCGCTTTCCAGAAAACCGTTCTTGATTACAGCATCGCTTACTTCGTAACCGATCTATTACACTACGTCGTTTTCTTCCCAAGCGACGTTCTCTTCATCGCTCATCATTTAGCCACGCTTTTCGTTATCGTCACGTGTCGTCACGTCGTTTCTCATGGCTCTTTCTCCGTCGTCGTTTTGCTTGTTCTCGCTGAAGTCACCAGCGCGTGTCAGAATACATGGACGCTCACCGGTGCTTGCCGGAAGGAAAATCGCTTCGCCGCTAGGGTTTACGATGTTCTGTCCCCGCCGTTTTACGTGGTGTATTCTATTGTGAGGGGCTTTGTGGGCCCATACTTTGTGTTTAAAATGGTGGTTTTCTATGCCAGTGGGCTTGCGTATGGGCTTGTTCCTACGTGGATTTGGGTTTCTTGGGCTGTCGTGGTTTTTTCAGCTATTGGTGTTAGTATTTTGTGGGTATACACTCGTTGGGTTGAACTCATTAGTGAAAGAAGAACTGGTGAATACCTTGATATTGCAAAAACATATGGTCTTCATCCTGTATCACTTGCTATAGCTTTTGTTTTGCAACACCCTCTTGTTGCTAGTGTTGTTTTTGGGGCTACCAAATCATGGCAGCTCCGGGAAGTTATAAATGCATGCAAGATCAAGCTTACATCTGAAGTTATTGAAGAAATTAACAAGGTTCATTCAAGGTTTCCAAATCCATGTCCTTGA

MtAKRS' gDNA sequences:

>Medtr0374s0050 CATATTATTGTGTATGTGACTCTCTTTGCTTCCATGTCCCATCATTTTCTTGCTTTTATATAAAGAGAGTTAGATGCAAAGAAAATCAAACAGAGTAGTGTGATACTATCTTCATCATCATCATCATCATCATGTCTGCTTCAAAAATCCCTCAAGTAGTGCTGAAATCTTCATCCAACCAATCCAACATGCCAGTGATAGCATTTGGTACAGCAGCTGTAACAAACAACGATGGAGAAATCACAAAAGTAGCAGTAATTGAAGCTATCAAATCAGGTTACAGACACTTTGATACTGCATCAATATATGGCTCAGAAGAGGCTTTAGGAGAAGCTATAGAAGAAGCTCTTCAACTTGGTCTAATTGGATCAAGAGATGAACTCTTTATCACCTCTAAACTTTGGGTTACTGATAATTTTCCTCATCTTGTTCTTCCTGCTCTTCAGAAATCACTCCAGTAAGCATAATTCTTACCTTTTTCAACTTAGAATTATTAGTCATATATATATATAAAGTTTTGTCTTGGTAAGACTAAATATTTGAATACAACTTATTGAGTTTTGTAATTAATTTTTTTTTACATCATAAAGTTGGTCTATAATTTATTTACCATTCTAATCCAAAAATCATACAAATAATTCTTCTTCTTTGATTAATTTTTCTTTTGAAATATTCTTCTTTGATTAATATCAAAAATAGTTTTTTAAATTCTAGTTTGTCTCCAAAAATATGATTTTATTTTTAGTTTTATTTTAAATTAAAATTGAGGAACCGGAAATTATCATTATTTAAAGTGGCAGTGATAAGTAACTTTTATAACCGTTTCTATGAGCTTTGAACTCCGTCCTTTGAAGTTATAAGGTTAAACATATTATTGAGCCAAGACATCAACCTATGAAACCCAGAGACCTAGTGCGGGTACGAGTATGATATGATACGGATATGAAGATATCCAAAACTTTTAAGATAGGATACAACTAAGATACGTTATCAAAATCTTTAAAATAAAATTAATTTGCATGTAAAATACTTAAAAAACATATGCATTGATACTTTTTATTAGATATAATTATAAAGATCACAATTTACAAAAAATAATACTTGTGGTCTAAAAAAAATGTAGATATAAAATAATACTTTTCTTTCTTATGTTTCGATATCAACAAAAACATCATAAAAAAACTCAATAATTTTTATCTAATGTTGACTGATTGGGTCTCCTCTCTTATGGACGATTTTTATTTATATTGACGAGCTACTTGAAAATATAAATTGTTATAAATCATAGGATCAAAGAGAGGAAGATTCATCTCATAGAAAAGTTAAGTTTAAGAACTTGATAGAGGGGAAAATCTTTTGAATTCATATTGATTGATTAAATGTTGCAGATGAATACATGAGATGCTCTATAAATAGACCTACTAGAGAATCAACAAACTAACTACCTGCTAACAAACTCTAACCGAGTAAAGAAAAAACCTCTAACAAACTTTTATATATTTATTATAAATTACCGTTTTCAAAATCCAGTGATCATCTTCAATGATTACACTCGATCTTTAGTTTGAAACATTGTCATCAATATTAACTAAATAGTATGATAACATTAACCAAGAGGAAGCAACATCGGATTATGGATAACAAACGATGCTAAAATGAAGCGATTACGTTATACTATCCAGTAACAATCAGATTACCAAAATTGTTACTAATATATTAATAATATGAAAGTAGTTCTAAATTTGTTTGTTATGTTAGGACTAATAATAGGAAATTTAGTTTCATGTGCTTGTATATAAATAAATCTGTAGAGACTTTAAAAGGTATCAAGAAATAATAAAAATTTAGTGAGTAAAATTTGGAGTTTGGCACTGACTCGAAAAGTGCACTTTACCTTATCATAGTATCAAAACAACATGGAGCATAAGGTCTGAAATTGATGCCATGTTGCAGGACCCTGAAATTAGAATATTTGGACCTTTATTTGATCCACTGGCCAATCAGTGTGAAGCCTGGAAATTGGGAATTGCCGTATGCTGAAGAGTTAATAACCACATTTGATTTAAAAGGAGTGTGGACATCAATGGAAGAATGCCAGAAACTAGGCCTTACAAAATATATAGGAGTCAGTAACTTCACTCGCAAGAAACTCGAAGATCTACTCTCATTTGCTATCATTCCTCCTTCAGTGAATCAAGTAAGCCAATAAGCTCATCTATGTAGTTTAACGTCGGTCTCAAATAGTTTCACAGCACGCGGAAGCAATAGTAGTCACGGTATTTCCATGTAGTATCACTTTGTAATTGTAATTTAACACTGCAACATGCCGCATTCTAATCGGATATTAATGCAACCAAATATAGGTGGAGATGAATCCAGTTTGGCATCAGAAGAAGCTAAAAGAATACTGTGAAGCAAAGGGTATAATCATAACTGCTTTCTCTCCTTTAGGAGCCAAAGGGGCCAGTTGGGGTAGTAATGAAGTTATGGACAGTGAAATACTCAAGCAAATTGCAGAGGAACATGGCAAAAATATTGCTCAGGTTACCATTTGAGTTTTCTATCAGTTAGTTAAATTCAACTTTTCATCTTAAATCTCCAATTGAGTATTTCATGCACAAGAAAAGAATAGTTAATATTTATGTGATATTCTGAAAATCACCATTGTTGCAAACTATCAATAATTATTTTATTCAAGTCTTACTTGGCTAGGGTTTTGATTATTCAACTATTTGATGAAGTTAATATAATTCTTTGTGGTGTTATAGGTATGTCTTAGATGGTTGTATGAGCAAGGGGTGACTATGGCTGTGAAGAGCTACAACAAGGAGAGAATGAAGCAAAACATGGAAATATTTGATTGGTCACTTGCAAAAGATGATCATGAAAAGATTGATCAAATCAAACAAATTCGTGTCAACAATGGGCCAGTTGTGTTCATTCCTAACCTTTGGGATGGGGAAACTTAAGCCTAATAACTTTAGCAAAATTAAAGACAATTTCCCAATCCGATTTCAAGGTGCTCAAATCTATCAAAATATACTATGCAAAGTAAGCTTAGGGGTGCAAGAATCTGTCAAAATATATGTTACTCACTTGTTTATGTAGAAATACAAAATGGGCTAGAACGAAACAAAAAATCGAACTACGTACAAAA

>Medtr1g042730

AAACTGTCTATAGGCAGCAAACTGAACAACGTGTTTGTATTATGTGGTAGTAAAAGATAAGCTGCTAAGAAGCCATGGCCGGTGTCTGCTCTTTCTTCTCTACCACTTCCTCTGACGTTGTTGCCATTTCACATTCTCTTACACCATCACTGTCTCTCAACAACACTACACTTTCAACAACAAGTAAAAGAAGATGCATTCTGAGGAGGACGAGAACTGTGAGGCCTCTTGTTTGTGCAGCCATCAACAATCCATTACAATACCGGAAACTTGGTGACTCTGACCTTAATATCAGTGAAATCACTCTTGGTACTGTAAGTTTACTTCTTCTCACATTATGTATTTTCCTCTTTTTATTCTTTTTCTTTTTGTTTGTTTACAAATGTTGTTATTTTGTCATCTTTGTTGAGAGTCTCACGTATTGAGTCTAACTTAGTGCATGTTTTGTTCTGCGGTGACAATAATTGATTTTGACTGAATTGATTTTGACTAACTGTGAGTTCAAAGTAAAGTGGTTTGTGTTTAGATGCATTTATGCAAAAATACTTTTTACTATAAATTTGTGTGTGAATATCAATTCTAGAATAAAAAACTTGAAATCCTAGTTTCAACTATAATCAATTCTGGAGGCAAAATTAATTAATTCTCTTAGAAATGGTGGTTGGACCTAACACAACCCCACAATACCAGCTTGTGAGGTGAGGATTGCCCCCACTTATAAACACATTGTCAGGCTATCTCCTATCCGATGTAGGACTCTTAACAAATTCTATACAAAAGCAACCAAACATGTTGGAATCAATTCTACATTGGGCCCTTTAGAAGTAGAACAAAACATAGTATCAACCTTAAAAAAACCCGGCCTGTATAACCCAGGGATTGGCCTAGTGGTGAAGACTTGGACCTAGGAGTATGCTCCTCTTTAGGTGTCGAGTCTGAATCCTCCAGGTACCAACAATTCCTGTGTTGGGCGAGTTCATATAGAGTTTTACTCTGACTTCAATTGGGCCTCGCTAGTGCATGATAGGATTGGTCCTCCGATTAATCAGTTCTTGGATCGTATATCAAATTTTCAAAAAAATAGATGGCTTGTAAGGTGAGAATTGTCCCCACTTATAAACTTATGTTCAGGTCAATCTCATTCAATGTAGGGCTTTAACAATCTTGATTTAAGAAAAAAATTAATGTTATTAAGGGTAAAATTATATTAATGTATTGGTTATTGCTATATTATTATTGTTGTTTTTCTTGTTATTACTATTGATCACTATGATGATTTGAGTATTGTGGCATGTTGTTGATTGATTGGTGCAATGTTTCAGATGACCTTTGGGGAGCAAAACACAGAAAAAGAATCTCATGACATACTCAATTATGCATTTGAGAATGGCATTAATGCTCTTGATACTGCTGAGGCAGTAAGTTCTTGAAATCTAGACTAGATCTGCATTTCCTTCATTCCATCTCATTTTTACACTTGTACAATAACGCTTTATGAAATTCAAGTATTTCGATTGGTTTATCATGTATAAAAAAATTATAATTTTGTGATTAGATGTAATGCTTATTTTGTTTTTAAGTCCAACAAGTTTATTAGTCTGTTTCACAATTCTACTTTTGTTTTGCTAAACCAAGTTATCCCTGCACACAACTTTTGCAATCTACTTCATTTTTATCTTGTGCTGCGACCGTGTGTTTTACATGCCATATTGGATTAACTAGTGAAATTTATGTGTCTGATCAAATTGCTGCTGTTTTGCACTTTGCAGTACCCGATTCCAATGAAGAAAGAGACACAAGGAAAAACTGATCTCTATATAGCTAGCTGGCTGAAATCTCAATCTCGTGACAAGGTATTAATTGTTCAGGAGTTTGGCACATTTTGCTACATATAACGAGCTAACCCTGAAGCTAGATTGAGATTTGGATTTCAGTTATTTTGGAAAGTTTTTCACTGACAGCACACTTTGTTGAGCTAACAGTTTGAAACTATTTGTCTACCCTCTTTGTGCATAGATTATCATAGCAACAAAAGTTTGTGGTTATTCTGAGAGATCGAGTTACTTACGCGACAATGCAGACATTTTGCGGGTTGATGCGGCAAATATCAAAGAAAGTGTGGAGAAAAGTCTTAAGCGTCTTGGGACTGATTATATTGATTTGTTGCAAATTCATTGGTGAGTTTACAGTATGTAAGCATTGGTGGTGTATCTGACTTAAATATGGTTTTAAAACTTTAGCCAAGCATACATTGTTAAAAAAATATGATTTTATTGTTTTAGGTATCTTAAGTTAGAGATGATGAACCAGAAAGCTAAACTAGTTTTTTAACTATTATAGGCCAGATCGCTACGTTGCACTATTTGGTGAATATTCTTATGATCCTTCAAAATGGAGGCCTAGTGTGCCATTTGTTGAACAGTTGCAAGCTTTTCAAGAACTTATCAATGAAGGAAAGGTGACTGATTCATAACATGCTTCATCTTTACTATATTCCTAAAGCAGTTTTGATAAAAAAAAATAAAAAATTGTTTCGACTTAGAGAAGCTGAGCAGTGAGCAGTACTTTTTATTTGTTATATATTTGCTCGAACTGAAATCATGTGATATTTACTTTTAAGTGGTAACCTTCAATCTGTACTAATAACTACATATAAGATCATTTTACAACATTTTGTTATCCATTTTATTATGTTATGTTGCAAATCTTATACGTCCTTGTGATTCATAAGTCTGTGAATAAAGATCAGCTATCATATGCCACACATCAATTAGCATAAACTCACGACCTTCTAGTACATGTGTATGCGATTCAGTTTCTTTGTATTTTTGCATGCATCAGGTACGTTACCTAGGTGTTTCAAATGAGACTTCATATGGAGTGATGGAATTCGTCCATGCTGCTAAAGTTGAGGGACTTCCAAAGATTGTCAGTATCCAAAACAGCTATAGCTTGCTCGTAAGAAGTCGTTTTGAAGGTAAGTACAATTCTGACGTGGATCAGATCTCATTCATTTATCACTTAATTTTGTATTGCTGGATGTAAAGCTGCAGCTCGCTTTGTCCTTATAGTATATGATTTGTGTTGAATCGAAATGCTACTTTTCGATGAAAAATGCAATGCACCATAAGTTTAAAAAGTAATACATTATTGAACCTAGAGAGTCCAAAATAAAAAGGCTTTCTTGTAGATCTTCGAGTACTTGGTCTCTCTTGTTAACGGTTACACTATGCTGTATCAATGAAGACTTGTTAAACAAATGATTACAGATAACATGGTTTAGTTTTTTAGTAAATATGTTGCCAGAGGTCAAATTTCTTTCTCACAAGGGTTCTCATAATGAGCAATTTTTAGGCATACTTGTCTCCTGCTTTTGGGGAAGTTTCATACGCAATTTGGTCCTTAAACTTCAAAAACGTCCAACGTGATCCGTAAACTTTTAGTTTATGGTTTAGTTCTTTTCATTTAATATTCTGTCAAATGTTAACAGGATTGTTTTTTTCTGTAAGGTACATGTTCAGAGTGCAGTTGAACCCTAACTTGGAGGGGGAGGGGGTAAATTCAACTTTTTCCATTAGGAAATTTGCAAAAATATTCGCATGATTTGAGTGTGACGAATTTCTGTCGGTGTTTGATAAAGAGTTTCTGCAATACGCTCTTTACTACAAAGTATTGTCAAATAGCAGCTGTAGTGGCTTTATAGCTCCATAGTGCTGTAGAATTGACCTGACCGCTATTTTCTGTGATTGACTATGTTACTGTTATAAAGATCAAAGTGCACCATGACCCTAAGTTAGAGGGTGTAAAGTGTAATTTTCCCATTTTAAGCTTCTTCAAGTTCTTAGTTAGTTTAATGTTTTTGCAGTTGATCTTGTGGAGGTTTGTCATCCAAAGAATTGTAACATTGGCTTGCTGTCCTATTCCCCACTAGGCGGCGGAACACTCTCAGGAAAATATATAGATATAAATTCTAAAGCTGCAAAAAGTGGAAGATTGAACCTCTTCCCTGGCTACATGGAAAGATATAACCAATCGATTTCACGGGTACATAATACTTCTAGTTCTTGTATTTAAAGTTTTGACCGTTTTTTTCAAAACCGAATTAGACATCGTACCAGCATGACTTCTGGGTCAGGTTAAGTGACTTTAAACCACTCGGACAAAACCACAATTGAACTGCTCAAATAACTGAACCATCCAGTTCAGTTTTTATAACATTGGTTCAGACTATACTCTAAACCACGGTTAACAAGATAACCAATTAATAACTTTCTTCTATTTTTTCATGTTTTTATTTCAGGAAGCAACTATTAAGTATATTGAGTTGGCCGAGAAACATGGTCTAACTCCTGTCCAGCTCGCGCTTGGATTTGTGAGAGATCGTCCATTCATGACGAGTTCAATTATCGGTGCAACTTCTGTGGACCAACTAAAAGAAGACATAGATGCTTTTACAACAACCGAACGACCTTTGCCAGCAGAAGTCATGACAGGAATTGAAGCTATCTTCAAGAGATACAAAGATCCTAGCATCCTTTAAAGACTATACCTTTGAATTTTTCGGGATATTCTTATATTAGTTCCATGGATAGATACATATTGAATCGATACATGAATACTAGGGTTTATAAAACTGTCTGCATTACTTATGAAAACGTTACGCGGCCTTACAAACTTGGTATTGTATAACCTATGTTCAGTTATTGTCAATGATCACATGCATTTATACAAATAACATCACTATTTATTTGATGACAAAACAATATGTGAGAATAGTTGCTTTGACATTGAATAAAAATATATTGTAGTTCCAATTTCTTTC

>Medtr1g047250 TTAATATACAAATACACACACATCTAGTACCTTTAATTAACCACTTCTAGATCCATCAAGTACACACACAAACGTTGTAATCCTCACAAGAACCACCTCTACACTTTTCATAACTCTTTCAACCTCTCATCACCCACTTCATCAAGATGCAATACAACAACTTAGGCCGGTCAGGTCTAAAAGTCAGCCAACTCTCCTACGGAGCATGGGTAAGTTTCGGCAACCAGCTCGACGTCAAAGAGGCAAAATCTCTTCTCCAATGCTGCCGCGACCACGGCGTAAACTTCTTCGACAACGCAGAAGTCTACGCCAATGGTCGCGCAGAGGAGATCATGGGGCAGGCAATCAAAAGCCTTGACTGGAAACGGTCTGACATCGTTGTTTCGACAAAGATCTTCTGGGGAGGACAAGGACCGAATGATAAGGGGCTGTCAAGGAAGCATATTGTGGAAGGGACGAAAGCGTCGTTGAAGAGGCTGGGGATGGAGTATGTGGATGTTGTTTATTGTCATAGGCCGGATGTTTGTACACCGATTGAGGAGACTGTAAGAGCAATGAATTTTGTTATTGATCAAGGTTGGGCTTTTTATTGGGGGACTAGTGAGTGGTCTAGTCAGCAGATTACTGAAGCTTGGGCTGTTGCTAATAGGTTGGATTTGGTTGGACCGGTTGTTGAACAGCCTGAGTATAACCTCTTGAACAGGCACAAGGTAAATTCAAATCCATTTTTGTTGAGATTTGTTGTATATATATATGTTGAATTTGTTTATTATAACCATAATAATGTTGGATTTAGGACCCATTTGGATTGGCTTATTTCTGAGGATCGTAAATAAACTTATTATTCATAAATTTGTCAATATAGTTGATGAAAAAAAAACAGCTTATGTAGATGCAGCCACAAGTTTTGTGAGGTTGTTTAAGAAAACTAATGTTTGTTTTTTTGGCATATTTTAGTACTTTCTCCAATATATATTTGACTCTCTTTGGATAAACAACTTAATTGTAGCTTATAGCACAAGTGCTTATATATAAGCTTGCATAAACTATTTTTACAACAACAGAAAAAATTTATAGCATATACAAAAACAATTTAACTCTATTTAACCTTTTTATATAAGCTTATGCATAAACACTTATCATGATAAGTGCTTGTGCTATAAGCTGCATACATGTTTATAAAAAACATAATTTAGTTTGCTTGTTTTTTGTTGATAAATTTAATCTTCTTGTTTTAGTTTAGTGGTTGGTATGTAATTTTTTTTTAATTTTTGTTGAAAATTGGGTATCCTCCACATGAAACTCAACTGCAGAGACTAATCCCTCAACTCATGTACAACTAAAATGTGTGGCAAATTCTCCATAACAATTTTAACGATGTTATATTGTATGTTTGAGTTTGAGACATGAGACTTTGATTCTATTGACATGTTTAGTGGTTAGTGTGCAACGTATATGACTATTGGTGTGCAATGTATAAGTGAGGAGATTAGATTTCAGATAGAAAAGTGGTTGGTGTGCAATGTATATGACTATTGTAAACCTTTTTTATGCTATTGATAAAAAAAAGTTTTCCAAATTTAAACTGCTGGTGTATGTTTTAAAAAAAATTAAATTTAAAAAGGAACGATTTTTAAGGGTAAAATTGTATAGGAAAAAAGTTCAATCAAAGTTGGTAGTTTTCTTTTGGTTACATCTTATATGTACGAAGTTTCCACCGCCGTTTAGTGTTGATTAATCTTTATCGTGGAACCTGTTGGGCACACATGGTGGGTTCTCCCCTCCCAACCGATGTTTTTGCTTACGCAACCGTTAGAGAACTAACCCTGATCACTTGCTTAAGGGGTCCAAATCCCTTACCACTTGGATAAATCCATTGTTGGTGGTGCAAAGTGGTAGCTATATTTTAGTTTCCGCTATATGATGGTATGGATAATGTTGCGATATCTTATATTTCCCTTTTTGTTTTAACCCTCTGATTATGATGGAAAAGAAGCCCTGATAATTTGGAGTTTGGCTGGCAAGTAAAGTTGGCCAATGATTGTTTCAGGCAAAATTTATCCTTCACTAAATCTCATTTACCGGTTGAGCCAATTGGTTACTGTATGCTTATATGATATGATTGATATAAATGTATCTGTCTTTTAATTTTAATTGGATATTTGTGTTGATTTGATTTGAGTGTGTAAATGTGTGTTGCAGGTTGAGTCTGAGTATCTTCCCCTGTATTCTAGTTATGGAATTGGTCTCACCACTTGGAGTCCACTTGCGTCTGGAGTCCTTACTGGAAAATACAAGAAAGGAGTTATTCCCCCAGACAGCCGTTTTGCTTTGGAAAATTACAAGGTAATAAAAAATTTAAGGCCTTTTATAGTTTTCTTTTTAAATAAATTTCCGTGTCTTTCTTTACTATTTCTTATTTTGGCGTTTTGTAATTTAAAATGAATTGTGTCCCCAAATTTAAATGTAGTTTATGAGAGCATGATAGAAGATGGAATAATTTGGGTTGGGTTTATGAAGAGTTAGGTCAATTTCGGTCTTTTTTGACGACTAGAACAATGAATAAATTCAAAGAGATGAATAACATATAAACTATTTTGGGTCGTTAACTGGGATAGAGATCACAATATGTGAGGGGTACTAATTAGCGTTTATTGAGTTAGTGACCAATTTGAGCTTTTTTTTAGTATGAACAAATTTGAAGTGCTGAAGAAGAATACATAATTAATTTAGCCGAAAATTTAGACATATCAGCACCGTAACTGTAATGATTGATTGCCTTCTAAATTGACATAGGTTCTGACATCATTGTTGTATGATGGATAGAAATGGAATTGTTTTGGCCTCTTTTGTTTAGATTACTGAGCAATTATTTATTTTCCCACTTTTCTTCGTGTGTTGCAGAATCTTGCATCTCGATCACTGGTTGATGATGTGCTCAAAAAGGTTGATGGTCTGAAGCCAATAGCAGATGAACTTGGTGTGCCATTAGCACAACTCTCAATTGCATGGTGTGCTGCTAATCCTAATGTTTCGTCAGTTATCTGTGGCGCTACAAAAGAATCTCAGGTTAGTTTTCCATTATCAAGAATTTTTACAAGGACATAATTTACATATTGAAAGCAACCTTGTGTGTCGATTTTTTACATCATACATTCTTGATACATTATGCATTTAGCAAGAAATACTTGTAGAGAATGTTAAGTTAATTATCTCTAACATTGGCATGAGATAACTCGAACTTTCTGTTACATGTGGCATTAATGTGGAAAGTACTATTCACAATTACCAAGTGTGCGGTATTTGGAAATGTTCATTGTTTTCCTAACGGCATGAAAACTTTTCAAATAGAAGTGAATGAAACTAATAAATACTGAGAATTAGAGGACTTTTTGGTTTTTATTTCTATTTTCTGGTTTCACTGTTTCTTGTATAAATCCTTGACAACAAGAAACAAATTAAAAACAAAGTGACGTTTTCGTTATTAAAAGTAAGAACAAGAAGTTAAAACAAAGAATGGAAACAAAAATTAGACTAGCTCTATATAACGGTACTAACTGCTTGTTAGTAGCTACGAGCAAGTTTTAAAGATGCAAATAGACTGTGAGTTGAAATACTGATATGTAGTCATTGAAACAGTATAATCGAAACTGAAAGTACAATCAATTATGGATGAAGACCTATGAAGCACGGATACAGACACAGACACCGGATACGACACTGACACGCCGACACCGCTAATAATTTGAGAAAATCACATTATTCAGTGTAATCATATGTGTCGTCGTGTCAGTGTCGGACACCGACATGTGTCCGACACCGGGACACGCCTAATCTTAGGAGTGTCCATGTTTCATAGATGGAGACACATTTAAGTAATTTAACAACTATTAAAAAGGTTGAAAATTTGCAGCAAAACAGAAGACAGAGGACATGCACTTGAGTACTAGAGAGAGAAAGGGTGTTAAACTTAACTGTTCCTACACCCAGTGATTCCTTCTGCCGTCCTTATATCACATACTCTTCCGTATTCTTTTGTGGTTCTTGAGTTCCTGGATTGCAAGTAACCTGTTCCAGTTCGCCACTTGATTCTGTTAGGCCACTTGGTTCAATCTTCAAAGAAGCATTCTCAATAGCTCAACAGTCGTCTTATTGAGGGGATTGAACCGATACATGTTTGGTTTGAGTGGAAAGTTTGATTTTTCCTTAAGTAAAGTCTCATGTTTGAAGTCAAGATTTGTTAATAAAAAATTAAGCCCCGGGAGAGTTTTTCCCCCTTATATCCGTTACCCGATTCACCTCAGATTATGCCAAGTTTAGACCAAAGAATAAAAAAAATAAAAAATAAAAAATAAAAACTTAGATATACGTGTCCATTTATATGTTAACTATTATCTTAATTTGTTGAATAACTTCCTGCAAGAATGCACATTACCGTTAGAATTTTGTCCACTTATTTGTATGTATGTGTAGTGTCTATGTGTGTGTGTGTGTTATGTTTTCCTATGCTTAAATCTAGGAGAACCTGAAAGCAATTGATGTAATTCTTAATTTGAGACATAACTTGCTTGCAAGAGTGGAAATTGTCATTCTAAATTGTCTTATCATTTCTGTTTTGCTTTGCTTAGATTCATGAGAATATGAAGGCTATTGAAGTCGTTAAACTACTAACTCCTGCTGTGATGGAGAAGATTGAGGCTGTTGTTCAAAGCAAACCAAAGCGCGCTGATTCATACAGATGAATTTAAGGTTTCTCATATGATACAGACATATATTGTTTGTATTGATGCGGTTTCCTCCTTTTATTAAGTATTCCAAATGTATTTAGGAGCAATGTACTCCAAGGATTTAAGACTGGGATTTGGGTTTACATTATCCTCTTGGATAAATATATGCTTATGGCTTTTGGATAGTTTTTTCTTTTAAAATATCAGGGTATTGTTTGGATTTTGATATAGTCCAGATGTTTCTCTTGATCCATGTAGTTATATCTACCATGGACAAGACATGATTAAGAGCATGTTTGGATTTGATTTATGGGAGCTTATCTACTGACATAAATTTTGTGAGTGTTTGTGATAATTTTTAAAACCAGCTAATGACATTTTTCATAAGCTTTTTT

>Medtr1g102750 CTGACTGAGTGGACACACTTCAACAACCCTTTGGCTTAGTTTTATTTCTTCTAATATGTGCTTTTATATTTCAATTTGCATCTTAAGCAAGGCAATAACAGAGTTGAAGAATGGAGATACAAGTCCCAAGAGTCAAACTGGGTACCCAAGGTTTAGAGGTAAATTTTCATCCTCCTTATTGACAACCACTTAACATTTGTTTTTCACAGTTGAATGATTAGTTCAACATTAAGAGGTAGAAATCATTAAACTACTAGGTAATTAGTAACCAGTTCATAGTGGAAATTTGTTCTTTCAAGATTGATATGTAAAGCTATATGGTTCCCCATTCTAATTTAGATGAAACTTATGGATAAAGAGTCCTTCAGATATATATTCATAATAGTGCTTTGTTCTCCAAATTTTGTTTCCATTCCTAATGATTGCAATGATGTTTTTACTTATTTACCATCATGCATCTCCTGTTTTTTCATTTTGTGGAATGCAGGTGTCTAGGTTGGGATTTGGATGTGGAGGATTATCTGGAATTTATAATGCTCCTCTCTCACATGAAGATGGATGTTCAATCATTAAGGAAGTATTCAATAGGGGAGTTACCTTTTTTGATACATCAGATCTCTATGGACACAAACATGATAATGAAATCATGGTTGGTAAGGTAACATATTATCCAATAAACTGATAAATCTTTAAGTTTGTAAGTCTGAGTGTTGAAAATACATAAGCATCTGTATGGTTTACAAATTGTAATAATTTGATTAAACACTCATGCATTCAAAATTGATTCAAAAATAAATAATTATTTATATGTGACAAATGGAATGTAACTTATATGTCTTACTGATATCTTTATAACATGGTATAAATATAGGTTTTGTTTACACTAATTTTTGTGGAAAAACAAATTTATGGATGAATCTTTTCTTCTGTCATAGGCATTAAAAGAACTTCCCCGCGAAAAAGTACAATTGGCTACGAAGTTTGGCGTTACTTTATCTGATGAGATGGTTTTGGGGGTAAAAGGTACCCCTGAATATGTGAGGGAGTGCTGTGAAGCTAGTCTGAAGCGGCTTGATGTCAAGTATATTGATTTATACTATCAACACAGAGTTGACACTTCAATTCCAATTGAAGACACCGTAAGCTTTGAATATTTTTCCTCTATTGATTTATGCATGCAAATCATTTCCATCACGTAAACTCTAGGTGATTAAGTGCTTTAACATTGACCTCTTTATGGAAGATGGAGGAACTCAAACAACTTGTAAATGAAGGAAAGATAAAATATATCGGGTTGTCAGAGGCTAATGCTGATACTATAAAGAGAGCACATGCAGTTCATCCTATTACTGCTTTGGAAATGGAGTATTCCTTATGGTCCCGTGACATTGAAGAAGAAATAATTCCACTCTGCAGGTATATTAGTTGAAATGTTTTCTGCTCATATAGCACTAGTTACAATGATACTCAAGTACGTATATCATAAATTGTTTTTATCGTTACTAAGAAGCTTTGTGTACTTGCAGAGAGCTTGGCATTGGAATTGTAGCATACAGCCCCCTTGGTCGCGGCTTTTTTGCAGGAAAGTCAGTGGTAGAGGCTTTGTCTAGTCAGAGCTTGCTGGTACGCAGCTAGTTATGATAATGCAGTTTTATATATGTTTGCATAAATTTGGAGAATATCTTATTATAATGGTCAAAATAACATCGATGAGGAAAAATGTCATAAATATCACTGAATTAGTTCCATTCCTGACGCGATTTTCTTTTGAATATTTTTCTTATTTCTGTAACAGGCTATGCATCCAAGGTTCACAGGAGAAAATTTGGAAAAGAACAAGCTTTTTTATGAACGAATCAATGACTTGGCTTCAAAGCATGCATGCACTCCTTCTCAATTAGCATTAGCTTGGCTTCTGCATCAGGGAAATGACATAATCCCTATCCCTGGTACTGTATTTTATATTTCAGAAGAATATGATGATTTTTTTAGCTTTCTTTCAGAAGAATATATTTAGGCAATCTATTTTCGGATTTTACTTGGCCATCTTAAAATTTACTAATCGATAAAGGAAAAAAAAGCATGATGATATTAAAACCCTTTTATTAGTACCTACTTTCACATATCCATCATCTAACTTTGTTTGGCATAATGTGCATTTATTCATTGACAGTACCTAAAAACTTGACCATATACTGTGTAGATACCGTAAGCATGAGAATAAATCGATTCTATTGACCAAGTGCTTTATGATATATATTTGTTTGATACCATAGGGACTACTAAACTGAAGAACTTTGAAAACAACATTGGATCTCTGAATGTGAAGCTTACACAAGAAAATTTGAAGGAAATTTCTGATGCAGTTCCCGTTGAGGAAATTGCTGGGGAGCGAGAATATGATAGTATATCACAATATCTTTGGAAGTTTGCAACTACGCCGCCAAAGTAATTGCATAGATAGAACTGTGCAACTGTAATGAATTGAGTGTTGTGTTTGTATTTCACTATGATGTTTACCTCAATGAAATAACTTCTCTACAAGATAATGACAATATGTTACATGGGAGAATTGTTATTTCAAGCCTATCTTCTTGGATTTTCATTAGTAAACGACAAAGCAGTTATGAATTACGGCCTTTTATT

>Medtr2g085125 ATGACTGGGTTATTGCAGCATTCTCTGAGCAAATTCACTTATTACAATTCAAACAGAACACCAACAAGAAGAGGAATTGTAACAAGAGAATATAAGCATTTGAGACCATGTCAGTGTGTGACTACCCAGGAGGATAATCGGCGCATTGTGGTCAGTAATGGGAAAGATTCATTGGATATAACTCGGGTTGTGAATGGAATGTGGCAAACGAGTGGTGGATGGGGGAAAATTGATAGGGATGAGGCTGTTGATTCTATGCTTAAATACGCTGATGCTGGATTAACCACTTTTGATATGGCGGATATATGTATTCAACTTTCCTTGTTGCTTCTACTTGCACCTAATTTGTTTGAGTCCATGTAACCACAATTCCCAAAAAAAATTGGCTTTGTTTGGTGTTGAGAGCTTTGCATTAAATAGTGATGTGACATGAACCGAATTCTTACAGGGCGGTTTTATGAAGTTTTGTCGAGTTGATTTAGGTTCAATCCGAATTCTAAGACTCTGCTTCCATTTCACTTAATTGTATCCATCCATTAGAGTAGTAGTGTTTAGAGACTGTTCTCATTTGAGTAGGCAGAATGTTATCTGATGTTATAGATGTACAATCTTCTTTACATGAGCTGCTGTTTTTTAGTCTGCATAATTAAAGGTCATAATATGTGTAAATTGTTTTCTCATTTGTTGTGCTCTTTTGGTTTTGCAGATGGGCCAGCTGAAGATCTATATGGGATTTTTATCAATCGAGTTCGTCGTGAGCGTCCACCAGAATTTTTGGAACAGGTCAGAGGGTGAGTTGATTTTGAGATTCATATTATAAGTCTTTACTATTAGAAGAAAATGCAGCAAGTTTAGATATGCATTTGGAAAATATATATCCTATTATATAATTCTAGGTTCTCAAGAAAATTACCATAGAACATAGGAAGTACTGTCTCAGCCATTGTATTATTTGATGCAGTCTCACTAAGTGGGTGCCACCGCCAGTTAAGATGACAAGTAGCTTTGTAAGAGACAACATTAATGTGTCAAGGAAGAGAATGGACGTGGAATCCTTGGATATGCTTCAGTTCCATTGGTATTTAAATTCTACCCCTCTTGTTATTTGGTGTATGCTTAATATTTTACAATTCTTCATAACATGGTCATTCATCGACTTTGCTTCCTAGTATGTTACTGAAATAAATGAGTATTTGATGTCAATTATGTAGTTGATTTACTTATTGATGATAATTCTGATTCTCTGAGGTTTAAGTTTCTTGTGACAACTCTCCTTTATCTGTAACAAATTTAGGTGGGATTATTCAAATTCAGGATACCTTGATGCACTAAAACACCTCACAGACTTGAAAGGAGAAGGTTAGTTTTGTTCCTGATTAACTCATCTAAGTTCCCGTTTCTAGATTTAAGCAAACTGTTTCTGTTAACCTTGTACAGGTAAAATCAAAACTATAGCTTTAACAAATTTTGATACCGAGAGATTACAAATTATTCTTGAAAATGAGATTCCCATTGTGAGCAATCAGGTAGACCCAAGTGCTTTAATTTCTCTTCTCTCGGTTTATATAAACTTACTGCTGGTTCATTATTCTTATAAAGTATCTTCACTGTCAGGTACAACATTCACTTGTTGATATGCGTCCCCAGCAGAAAATGGCAGAGCTTTGTCAGCTTACAGGAGTCAAACTGATAACGTCTGTCCCCCATTTAAGAAATTATTTCATCACAAAATGATTCTCCAAAATGAGATTAAAGTGAAATTAAAGAAATTAATTTACCAAAATTTCCCCCCTATTATGATGCAGGTATGGGACAGTAATGGGTGGTCTGTTGTCTGAGAAGTTCCTTGACACCAACATAAACATCCCTTTTGCTGGACCTGCAATAAACACTCCATCCCTCTCAAAATACAAAAGGGTAAGATATCATGACCATCATTAACTATCTTAAGTTAGTTTCAAGCTGATAATTGATCCTTTTCTCGCTTAAGAACACTAGGAGGATGAGATTTCATTTCTTTGTTCTCGTAACGTAGTAATATTATTCACGTCTTTTGCTGTACTTTGCATCACATGGCAGATGGTGGATGCTTGGGGAGGATGGGGTTTGTTCCAAGGACTTCTTCGAACTCTTAAACAAGTATCTACTAAGCATGGTGTTTCCATTGCAACTGTTGCTGTGAAGTATATACTTGACCAGGTGAAGTTGATTTGTTTTCTCTTCAAGAATTTGTAATTCTAGTAAACTTAAAATCTCATATTGATATTAAATTGGACAAGCCCGTTCACACATTTCTTTGATTTTGGTTATGGTGAAACATGCAGCCTGCTGTAGCAGGATCAATGGTTGGTGTAAGACTTGGCTTGTCAGAACATATACAAGACTGCAATGCTATCTTTTCACTTGTTCTTGATGAAGAAGATGTAAACAGCATAAGGGAAGCAGCAGGGAAAGGCAAGGATCTTCTTAAAGTGATTGGTGATTGTGGAGATGAGTACAGGCGTGCATGA

>Medtr3g083130 ACAACATCCAATAATTCAACATTCTTATCATCACATCAACCTAACAGAACAAACAAAATGGCTGCTACACCTACAGTCCCTGAAGTAGTCCTCCCCTCCTCCACCGGACAACGGAAAATGCCGGTGATGGGCCTCGGCACGGCACCCGAAGCAACCAGTAAGGTTACTACAAAAGATGCTGTCCTTGAAGCCATCAAACAAGGTTATAGACATTTTGATGCTGCTGCTGCATATGGTGTGGAAAAATCTGTTGGAGAAGCTATTGCAGAAGCACTTAAACTTGGACTTATTTCATCTAGAGATGAACTCTTTGTTACTTCTAAATTATGGGTTACTGATAATCATCCTGAGCTCATTGTTCCTGCTCTGCAGAAATCTCTAAGGTAATTAAAAAAAAATCTTCAATAATTAGCTATATTTGTAGTTTTATTCATGAATTATGAATATTGATTTGAATGTATACATTTCTTTTTTTTGCAGGACTCTTCAACTAGAAAACTTAGACCTCATTTTGATCCATTGGCCAATTACTACTAAACCAGGTGAAGTTAAATACCCTATTGAAGTATCAGAGATTGTGGAATTTGATATGAAAGGTGTGTGGACATCATTGGAAGAATGTCAAAAACTTGGTCTCACGAAAGCCATTGGAGCCAGCAACTTCTCAATCAAGAAGCTTGAAAAATTGCTATCCTTTGCAACCATCCCTCCTGCTGTGAATCAAGTAATTAATAATTTATAAGATCATATATTGAAATTAATTTAACTATTTTAGTTGATTTCATGTTTGTTGGTATTAATCACTTTACTAATTATTGATTAAATTAATTTGGATCCTAATAGGTGGAAGTCAACCTTGGATGGCAACAAGAGAAACTTAGAGCCTTCTGCAAGGAAAAGGGTATTGTGGTAACTGCCTTCTCACCCCTTAGGAAGGGTGCTAGCAGGGGTGCTAATTTAGTGATGGACAATGATATACTCAAAGAATTGGCTGATGCTCATGGCAAGACTATAGCTCAGGTACATATATAATATGATCTTAGTGTATATATATATATAGGGATTTGCTAGAACACACCCACTTATTTATGTGGGAGTGTTACTAAAAAGTTAATAAATACACCTTAAGGTGTATGTTGCTATAACACACCTTCTAAAAAAACAAGTGGGTGTGTTCTAGCAAATCCTATAGACATTTGTTAGAATACACCCACTTATTTTTTAGAAGGAGTATTTTAGAAAGTGAATAACTAAAAAGTGGTTAAACACACCTTAAGGTGTAGCTTTGCTAAAACACTTCCACATAAAAACTAGTGGGTGTGTTCTAGCAAACCCAATATATATATATGAAGTTGGTCGGTTTCTAGGTTGATATGATTCTGATTGTGAATTGGCCTAGTGGTATTGGCTTGAAACTTGGGAGTATGTCTCTCCTTAAGGTCTGAGGTTCGATTATCTCTGAAACCAATTTGAGTGGGCTAATTTAACTTTTTAAAAAGAAAAACCTCTAATTTGTGATTTTTTTTTTTTTTTTTTTTTTATGAGCAGTAAGAACTAGCATAATTTATTTGTTCATAAAGTTGCATAAATTTAATGTTCCAATACTTTGCAGATTTGTCTTCGATGGCTATATGAGCAAGGATTGACTTTTGTGGTTAAGAGCTATGACAAGGAGAGGATGAACCAAAACTTGCAAATCTTTGATTGGTCATTGACTGAGGATGATTACAAGAAAATTAGTGAAATCCATCAAGAGAGACTCATCAAAGGACCAACCAAGCCTCTTCTTGATGATCTATGGGATGAAGAATGAAGAATCAGTTCAATTTGAAACATTGGATTTTGCTACTTACTCTCAATAAAGCAAGTCTGTCTCATCATAGTGGTTATTATGAGTACACTTTGTTAGGTGCAGTTGCAAGTATTTTATTTTCAAGAGTTTTGATTCTACATGTACGGATTGTCAAAATATCAATTTTAATTGGAGTGAATCTTCCTACTTTTCTTGCATTGAGAAATGATATTTGAATAATCATTTTTTATAATTTTTGTGACAACTTTATCTTTCATACTCACATTACTTTTTTACTTTCTTTTTCTATTGCTTTGATTTTTG

>Medtr3g092140 ATGGCACTCTCATCTTGCTGCAGTTTTGTTGTTTGTTCCTCAAACATTAACAACCCTTTACCATCTTTTACTTTCAAATTCCCTTCCCTATGGCCATCCACCCAAAACCAAAAGCTCAAGATGGGGCCTCTAAGTGTTTCTCCAATGGGTTTTGGAACTTGGGCATGGGGTAATCAGCTTCTTTGGGGGTATCAAGAATCCATGGATGATGACCTACAGCAAGTGTTTAACATGGCTGTTGAGAATGGTGTTAATCTTTTTGATACTGCTGATTCTTATGGTACTGGAAGGTTTAATGGTCAGAGTGAGAAACTTCTTGGGAAGTTCATTCGAGATTTTCGAGGTTTGTTCATCACACTTTTCCTTTAATTTGGTATAATTTAACTGAACTATGCTAGATGCATGTGCCTTTATATCAATGTAAATATGAGTGTGATGGAAGGGGAAGAGATGATGATATGGATTATGGATTGAAAAGGAAAGGAAATGAAGGCGATGTATAGCTCAATTAAGCACTTAAAACATAAGTGTCTATGTATAAGCTATTTCTATAACAAAGGATAAAATAATGTCAACTTGTTTTGATATAAGCTATTAGTTGTTTTCATAAGCTTAAAACAACTCAAGGACATGTCAATCTGTCGTGTTATTTTTCTAAGCTCTACCAAGCAGTTTCGCAAGTTCTTAAGTCAGTATATAAGTTCTAATAAGTCAATCCAAGCAGGCTAATAAGTAAACTTAATAGACATGGTACTAAATTATTTGATTTCCTATTGTTTTCACTAAATTTTGTATGCACTTCCTTAGAGAAAAAAGGGAGCCAGAATGAAATTGTGATTGCAACAAAATTTGCTGCTTATCCATGGCGCCTTACACCAGGGCAGTTTGTCGATGCTTGCAGGTAGACTTTTGTTGTTGATATGTTCCCATTCTGCCTGAATGATATTACTGGACTAATATACTCATTCTTATTATCAAGTTCTTCCTACGCAGAATTCCATTGCAAATATTTGCTTTACTTATTTGCGTATGCCGTTGAACTTGAATGAAATGAATTTTCAGGGCATCACTAGATAGGATGCAGATTGAGCAAATTGGGATAGGACAACTGCATTGGTCAACTGCAAATTATGCTCCTTTTCAAGAATTAGCTCTTTGGGATGGTTTAGTGGCAATGTATGATAAGGTTACGATATTTCATGTTTAATAGTAGGATCACATAAGGCCTTAAGTGAACCATTTTTTAAATACATTCGGGGTGTTAAAGATTATTTTTTTACATGCAATTTTCATATTTTTATATATCAGATGAGTGTAAACAACGAGTTCAACGGTTCTTGCTAGGAAAAAACATTACTTTTGATTTCTTCAATGATCAAAGTTTATGTCTTAATATTTATATTGTCATCCAGGGTTTAGTTCAAGCTGTTGGAGTAAGTAATTATGGACCAAAGCAGCTTTTGAAGATACATGATTACTTGAAAGACCGCGGAGTTCCGTTATGCTCAGCCCAGGTAGAGTGTTATGCGTGATTGTAATGGTTTTACATTCAAATTTTGCTTTTGGAGAAATGCCTTTTAATGTCAGTAGTGTGATAACCATGGTTTAATTGTCAAATTAGACAAAAATATCCTTGAGAAACTACAACAGAAAAGAAAAACGATAAGGCAGAAGAAATGAACTGAGATCGGTTAAATGAACATGAGTTGCCAGAATAACTAAAAGAGAAGTGATTCCTTGAAATTATGCTGTCAGTATGCCACAACCATTCATTTATTGTCATGGCCTTCTGGTATACTTTCATTAATCTTTGTAATAATTTTGACATTTCAGGTGCAATTTTCTTTGCTAAGCATGGGGGAAGATCAACTAGAGATCAAGAGTATATGTGATTCTTTAGGTATTCGCGTAATTGCTTATAGTCCTCTAGGACTAGGAATGCTTACTGGGAAATACTCAGCATCCAAACTTCCAACTGGACCAAGGTGTGTTGACATTTACGCAAATATTTTAGAACGCTATTAAGTGCTTAATATTATAGCTTAGCATCAATAATTTTTTTTCGGTGTACCTTTGTTTTTAATTGCAAATCACAGGGGATTGCTATTTAAGCAAATACTTCCTGGACTGGATCCTTTATTGAGTTCCCTGAGAGATATTGCAAATAAAAGACGCAAAACCATGTCACAGGTAATCTTTTTCCTATTTTTACCTACTACTTTCGTTCTTTTTTATAAGCCCCTTTAGCGCTTTGTGTATAAATATCGCTCAGTTGCTCGGAGGTAGTACTAAAATTCTCATCAATTTGACTGTCAATTCATTTGAGGCAAATAAATATGTTCCAAGCCTGAAGGGCCTGAAGGTGTACTGTGTAGTAGTATATCTTGCATGAGTGCTTTAGGGCATTTTTTTATCAGTAGGAATCGAACTTGGTATTCTGAAGTTTACAAGTTACAACACTCAGACACAGTGCGCACCAACCACTTGCGCTAGACTTAGGTAGTGGTTTAAGGCATCGGACTATACTTATCTTTCTTTTAATCCATTTTATCTAATGTGGAATTAATCACTCACTTTTGAGTTCCAACAATAGTGGTAGTGGATGGGGTCAATGAATAGATAGTGCTATAAATGTTCTCTCCATTTTGTAGATTTTATTTTCCTCGCAATTATTTGACAAGAGCATTTCAACTACCAAAACCTGTAGTTTTCACATTAATCAACCGCATGCTTACAGTTCCATCTACTATGGCAAAGGTAGGCCCTGCAGGCTTGGCCCATCTTCTGGAGAACATAACTTAATCGTATTCTGCTGTCATACTCAGGCTCTATGCACCTTCCTTCGGGCTTCTGATCCAATCTTTTGTACACTACTAGTACTACTCTGTGTCCTTGTTCTGACCTCCAAATGCCAATATTAAAGACTCGGCATAACAGAAAACTAAAACCAAGATCTGATGGTCATAGAAAGCTTGTGAATAATAGATTATTCAATTAAAGGATTATATGATATCATACGTCATTGATATCTGATGTCACAAAGTAACTGTTTTATCTTGTATGTAACAACATCAGAACTACTCCGACTTCATTATCAGGATCATCCACCAAAAAAGACAAACCTTAAATGTTTTAATGTCGTGTTTTCCAACACAGCTTACTTTTTTAAAGGTTTTAAACTTCCCTTTAAAGAACGACCAACCAACCCACATAACTGTCCATGTCCTAGGTGTGTTACAAATGACAACGTCGATCACTTACTCTGACCAACCTTGCCCGATCAAATAAATTCATTGTCTAATGCATCATTGTCATGTCAGGTTGCAATAAATTGGTGCATATGCAAAGGTACAATTCCAATTCCTGGAGTCAAGTCAATAACACAAGCAGAGGAAAATTTGGGTGCTCTGGGTTGGCGTCTCTCCTCAAACGAGCTACTTCAGTTGGAGGATGCAGCACAGCAATCGCCTCGCAGGATGATCCAAAACATTTTTCAAACCAGGTATGCATGTTATCTAGCTTCTCGATTATAGGCCAGATATTTCAAGGTTAATAAAATTTGTAATTATTAGTGTTTCATGAGACTTCTCTTTCATATGCCAGATGATATTAGCGGCACAAGTTGCACACTGAGAATAACAAGGCAAGGCCTCATGGTTTTTATCATCTTCTAACAAGAAAACCAGAGAAGATTGTTGAACAATTTCATCTATACTCGTCCAGAAGAGATCGTTGTTTTTAAAGTATTGCAGTTACGTGCATATCTTCGTTTTTAAAGTATTGCAGTGTGCATATTGTGAAGAGTTGTGTAACTTCAAGAGCACCAATCCTGATCATATACTTGGACAATCATATAGTGTCATTCATATAATTTTTTGTCACGTATTATGAAGATAAATTTATTGACACAAAAAGAAAGTTTAATTTAAAAATATTTTACTTTTATGCTGCTAAAAAAGCTTCTACTGAGAAACAGCCACACCCACTGACGTAGAAAAATAGTAGGAAAACGAAAGATTCCATAATTGGTGCATCTAAAATTAACATCTAAACAAGTGAGAGGAAAAAATTATCCTTCCTTTGATGCCAGTGTAAACTATTAACATAACTTCTACTAACAAGAAACTATTTCCAATTATACTTCGGATGAACTTTTTTAACAGAAATAACCTTATTTCGTATAATCAATAATAAGAGAATGAATACATGTAGAAATATCCTGAAAGATGCAGAGACTTCATGACAACACCAACCAGCAGCAATGATTTCATAATATGGAGCTTCAAGAACCTAGGGATCTTTTCCAATGTAGAGCCTTAAAAATCATATTCTAACTCCTAAATCAAAAATAAAGTAACTGCATTTCCAAATATATAAAACAAGGTTGTCAACATTAACAAAATGATTAGGATACAAAAATATGATTCACCATTCAAACAAACAAGTCCTAATAATGCTCAATAGTTTTTCTTTCATCTGCACAAAGCAACACTACATTCTTAAGAAAGCAAAAACTAAAAAAGTCCAACCTAAACCGATTGAAGACATATGCAAGTAATATCGATTATATAAGGTATTTACAGATTACGTGTTTCATCGCCTTAAATGAGGATATCCTACAATTCCATGTAGTATACACTATTCAAAGTACTGAAAACAAAATATAAAATAATATTGGAATTAATTTGTTCAATGATTGACGTTTATAAAATGAACTAGAATTTCTCAAGACATTTTAAGACTTGATAGAAGTGGGGCTGAAAGCAAAAATTGCCACAAACCTAGAGGCTGACTGAGATTGGCAGACAATGCATCAGATAGAACAACTGGAGTGCTAACTTTATGCAGAAGAATAGATCACAATCACTTGCGCTAGAATTCTTCTGGGTATGAATATCACTAAACTGCGGTAGTACTAAAATTCTCATGGAAGTCTTTTGACTGCCAATTAGTTTGAAGCAACTAAATATGTTCGAAGCCAGTAGGTGTACTATGTAGTAGTATATCTTGCACAGGTGGTTCAAAGCAATTTTATCGGTGGAAATCGAATTTGGTACCTCGAAGTTTACAACATACATACATATATTGTGCGCTAGACCTTCGTGGTGGTTTAAGGCGTTGAATACACTGGTTGCTTCATTTTGCCTATTTCTATCCGATGTAAAACTAACCACTCGCCTTTGAATTCCAATAATACCCATTACTTAATGAGGTCAATGAATAGATAGTGTTATAACTGTTCTCTCCATTTTGTAGAATTTATTTTCCTCACAATCATCTGAAAGGAGCATTCCAGCTACCAAAATCTATAGTTATCACATTAAGCATGCGCATGGTTACAGTTCCATCTACTTTTGCAAAGGTAGGCCATGCAGGCTTGGCCCATCTTCTGGAGAATATAACTTTATCCTATTCTGCTGTCATATTCAGGCTATAAAGCATCTTTCTTCAGAACTATTCAGCTCTTGTCAAAAAAAAAAAAAATCAGAACTATTCCGACTTCATTATCAAGATCACCCACCAAAGAAGACAACCCTTAAATATCAATTCGTTCGCTTTTTAATGCCATGTTTGCCAACACAACTTACTTTTTTAAAGGTTCTAAACTTCCCTTTAAAGAACTACCATCCAACCTGCATAATTGTCCTTGTCCTATGTGCGCTACAAATGTCAATGCCCATCGCTTACTATGACCAACCTTGCCCGATCAAATAAATTCACTGTCTAATGTATCACATTATCATGTAAAGTTGCAATAAATTGGTGCATATGCAAAGGTACAACTCTAATTCCTGGAGTCAAGTCAATAGCAAAGGCAGAGGAAAATTTGGGTGCTCTTGGTTGGCGTCTTTCATCAGACCAGCTGCTTCAGTTGGAGTATGCAGCACAGAAGTCACCTCCCAGGATTAAAGGATTTTCCAAACCTTCAGTTGGAGCTGCTTGGAGACAAAACATTTTCCAAACCATGTGA

>Medtr3g449790 GTACTCTTTCACCACCTACAACTTCTTCATCACTTCAATATTCAATATAAATTCGAACCTATTTTCTAAAAAAATAATAATAATTCAGACCCATCAATTTTCATTCTCAATGGCTCTTCATATAAACAGTGGTTGTTTTACTGTGATGGGTCACTGTAGAGTTCAAAGAGTAAAAGCTGTTGCTTCAGAGGGTTCTGCTAATGTTACAGTTGAAGATAAGTTGAAATTGGGTGGTTCTGATTTGAAGGTGTCAACTATTGGAATTGGAGCTTGGTCTTGGGGTGATACTACTTACTGGAACAATTTTCAATGGAATGGTAACTTTTCTTTTCTCTTTTTGTATTTCTTATAAGTTGCATTGCACATATGAAGTAAAAACAAATCAATTCTGGTCTTTGAATTTTACTGTGTAACTATGAAGCACACGCACGATATGGACACTGACTCACCAACACCACTAATAATTTGATAAAATCATATAATTCAGTGTAATTATAAGTGTCGGTGTTGTGTTGGTGTCAGACATAACACGTGTCTGACACCAGGACACCTCTAATCCGAGGAGTGTTTGTGCTTCATAGTTGTGTAGAAATTTCTACCTTGTGACGGGAGAAGGAAGTTGACAAACATTTTAGGATTTTTGCTTCCTGAGATACCATTTTAGTTGTGTAGCTTTGAAGATAAAAGGCATATTAGGACCATTTATATTTAATCATGGATCATTATTTTTGAATTCATTTTCTAATTTCTACTCAAGGATGATTAGATAACAATCATCATCAATCTTCTTGCCTTATGGTAATTTTAGATAGGAATGAGAAAGCTGCTAGAGATGCATTCAATACAAGTATTGATGGGGGTCTAACCTTTATCGACACAGCTGAAGTTTATGGCTCTGGGGTGAGTACATATCTTATCGAGTTTTCATTGATGAGTGTAAATAAATTCATTAATTTAACACTTTCTCTTTTTTAAGCTCGCTTTCGGAGCTGTAAATTCAGAAACTCTTCTAGGAAGGTAAAGCTTACTCCTCCGTTTCTGAATATAAGCAAAATTGACTTTTTAGGTTCATTCAATTAATGATGTATGTGGTCTATAATATATGGACCACATACATCATTAATTGAATGAACCTAAAAAATCAATTTTGCTTATATTTAAAAACGGAGGGAGTACATTTTTTGGTATAATTTTCATTTGCAGTTATTAATCAAGGCTAGCATTAATGTTTCTATATGTTTGTAATGTGAAGATTTATTAAAGAACGAAAACAAAAGGATCCAAATGTTGAAGTTGAGGTTGCGACCAAGTTTGCTGCACTACCATGGAGATTAGGCCGTGAAAGTGTTCTAAGTGCACTTAAAGATTCCCTTGATCGACTCGAGATGACTTCTGTGGATCTGTATCAGCTTCATTGGTCTGTATTTTTATAAGTCTATTGATTGATGCTGTTTTTATCTAAAAATATTGTTTTTGATGAAGTGACACGCTTGTTTTTCAGGCCTGGAGTATGGGGAAATGAAGGTTGTCCAAATGTACCAATTTAGTTAATTGAACTATTTACTTAATATAGTAAATGGAACTAAATGTTTCGAAACTTATTCATAATAATCATATTAAGAGAATGCTAGTTCCCCAATGAATTGCTTTTCAGGGTATATTGATGGTCTAGGTGATGCTGTTCAAAAAGGACTTGTAAAGGCTGTTGGTGTTTCAAACTATAGTGGTAACTCTCTGTGATTATTATCTGTTGTGTAGCACCGACACATTACATATTCACGTCTATTTTCTCAAACTACTATCGGTGTCGGTGTCATTGTCTTGTCTGGTGTCAATGATTTGTTGGCGGTTCATAGATTATTTGTCTTGGAGTCTTCTAAACTTCAATTTTATGCAAAACTATGACTAACTTGTACCATAATACTTTTTATGGAACTAGTTCTAGTTCTATGATCAGAAGTACCTAACATTCCTTTTTTCTATTTATACAGAAAACCGACTACGCGAAGCATATAAACAGCTCAAGAAGAGAGGTATTCCATTGGCTTCAAACCAAGTAAACTACAGCCTCATTTATAGGGTCCCTGAAGAAAATGGTGTGAAGAAAGCCTGTGATGAACTTGGGATTACATTGATTGCATATTCACCAATAGCTCAAGGTGAAATCCTTTTTACTATTCAAAACCACATATTAAAAGAATAATACTACCTCTATCCCTTTTTATTAGTATATACCTTTAAGGTTAATAAGTGGGGTGTCCGGTGTTCAAACCTCGGATCCTGCATATATTATGCAATGTCCCTGCCAAGTTAGTAGTGTTATTAATGTCATTTGGTATAGCAATATTACTAAATACTCTCTTCGGTCTTTAATATAAGAAAATTTTCAAAAAAAAATCTGGTCCTATTTATAAAAAAAATGTCACAACTTTTAAGGTGTAGTTATTGCTTAAATGACTTTTTTATCCCTATTTAATGTTTCTCTTTTAAATATTAGTGGGTGTGTTTAATGTTTCACAATTTTTATGAGGGTATATTTGTAAAAATATTCAAAAAGTTACATTAATCAATGAATGCATTAGTATTGATGTTTTTTTTATTTTGTTGCTTATAATAAACACCGGAGGGAGTATTATGGTAGTGTTATTAATACCTTAAATTATGACTTTTCATATAAGCACTTAATAGCCGCAAAATTTATGCAAAATCGTTATCTTAAGCAGGTGTTCTTACTGGAAAGTATACTCCACGAAATCCTCCATCTGGGCCTCGAGGTAGAATTTATACTCCAGAATTCTTAACAAAGGTACAACTTTTCTGAAATTTCCGTATCTAATGAATTATACCGATAGTACTTGGTTCCTCAAATGTTTATTTGTTTTGCAGCTTCAACCACTGCTAAAGAAGATCACTGAAATAGGAGAGAAATACAATAAAACTTCTACACAGGTTCATACTTTTCTTTATTTCCATGAAATAAGTTGAAAATTAGAAATATTAGTATAATGTTTGTGTCATCCTGCTTTAACAATATAGTCTGTTTGTGTCATCCTGCCTTGTTTCCGAAGGAGTTTTATGATTAGAAACAGAGATAGTATAGCATAGAATTTGTTCTGTGAGAATCATTGACTGGATTCACACACATTATGTGTGGAGAAGTGGGGAATCCGTTCGAACCCCAGCCCCTGTACAAATTATCCCTGGTAGCTACCAACTGAGCTACGCTTACAGGACATTTACACAGAAATTTTACCAAATACAAAAGCATTAGACACCATGTTATATTACCATGTGCTCATTGGCTTAATTGCACTTTTCCTCTCACTAATATCACTATTGTGCGTCCCCGCATTCAAATTGAGCGAATTAAATTCCAAAGTAAGTATCCCTCTTATTACATTTAAGTCTCACTTATTGTGTTGTCTAAGTAAATATTTATGGTATTTTGATGGGGCCAAAAAGGAAAGAACATCAGCCATTAAAAGTGCATGTGCTGCACTTGAAAACTCATCCCTTGTGAATAGTCGACAATGCCAATGACAACGGACTTGATTGTAACACGATGAATGCTTAGGGATTTAAAACGTGCAATTAAAACCTGGAGAACCCAAAATTACACAATCGTAATTCGTAACACCTGATAGACTAAAAGTGCAATTAAGGTTTTAATTAGAATAGCCTTCACCGCATGTCGGAGTACAAAAGGTTTCTTTTTCAAATGATACTTGTGAGTCATCTCTTAAAAACAAATTGATTCTTTGTTAGGTATCACTGAACTGGCTGGTAGCTCAAGGAAATGTTGTACCAATTCCTGGTGCTAAGACAGCAGAACAAGCTGAAGAATTTAAAGGTGCATTGGGATGGAGATTGACTAATGAAGAGGTAGCTGAGCTACGAAGTTTGGCGTCGAAAATTAAGCCTGTTATTGGCTTTCCTGTTGAAAAACTCTGAATCATGGTTAATCACATTGCTGCAGCATACACCCCCTGCTAAATCATTTGTAAAGTTATAAATATAGTCTAGCGAAAAAGAAATTGCATCCATAGTAAATTGGAAATTGAGAGCAAATGATATCTCATATCTTATTATAGTTACTATAAAGTGCTTATCTACAAGGTCTACTTCCATTTTTGTTCTTTTCATTAAGTG

>Medtr4g021350 ACTCAAGATTCTTACACACTAGGTCAACATCTCACCGTTCAAATCCATATGAGATCCCATCTAGCCAAATCACAACCATTGAATCTTCTTTCTCTTTTTACACACCATTCATTATTTCTTAGATTCTTTTGCCTATAAATAGCTAGCAATAACAGAATACAATAACAGAAAAACAAAGAAGAAAATGGCCACAGCAATCAAATTCTTTCAGTTGAACACTGGTGCTAAAATCCCTTCTGTTGGTTTAGGTACTTGGCAAGCTGAACCCGGTGTTGTTGCCAAAGCTGTCACCACTGCTGTTCAGGTATAAACTTGTTCAGTTCAAAAGTTGATGCATCTGATTCAAAATTTAGACCAAATACATTAACTTTTGTTGACCTAACTTTTCTTATATATAGGACTGAATTACTATTCATATTTTTGTTTGAGTAAGTGGATATTTAGATTGACGGTGAATTTGGCAAAATCACGATGTTTTGTCGAAGTTTTAAAAAATTTCACCATAATTAAAGTGATGCGATTTTGCAAAAGTCAACATGATTGATTTGAGAGGTGTAGAATTGATTTTGATATTTTGTCTATGTTTGTTTGAGAAGAATTGATTTTGACTTTAGGATTTGTAGCTTTGATTCTAAACTTGATATTTACGTTCAATTTATTGTTCTTATACTTAAATTAATCCAAATACAAACTATTTTACATTGAACTCAATTATAAATAAATTTAATTTTAGTGTTCATTCAAAATCAATATTTGTAATGGCAGAACCAAGAACATATTTATGATGGCAGAACCAAGCACATATGATATAGCTTATGTTTGGTTTCACATTTAGAGGAGCCAAAATTGATTCTAGAGTTTTAAAATTGATATAGTGTATATGAAAATTGGAGTTTTTGATTGTAGAATTGATTTTTACACTCATACTTAGTGCCTGTTTGTTTTCCCATTTGCCCACCACTGCATTGTGTTTACTAGAAGCTAGAAAGTGTGGCTTCTACATAAACGTGCATCGGGACGCGATAATAAAGTGCTGTACCGTGTTCCAAACACAACATTATTGTTCAACTCAATTTACCATGAATCAATTACGACTAGAAATGTAGACGTTGGTAACCACTTTTTAGTATTCAATGTGACATTGCCATTGACTATGAAGCTAGAAATGTAGACATCAATTTGTACACCCAAACAGAAATCATTTTTCAATTAACTCAGTTTTAGTCTTAATCAATATTTTAGAATCAATTTGCTCAAAATCAATTTTTGTGGCCTGACCAAACGTCCACAGAAACAGTTTAGTAGTTTTGTTTATTCTTAGGTTTTGGAGGAGTGAACTAATCTTGTAGATGTCTATGTTTTTGTTCAGGTTGGATACAGACATATTGATTGTGCTGAAGCTTATAAGAATCAATCAGAGGTAACCATTTTTGTTTTATTTAGAGTTTTCAGTTTTTGACAATTACTTGATTGTTTTCATTTAACATGCATGCTTATACTCCACCATAATTTTTTGATACAAACATAATTTATTTGATTTTCTTGTTATTGTATTAATTTTGTTTTAATAAAAAGGGGGAAAAGTGTAGAGAAATAGAACATTTCCTTAACTACAAGCAAATTGACCGTTGTTTTCTTGACTTTTATAGATTGGTTCAGCTCTCAAAAAGCTTTTTGAGGATGGTGTGATTAAGCGCGAGGAATTATGGATCACCTCGAAACTATGGTTTGTTGCTTTACTTTTGTGAAATTGAGTTCTGTTTTTAAGAATGTTGGGAAAATGTTTGTTTTAGGTTGTGTTTAACATAAATCCTTTGCTTTGTTTACTGTTTCAGGTGTTCAGATCATCATCCAGAAGATGTGCCAAAAGCATTGGATAAAACTTTAAATGATTTGCAACTTGATTACCTTGACCTCTATCTGGTAGGCATTAACTTTTCTGCATTGTGAATATATGTTGATATTTTGGTGTAAGACTAAACACACTAAATTGTTACCATGTGACCTTGGTGATTCGGGGATTTTTTATACTTCTTGGTTTTAGGTATCCAATGCATAGCATTGACTATGAAGCACATACACGGACACGTTACTGATGCTAATACTTTGACACTGATAATAACTTGAGAAAATTAACCATATGTGTTGGTGTCTGCTCAGTGTTAGATACTGGCACATGTCGGACACCGTGTGTTAGTGCTACTTATTTAGGAGTTGGATAGTGTTTTACGAATGCTTGATAGCTCTGTAGTACTGAACGAAGGCATGTCCGGTGTCCGACCGGTGTTGTGACCAACAATGACACATGTGATTAAATTTTATTCGACTATTACCGGTCTTATCAATGTTGTGTATGGTGTTCATGTTTGTGTCAGTGCATCATAACTTGATAACTTGTGCCAATGAATTATGAAGTTTGTTTGTACTCAATTGAAATTATGATTTTTCTCAACTGAGTAACCTGCATTGGTTTTACACAAGAAATGTGATGCTGAGTTGAATTACGTTTTTCTGAATCTGATTTCGTTTTGTAGATCCACTGGCCAGTGAGCATGAAAAGGGGAACAGGTGAATTTAAGGCTGAAAATCTCGATCGTGCAGACATACCAAGCACATGGAAAGCATTGGAAGCACTATATGACTCTGGCAAGGCTAAAGCTATAGGAGTCAGCAATTTCTCTACAAAGAAACTTCAAGACTTGTTGGATGTAGCAAGAGTGCCTCCAGCTGTTAATCAGGTGGAATTGCACCCTGGATGGCAGCAGGCAAAGTTGCATGCATTTTGTGAATCTAAAGGAATTCATGTATCTGTAAGTTTAGCACATTAATTCACTTAAAAGAGTTGATATTCATTCAAATACATCCATTGAGTTATTGCAAAATCTTGAGGGACCAATAATCTTGTGAGTAATCAGAGATGTGAGGGATAGAGATAATAGGCTTTAGCAAGATTTTATGGCAACCTTTTCTGGCTGAAAACGTAACATTTTGATTGATTAAGGGCCTTTTTGGATTGACTTATTTGAGTTTTCTATTGACATAAGCACTTGTTGTGAGAGTGTTTGGAATATTTTATGGTAACCGTTTATAACATGTCTCTACAAACTAATTTTCAACTCATTTTCATAAGTGATCCGAGACAATTTATGAAAACAACTTATAGTTTACATGAAAACAATTTAACTTTATTTTATCCTTTTTCCTCCAAAAAAAACTTTATTTTATCTTTTGTATAGAAAGTGTTTATACTTAAACACTTAAATGATAGACGTTTATGCTATAAGTGCTCAATTAAGTTGTGTGTCCAAACAGGGTCTAAAAGATAGTGAATGACTTAATATGCTCCTATTTATGAAGAGCTTAATGAACTAACTAGTAGTAACCAACTTTGTAACTGATCATATATTAAATCATATATTCTATGGACTCTTGAGCTAGTGTAAAGTGATTTTTGTATGTATTCAAATTCAAATGATGGTATCTCAAAAGCCATTTTTTTTCTTATCTCCTCAGGGATATTCTCCTCTAGGCTCACCAGGAGTACTCAAAAGTGACATTCTTAAGAATCCAGTTGTCAAAGAAATTGCAGAGAAATTAGGGAAGACACCAGGACAAGTTGCCCTTCGATGGGGTCTACAAGCAGGACACAGCGTGCTACCTAAGAGCACTAATGAGGCTAGGATCAAGGAAAATCTTGACGTATACGATTGGTCGATTCCGGAAGACCTGTTTCCTAAGTTTTCTGAAATTAACCAGGTAAGTTGGTAGTTAGTTTAATCTAGTTTAGCTTCTGTTAGTTTCTACATCTCTAAATATTTATTTTAGGATTTAATTCGTATACACTTTCAGTATGAAATGTTTTACATTGTCAATTAATCATAACCTTCACTTCATTAAAAATATTTGACTTTGTTCTGAACTATTTTTAAAAATCATTCATTTGATTGGCTATGTTTTGTAGACAACGTAAATTTTTTAAAAGACAGTACATACAAATTAAGCTCTTTATTTTATCTGCATAGCACCGTCACTATATCACTATGCAATTGGTGTCATAACAAATGCAAATTTGGCTCTAATTTCCTGATGACATTTGAACTATTTGATGAAAAAAATCAACTGTTCTTTGAGGATATATCCATGGTTTGATATAAATGTGTCTATACCATTGATCATTGTGTTTATTTATCAATGGTTGAGATGATTTTGAATTGCAATTGCCATAGAGGCTGTGACAGGAGCCTTGATATTGCGGAAAAATACTGCTGGCAAATGCAGTTTAGAACTATGATTGAGATTTCTTACTTTATTATTATCTAAAGTCAATTATTTACTTCAGAGTGTAAAACCAATAAGTAAGGTTCATTATCTGAATTAACACATTGAAACTTACACATGCAGGATAAGCTAATCAAGGGTACCTTTTTCGTTAACGACACCTATGGTGCTTTTAGGACCATCGAAGAACTCTGGGATGGTGAAGTATGAGCAATATGCAACATTGAGATGACAAATAATACACTGTTTCTACAATAAAACCAGATACTGTTTCCATGATAAAGATTAAAGCTAGATGCTGTTATGATTCCTGTTTTTTTAGGTTCCATGAAGTGTTAACAACTGTAATTCAGATTAAAGACGTATCTGATGTTTGACATTTGTTGGTGTTGATTCAGACAAGACATCAATATACGTGGTTATATTTAATTTCTAATATTTTCTAAATTACTACGAGTGCTTATGTGTTAATGTCTTGTTCGGTATTTGTGTGCACTTATAAAAGTTTTAACAAAACAAATCTCCTTGAAACATGATTCGAATCATGCCATTGAGCATGAAGTTAAAGCTTCCCATGATTCAAATCTCCAAGTGTATAATTTGAATCATGTGTGTACTATCATTAAATATAAGAAATCTGCAAAATGTGATTCAACCCACTTAGTTAAAGCTTGAACTCT

>Medtr4g021410 CTTACCTTACCTTGTGTCATGAAATATGTGCGCTAGAAATAAAATAAAAAAAGTTTTGAGATTGATATTGATGTGACCATCAACTTGCGTACTTCATTGTCTTTCTCTGTTCTCTGTCTATGGTCTGAGTTTGAGTTGTGTTCAATCATCACACCAGAAAAATGGGAAGTGAGGATCTGAGATTGTTTGAATTGAACACAGGAGCTAAGATTCCATCTGTTGGTTTAGGCACTTGGCTTGCTGCACCTGGTGTTGTCTATGATGCTATATCCACTGCTGTCAATGTTTCTTTCTTTCTTTTTAATTTTTCATTCCATTATTTTATTGCATCATGTTTTGTTTGTATTATTACATCATGTTTTGTTTTTATTATTACTATTATTGTAAATTTATGGTTTTGGAATTATTATTATTATGTTCATGCAGGTTGGTTATCGTCATATAGATTGTGCTCAAATTTATGGCAATGAAAAGGAGGTCAGTTTTGTTTTTTTCTTTTCTTTCAAATAAACATATATGATATGATGACATGTGTTTATGTTTATGATCATCATATTTATGTGTCACAAGAATAATTTTTAGATTAACATCGACCTCATGTTGTTAATGAGCTCCACCAAAGGACAAATTGTTCGGGATAACTCAAGTTCGATTCCTGGTGAAACAATTCTTGATCAAACTTTATTTACCTCCCGACCGAACTCCGGATTAGTGGGTCCTTTTCCCCAAGAACCGGAGGGTTAACACCAAAAGAAAAGAATAATTTTAGATTAATTAATTTTATTGTTTTCAGATTCAAGTAGGAGATAATCAGTAGTTGGACTCAAGTGGTTAATTGGTTTCAGTTAATTAAGGTAAGGTCAATTGGCAGCACCCGAGTTTGATCCCTGACTAGAACAATTGTGGGTCAAATATTACTTGAATTTAGAACTACTGATCTTTCTTATAGGAACCGGAGGATTAAAGAAAAATAATTCAAGTACAAGATGAAAATAATTGAATGTATTTGACAGAAAGGATTTAGAGGAAGACCTAGGAAAACTATTAGAGAAACCATTAGAAAGGATTTAGAGGTCAATGAGTTGGATCCAAATTTGATGTATGATAGAACACTATGACGTCATTTGATCCATGTAGCCGATCCCACTTAGTGGGATAAGGCTTGGTTGTTGTTGTTGTTGTATTTGACAGAACTTCCCCTGCATCTTGTGCTAGACAAGAGAAGCCAAGAACACTTTTTAGTTTGTTTTTGTCTCTTTGATTTTAATGATGAAAATTTAAAGCTTTTGCTGAATTTATGGTTGGCAGCTAGTCAGTTATCTTACCTTTTTAGTCTGAATTGAATCCGGAAATCGGAGTTTAAAACACTTATGCTAGACCACGGTGGTTAGTTATCTTACTTTGATAGTGAACAATGATTAAAAATATGAAATACAGTTTAATAGTTTATGATTAATTTTTTAAGAAACTTAGGAATATAAATAATTTGAACCTCTTGTATACAGAACAAAGAAAATATATTTTGTTTATTACAACAACAACAACCAATGTTATCCCACTAAGTAGGGTCGGCTACATGAATCAAACGACGCCATAGTGTTCTATCCAAAACCATATTTTTATCCAAATACAGTCCTTAGTTGAAGAAGATAGAATGTGTCTTGACATACTGTTTCTGCTTCGATGATGATGATCATTGGTGATGTTGGGATATCTGCAGACTGCAAAAACACTCTAATGCCTTAAGTTAGCAAGAAGGTATGAAGGTGCAAAGAGAATATGATTAGAACCTTAATAGGCTTGGTTGACTAGTAACAATACCATAAACCAAATTGCATATTTGCATATGTATAAAAGAAGCAAAATCAGTATAAACATCTCCATAACCACTTATAACTTATAAGGACTTTTTTTGGCAGTTAAGGATCAAATACCATTGAGAAGGTCCAATAAGTCAAAATCTAATCCGAACCGGGGTTTAATTCATCTGCCAAAGTTCATTCAAGAGAGTAGTTCTGTTACATTACACCATCATCTTTTTTCCCTTTTCTGGGGCTACTTGACGCTCTTTTTTTGTCTTTTTCTTTCTGAGCAAGCATTCGGTAGAATAAAGAGCTAACAATTAACAAATTTTATGCCATACTATAGTTGCAATTCATTCAGTATGTTGATTGTAGAATTTGAATTTATTTTGTTAGATATTTTATAATCATTTTGTTTGTACACTGTTGTGGTAATGTTTCTATCTGTTAACATTTTGTAAATGTTATGTAGATTGGTGATGCCTTGAAGAAGTTGTTTGCTAATGGAGTAGTTAAGCGTGAAGAGATGTGGATCACGTCCAAGCTATGGTTTGTCCTTTTATTACTAACAGAGTTGTTAAGCGTATATTATTGTTTAGATGTTTGAACAAATTTATAAAAAGAAATGACTTTTTCAACAGCAAAGTCTACAGACAACTGGGTTTTGTTTTAACTGCTGTTGAAAATTTGTTCTTGAACATGGTTAGATATAGTGTTAATTGGGTTTAGTTCGTATACATTGTCCGTGTAGACTGTTAATCAAAAGTAATCATCAAATCATAAAAAAGGTTTTACTTTAATAGTAACTACTTCTTAAGTCACACATATGATTGGTTGTGATTTGTTGATAGCGACACTGTATCAAAATTGAACTGTTATCAATTTTCTGCTCACGATTGCATTTTTAAGGTGTACTGATCACTTGCCAGAAGACGTCCCAAAGGCATTTGATAGAACTTTACGCGATCTGCAGCTGGACTATCTTGATCTCTACCTTGTATGTAAATGACAAATTAATTTCTTCCTACATCAGATCCTATTATGTTCGCAAGTTATAGTGTCACTGAAGTTGTGTTCTTAATTCTTATTATTAAAAATTCCTTTTTCATATGTCTGTATTCTACTAGATTCACTGGCCAGTGAGCATAAAAAATGGACATCTTACAAAACCTGACATACCAAGCACATGGAAAGCAATGGAGGCACTCTATGATTCCGGAAAGGCTCGAGCTATAGGAGTCAGCAATTTCTCTGTAAAGAAGCTTCAGGATCTGTTGGATGTGGGACGTGTGCCTCCAGCCGTTAACCAAGTGGAATTGCATCCTCAATTGCAGCAGCCAAATTTACATACTTTCTGTAAATCCAAGGGAGTGCACTTATCTGTGAGTATGCAAATTTTATGTCTTGTATGATAATTACTTTAGTTGCATATGTCTTAATATAAAGAACTTAAAAGGGATTATTCATATTATCATGGTTATCAGTATGATACATGCGAGCCATATCTCGGTTTGATATAAAATTGAACCCAATCAATATGAATCTCCAGTATGTATCTGTTAGAACTAATCACATGGAAAACATGCTCTTGTGATTAGTTGCAAACAGGATTGGAATTAGGAATCACATTAATTTGTATTTTAGTAAATTCATTTTTAATAGTCGTGTGTAGTTTGATTAGACGCACAAAAATTACAGATAATTACATGGTAAGGCTCCTCTGCATTTTTCTAGACTGCGTTCTTTTTAGTTCTTGCGCATATTTTGGTTTCCATCTGTGCCATAACCAGCTGACCCCCTTTTCCAGTGTGCGCCACCTCCAGATCTGCACATGAGAGTGTGTGCACCCACCTCCAGCTACCAGCACCAGCTGTTGCAAAATGCCCTCTTGCGAAACAATGCACGAGGAGAAGGGGGATGACTTAGAAATAATCAAACCCGAACTTGTTGTAACTATTGAAACATCTTTAGCCGCCCAATATCAAACCGAAGTTAATCAGCTTCATCGCAAAGTTGTGAGTGTTGCTTAGGTCAACGCCTTTGACACAGCCAACAAGTTTCCCTTTCGTCTGCATAGTATAATGAGTACCTCAAGGTCAAGGCACAAAATCGAATCACCACACAAGTTGCCAGCTGTCACTGTTGATTATATCGGTAATCCTGTTACCTTTGTTTCACAGTCCTCTTCAATTGGACCTGGACCCTTGACTCAGGCACCTCTGATCATATGTTTCGTAGCAAATGGTTTTTCTCCGATCGAACTTATATGACCACACTCCCTAGTGTTACTTTGGAAAAGGGATCACAATCCAAATGTGAAGGCATTGGCCAAACTATCCCTTTACACACACTTACTTTGGACTCTGTGCTTTACGTTCCCGATTGTCCATGCAATTTAATATCCGCACGTAAACTCACTCATGCCCTACCTTACTCTGTTACTAGGAAGACAATTAGAGCAGGGCATGAGTCTCGCAACTTGTATTACCTTTCGGCACCTACGCCGCCTATTGCTTGTTCAGCCATTGAATCTCCACCTGTTGTTCACCAACATTTGGGGCATCCTAGCCTTGAAAAGTCGCATCTTATGGTTCCGAGTCTCTCAAAAGTGTCTACCTTAAAGTGTGAGTCATATCAGTTCGGAAAACATACGAGGAATTCCTTTTGCGATAGAGTCAATAATCGTTTATTGACTCTAGTCGTCCTGTTTCATTCCAATGAATTACTATCTGAACTTAAGCCACTTTTTGTTGTCAACTGTGTAGAATTAATCACTTTTGTTTCATCATTTGATCTATGACTTGAATCTTAAACTATATTAAGGTTCGTCTATGATACATCAACTTATTATATATTTGTAATTGATTTTTACTTAAAAATATCTTATTGTTATTAATAATGTATTTTATTATTCATAATAAGTTTATATTCAAGTAAGGTCGAATAGGATCTTATTTAATAACAAATGGATATATGGCAGACATTTTATAGCTCCTTGAGTAAAACTTTAGAAAGTTTATATGATTATGAACATCAATTTTGATGACTACTTATCCCTATCAATGTTCTTATTCAGGCTTATTCACCACTAGGCAAAGGACTTGAGAGCAATATTCTTAAAAATCCGGTTCTGCACACGACTGCTGAGAAATTAGGGAAGACTCCGGCCCAAATAGCCCTTCGATGGGGACTACAAATGGGTCACAGTGTACTTCCAAAGAGCACAAATACAGCGAGGATTAAAGAAAACATTGATATATTTGACTGGTCTATACCAGAAGATTTGCTAGCTAACTTCAATGAATTTCAGCAGGCACGTCATTTCATGCAATCTTATTGTCACTTTATATGGTTGAATTCCAAAAAGATGTTAATTTATATTTCTTATAGCATTTTAATTGATACACAAGCATGACATTTTAAAATCAGTCTATGTGCAAAGGTCTATATACCAATTAAAAATGTTCTAGACTTAAATGACTTTTCTAGTATACAAAAAATTTGTCTTTGTTGATTGGTTCGTTGTTTGGTTTTAATCCCTCAGTAAATTTGATTTGATTAGATTCATGTTGAATTAGTCCCTCAATAAAATTGATGTCATGGTTTGGGGACTAAATCAATATAATTCAGATATTACAAGAACTAATTCAAAATGGTTCAAATATTGCAAGGACTATAATCAAAACAACGAAGATCAAAATAAAATATTCCTATTTTCATGGATTAGAACCAGCAATATTTCTTTTTAAGAGGACAAAAACCAAAATAGATGTATATTATAGGAACTAACAAATTATTTAAGTCATAGTTTCATTTCAACTTTCTCATTTACCGATGCAGGAAAGAGTAGTTCCAGGCGAACAATTTGTTAGTCAGACATCTCCTGGTTACAAAACAATTGCAGAACTCTGGGATGAAGAGTAAGAAACAAGTTCCATATGGGCTACTGCTTTTCCACTGTTGAATAATCTGACTTTTTTGTGACTGTTGTAATAATATATAATGTATTCATAATATGGGAAATAGGATGTGTTATTGAGGATTTGGTTTTGATATTCAATAAGAGAATTGTAATTTAGGGTCTACATGGAAACAACATATGACATGTTTATAAACTGTTTTCTGAAATGTCATCGAGTTTATGGTTTAAACATTTCCCTAA

>Medtr4g036845 GTAAACTCTTACACTACAAGAAAAAGAGAGGCTAGAAAGAAACACACTTGTTGAACATAAAAGTAAACTAAAAATGAGGTGCAATCATGTGCGTTTGAATTGTGGCATTACAATGCCTCTCATTGGATTTGGCACCTATTCCTACCCAAATGATAAGAAGACAACAGAAATTGCAGTCCACAATGCCCTTGAGGTACTAACCTTAATCACATACACATTTGTTGTATGAAGATCATATTGTTGTTATATATTGTTATATGAAGATCAAATAAACAGTAAACCATTAAATTGATCTCTGTCTTTGTAAGTCAGGAACTAATTTAAAATATTTTTTTGAAGAAGCTAAATTAGCTCATCCAAATTGTTACCAGAAAGAATCGAACACGAGACCTCGAAGAGGAACACACTCACATGTCCCAAGTCAATACCACCATGCCAACCCAAGTGGGTTAACTAAAGTCAGAGACTAATTTAAAAGTGACTTACATCAATTTAATGGTTTACTCATCAAATAACTTGTATATTGAAATATATTTTAAATCTGATATGAAATTAAAGTCCTCTCTTGTATTTTAAATTAATATAAGTAAAATAGGTATATGTGTTTAGTCTAAATTTGAACAAAAACATGTTCTTAATTAATTTATATTTAAGACTGCATGCTTTTCCTATCTATGATGAAAAATTTCTTGCTATTGTTCTTATTTTTCCTTATTATTGAGCACAAATATATGCATGTGTGTATGCAGGTGGGTTATAGACATTTTGATACAGCAAAAATTTATGGTTCTGAGCCAGCATTAGGCAATGCCATAAATAAGGCAATCTACAGGGGAGAAGTAGAAAGGGAAGATATTTTCTTGACATCCAAATTGTGGGGAAGTGATCACCATGATCCTGTTGCTGCATTGAAACAAACTCTAAAGTAAGTTAGAAAATAAACAAAATGCATATATCTTAATCCCTTTCATTTCTTGTAAAAAAAAATAAAAAATCTAAAATGGTATCAAAACTTCAGTTAAGATTTATTGGGCCACCTGTTATCAGGTTTCGGCTATCAGGTCAGCCACCATTTATGTCCACGAATCAAGCCCAATAATGCTGATCGTGAAGGGGTGTTAAAGAGTCTCGCATCAGATGGCATGAACATGTTTATAAGTGGAGGACAATCCTCACCTCACAAGTCAGTTTTGAGGGGTTGAGTTAGGTCCGACCACGAATTTTAAAAATAATTGAAAGTATATAACATGTGGTGACACAATAAGTAATGCCTATGATGTGACAGAATCTTTTTAATTACTTAAAAAAATCAGCTTTATTGTTGATTTAATATTGATTTTTTGTTTTTACTTGAATGCAAAAACAGGAATCTGGACATGGACTATTTGGATATGTACCTAGTGCATTGGCCTGTAAAGTTAAAACCATGGGTTAACTACCCTGTTCCTAATGAAGATGACTTTGAAAATCTTGATCTTGAGACAACATGGGTAGGGATGGAAAAATGTCTTGAAATGGGGTTGTGTAGGTCTATTGGAGTTAGTAATTTCTCTAGCAACAAGATTGAGTGGCTCTTAGATTTTGCTAATACACCTCCAGTTGTTAATCAGGTAATTAACACTAATTGGAATTCATCTTGAGTAGCACTTGTATCACATCACTATTACAAGAAAAAACCATTTTTGGGTACATTTAACAATTAATCTATTTGATGTATAATATAACTCACACATATTAATTATTGAATGAACTTAAAAAGTGATTTTTTATTTATAATAATAACTGAATGAAGTAACCCAATTGGGTTGATCTGATAGTGTTTGCTTAAGACTTTACGATGTGTTCATTTTAAGTTCGATTCTCCCTAATATCAATATCAATAGACTAATTTAACTTCTTAATAAAATGAGTAAATGAGGTAGTTTTATCATTGACAATACAAATAGAATAGAATAATTCAACAATTTTAATATCTTAATATATACTTAGGTGGAAATGCATCCTATGTGGAGGCAGAGAAAGCTAAGAAAGACATGTGGAGAGCACAAGATCCATGTAAGTGCCTACTCACCACTTGGTGGACCAGGAAATGCATGGGGATCTGCTGCTGTTGTTAATCATCCAATTATACAATCAATTGCCTTCAAACACCAAGCAACTCCAGCACAGGTAATTAAATTTCAAGTTCAAGACTTTTGTTTAGGAAAGGTTTTTGTAATCTAGAGTTCGACCAAACAAGTAAATAAAATATATTTAAATATTGTTTTAATCAAAATTCGAACTTGTCTATTTTTGACGGTGCATTACTTCCTACGTCTTAAATTGTATAATGTTTTGGGTATTTCACACATATTAAGAAATGTAATTAATATTGTGTGGAAAAGAGATATTATGAGTTGTTTTATAAAATTGTCCTTAATAAATGATATGAGAAAGATAAATGAAAGAATTGAAAGAAGAAAGAGTAATAAATAGTTAAGGATATAATAGGAAAAATAACATTAATGTTTCAATGGTATTATAAAGAGACATACAATTTGGAACAATTTTTTTATCTAAAGTGACATACAATTTGGGACGATAATAAATAAAAAGATTACTTTAATGTTCAAATAAATTTGACACATTATTTAAATGTACAATGATATCCAAATTAGTTGCATTTGTATCATTAGTAAAATGAGGGTTATGTATAAATAATTTAATAAGCTAATTTTTGACTATGATCTTTGAAGATTGCATTGAAATGGGGACTATCAAAGGGCTCAAGTGTGATTGTGAAAAGTTTCAATGAAGAAAGAATGGAGGAAAATATGGGATCATTTGATCTAAACTTGGATGATGAAGATATCTTTGAGATTGAAAAATTGGAAGAAATGAAGATTATGAGAGGGGAATTTCATGTTAATCAAACCACAAGTCCCTACAAAACAATTGAAGAACTTTGGGATGATGAAATTTGACGCCATAATCACTGTTATAATTCATAATTCATTTACTTCAAAAAAGGAAAAGTAAGATTATTAAAGAAAATTTTCTCAGCTCAAAACATTATGGTCTTTTCAATGACCATGATTCTTCTAATACTCAACTCTGAGAATCCGATAGCTGATTGTTTACAGTGATAAAAAGTCCCACTAAACCAACCTTTGTATTTTAATCATCA

>Medtr4g072060 TGCAGAGGTGAGAAGGAAAGAAAGAAAAAATGGCAATCACACTGAACAGCGGATTCAAGATGCCAATCATTGGACTTGGAGTTTGGCGCATGGAAGGACAAGCAATCAAAGACTTAATTATCAATTCCATAAAAATCGGTTATCGTCATTTTGATTGTGCTGGTATTTTCTTTTTTTACTCCTTCAAATTTAGATTTCAATCTAGTTTTTTAGCTCGTTGTTTTGAATTTTGATTGTGTTGTAGCTGACTACAAGAACGAAGCAGAAGTTGGAGAAGCACTTAAAGAAGCTTTTGACACTGGACTTGTGAAGAGGGAGGATCTTTTCATTACCACCAAGGTACAATTTGATTAGATACATGTGTGGAATAGGTTTACACCGTCAATGTATAAAAATTAAATCCTAATTCTAATTCTAATTATAAAGAAAGAAAGAAACAGCAACAAGGAAAACAAATATCGACGATAGCTATTTTAGAAAGTTGAGCTCCACGGGTGTTTTAAGCAGTTAAATTCATCCATAATTGATTTTATCCGAAAAAGATTAGCAATTTCTGCTTAGTTATTAAGAGTTAATGGTATTTGGTATTTGAATCTGATTTCAGCTTTGGAATTCTGATCATGGACATGTTGTTGAGGCTTGCAAAGATAGTCTTAAGAAGCTTCAGTTAGATTATCTGGATTTATATCTTGTTCACTTTCCTGTAGCCACTAGGCACACTGGTATGGTTGGTTTCTTATGCTTCCCTTGACTTTTTAGTTTTGATGATATGTAGAAACTATTTCTAAATGTATTGATCTATTAACTTGGACTCATATATATAGTTAAAATATATCTACTTTTAGCCAAATCTGCTCTATGCTTTTTAATAAAATATTTCTATACTTGGTGAAACTACGACTATCAAAATTTTGGTTTGGGATATTTCAAGGTTTAACCATAGCTATCAATAGGCATGAAGAGGCAGCCAAAAAAAAAAATATTATAATTGGGTAGGGGAGAGTAGGATGACATGACATGACATGCCGTCTTTTAAATATATATATATATATATATATATATATATATATATATGTGCTATTGGAAAATTATTCATCATTCATGAACTATTGATATCTAATAATAAAATGGCATGGCTCTTAAGCAAACCATAGAAGTAGTTAACAACAAAACTCAAGGAAACAAAAGAAGTTTAAGTCTAAAATTCCCGAAGCACCCGCATCTAAAACATCTCTTTTCAAAGGTGTTTTTATGGTCCCTCCCCCGGCGTGGCCGGTAGCACTGCTATTATTTGCACCTTAAATCATCAACACTTATATATTTGTTGAATTTTGCACTTTAGATCATCAACTCCTCCCAGCGATGGCCCGGCCCCCATATGAACATCTCTTTCTACAGCTAGAGAATAGTGACAGTCACTATTATTGGCCTCTATAGATAGCTATGAATTTAACAAGTGATCTAAATCACTCTTATATGTGTTCTATAATATAATTTTGCCACATTCCTCACACATGCAGGGGTTGGTACTACTGATAGTGCTTTGGGTGAAGATGGGGTGCTGGACATAGATACAACCATATCCCTGGAAACTACCTGGCATGCGATGGAAGGCCTTGTTTCATCGGGCTTGGTTCGCAGCATAGGAATCAGGTCTAATTTTGTGCTGCAAGCAATCTTCACTATGACTTCTAGAAGTTGCTCTGGAAATCCTTTTTACGACTCTAGTTTATGTGATGAATTTTACCTAATACTAAGGAAATAGTCAAATGAACAAAGATGAAAACAACTAATGACCAAACTTGATAGGTTCATGAGGACTGAAATTATTTCTCTAGTAATTATTCAGAGGCGCCTTAAATTGTGCCCTACAATTCCTGCATGCACATTTATGTTATTTTGGGATGATTACTTGCAACATATATTAAAAAACTTAACCAAGTATTTTGCAATATTCATCATGATATTACTAACGATCTTTCTCAGTAATTAAGAAGTGAATTGTATTGACATTTACAGATATATTCAAGACATTTTGTGTGCTGGATCCTTGTCACATGTTTCATATTACATTTAGCTATTGCCAGTTTACCGCCTACCCTAATGGAATAATAATGCTTAGTTGCTATTGATGGGCTGTTAGTGTAATCTATTGAAAAGGGGAATGTGTTGGATAACCTGTCTAAGTATAATTTGTGTGGTCTAATATTCATGTTATTGACTTCAGAAATCTTTGATTCTAATTAATGAAATTACAATCTGACAAAAAACTATTTGCAGCAACTATGACATCTTTCTGACTAGAGATTGCTTAGCATATTCCAAGATAAAGCCTGCTGTAAATCAGATTGAAACTCATCCATACTTCCAGCGTGAGTCTCTAGTCAAATTTTGTCAGAAGCATGGAATTTGTGTAACAGCCCACACTCCACTTGGAGGTGCTGCAGCAAACAAAGAATGGTTTGGTACAGAGTCATGTTTGGATGAGCAAATTCTCAAAGTGAGTGGTCATTATTAAGTTCCAACACATATTAGTAGCCTATTATCTTATCTTCCGACCATTATCTATCCAAAATCAGTAAATGTGACTAGTGTTGTGACTTTGATTTCAGGGTCTAGCTGAAAAATACAAAAAGACTGCTGCCCAGATTTCTCTTCGCTGGGGGATTCAAAGGAACACTGTTGTCATTCCTAAAACATCAAAACTGGAGAGATTGAAAGAGAACTTCCAGGTATTTGATTTTGAGTTGTCTAAAGAGGACATGGAGCTCATCAGCAGTATGGACAGGGAATATAGAACTAATCAACCGGCCAAGTTTTGGGGCATAGATCTTTATGCATGAGAATGCCCTTCCTTTCTAAACATGTATCATGGTCATCCAAATATGCTAGAAAACATCATGACCTTTCAACTTTGAGACTTGTATTGACATTTTGATTTGGCATATGCTAAACTTTTGGGCCATCCATTGTAGCTCATTTGAGTAGATATTATGAACTATGCTGGTAACGATAGTAAAATTATCTACCCGCTGTTATCAAATCTTGTGGACCAAATGAGGGAGAATCCAGAATATTTTGTTGCTTAATTTGCTTATCCATTTATTTACTCCCATCCTCGTCCATTTGGTCATTCATGTGAGCTTTTGGTTGATAAGTTTTCGAAATATTGAAAAG

>Medtr4g072320 AAATTATAAACTCAAATCTCAAAGTTAACATTTATAAGGGACAATTGTGAATTGTGTGAAGCTTGTGCCTTGAACCAACACAAAGAAGAAAAAAAAGATGGAAACAAAGAAAGTTCCAGAGGTGGTACTAAATTCAGGGAAAAAGATGCCAATGATAGGCTTTGGAACAGGAACAACTCCTCCTCAACAAATTATGCTTGATGCTATTGACATTGGCTACAGACATTTTGATACTGCTGCTCTTTATGGTACTGAAGAACCTCTAGGCCAAGCTGTGTCAAAGGCTTTAGAGCTAGGCCTCGTTAAAAATCGCGATGAACTCTTCATTACTTCCAAGTTATGGTGTACTGATGCTCAACATGACCTTGTTCTCCCAGCTCTCAAAACCACCCTCAAGCAAGCATAATTTTCTTAACCTTTTTTCTACTTCTTTCTAGTTTGTGCATTTTATTACTTGCCTAATATTATTGTTTTGGTTTTTTTGGATTGAAATTTTTAGAAATCTGGGATTGGAGTATGTTGATCTCTATTTGATTCACTGGCCAGTAAGGTTGAAACAAGATGCTGAAAGCTTAAAATTTAAAAAGGAGGATATGATTCCCTTTGATATAAAAGGAACATGGGAAGCTATGGAAGAATGTTATAGATTGGGCCTAGCAAAGTCTATTGGTGTCAGTAACTTTGGTGTCAAAAAGCTCTCCATACTCTTAGAAAATGCCGAAATCGCTCCTGCAGTTAATCAGGTAAAAGAATTTAGCACTAGTTGATTTGATTCATTTTGACATATAGATCAAATACACTTAATTGGTTGCCTAAGTCACTTCAAAATCGAAAAGCATCAGACATGTATAAACGTATCCGATACTGATACATGTCAGACACTTCCCTATATGAAAAGTATCCAATAATTTATTCTCATTCTTTCTAGAAATATTTCCACTGAAACTTCTCGAATACATTTTCATTACTTTCGTACCGACTAAGCACACAATTGTCATCTATATATGATACATCTTACTAATCATAGAGACACAGTGTTATATGTTTTTGTTACAAATTTTTGTTTGGATTTAGGTGGAGATGAACCCATCATGGCAACAGGGGAAACTAAGAGAATTCTGCAAGCAGAAAGGAATCCATGTGAGTGCATGGTCACCACTAGGAGGATACAAACTAAGTTGGGGTTCGCCTACAGTGATGGAGAATCCAATTCTGCACGAAATTGCAGAGGCTAGAAAGAAGAGTGTAGCCCAGGTGCAGCTCATTATTTTATTTCCTATGTTTAGCCAATTAAAATTTTAATAAGGTTTTTAAAATATACATCCCTTATTTGTTTTAAAATTCAGCTTCTTTTTTTAAATGGTCAAACTTTAGTCTTGGTCATAGAAGTCCTAATATAATATCACATCATGAACCAGCTCTTCAAAACCTTAGATTGTTAGGTGAGAATAAATAAATGGTTCTATATTTGATATCTTTTAACAGTTGAAACTTATCAAAGTCATATACATATACTACTACTACTACTATATTGAGATGACTGTTACACTGAAAGAAAGTCTATTTCTAATATTGACATATATTATGCAGATAGCACTAAGATGGATATACCAGCAAGGGGCAATTCCCATTGTGAAAAGCTTCAACAAGGAAAGGATGAAACAAAACATTGAAATATTTGATTGGGAATTGAACCAAGAAGAATTAGACAAAATCAGTCAGATTCATCAAAGCAGATTTCAAAAGGCAGAAATATTTGTATCTGATAATGGACCTTACAAAACCTTGGAAGAGCTGTGGGATGGTGATGTTTGAGCAAACTCTTGTTTGAATAAATGATGGGATCATGGAAAGTGTATGAGCTGCTGAATAGTTATAAGTAGTAAAATAATGCAAAAAGGGTTTTGCTCAAGAGATTAAAGACATCACAAATAAGATTTAAATTGTACTCAAATCCAAGTTCCTTATGTAAGTGACCTTAAATAAGAGAAAACACTAAGAAGGGTAATCTAAAAACATGGATCTAATGACTATTCCTGGACTAGAGAATCACATGGCATGCTATCTCAAATAACTGATACATTATACTTCCTTTCAATGTATAAATCATGCTAAGGGTTATCAATCCCTACAAAGCATGAAGTATGAAATCATTACTTACAATTTAATCTTTAAATTAATCACGCGTAGAAGTTTATTCTGTGTATAAATTCTTATTGATTAATTCTACTTTAT

>Medtr4g072350 GAAGATGGAAATAAACAAAGTTCCAGAGGTGATACTAAATTCAGGGAAAAATATGCCAATGATAGGTTTAGGGACATCAACAAGTCCCTCTCCACCACATGAAGTCCTCACCTCAATTTTGGTTGATGCCATTAAAATAGGCTACAGGCATTTTGATACTGCTTCTATCTATAATACTGAAGAACCTCTAGGCCAAGCTGTGTCAAAAGCTTTAGAGCTAGGCCTCGTTAAAAATCGCGATGAACTGTTCGTTACTTCCAAGTTATGGTGTACTGATGCTCACCATGACCTTGTTCTCCCAGCTCTCAAATCCACTCTCAAGCAAGCATAATTCTCTTAACCTTTTTTTCTTCTTCTTTTAAGTTTGTGTATTTTATTTGTCTAATATTGTTGTTTTGGTTTTTTGGATTGAAATTTTTTAGAAATCTGGGATTGGGGTATGTGGATCTCTATTTGATTCACTGGCCAGTAAGGTTGAAACAAGACGTTGAAGGCCATAACTTTAAAGGTGAGGATACAATTCCTTTTGACATAAAAGGAACATGGGAATCTATGGAAGATTGTTATAGATTGGGCATAGCAAAGTCTATTGGTGTCAGCAACTTTGGTATCAAAAAGCTCTCCATGCTCTTAGAAAATGCCGAAATTGCTCCTGCAGTTAACCAGGTATATGAATTTAGTACTACTTGATTTGATTCATTATGCCTCAGTTGCTTCAAAATGGAAGAGCATCATACACGTATCGTCAGATATTTCCCAAAACTTATAGTATCCAATAATTTATTGTAATTCTTTCGCAAAATATTTCCTTCGATACTTTTTTACCAACTAAGCACGCAATTGTCATCTAGATATGATATATCTTACTACTTATAGACACAACGAGCATCATACAAATTCATATTTGGATTTAGGTGGAAATGAACTCATCATGGCATCAGGGGAAACTTAGAGAATTCTGCAAGCAGAAAGGAATCCATGTGAGTGCATGGTCACCACTAGGAGGCTACAAACTAAGTTGGGGTTCGCCTGCAGTGATGGAGAATTTGATTCTGCGCAAAATTGCTGAGGCTAGAAAGAAGAGTGTAGCCCAGGTGCAGTTGATTATTTTATTTCAAATTATTTTTAATGATAACTATGACCTATGTTAATTCAATTTAGATTTTAATGAAGTTTTAAAATATGCAGCCCTGTTTTATAATTCAAGTTTTTTTTTTCAATGGTCAAACTTTAGACTTAGTCATAGAAGTGCTAATGCCACATCATGAACCTTAGATTGTTATGTGAAACAAATAAATAGTTTTATATTGAAACTTCATAAAGTCATACATATACCACTGTATTGAGATTACTGTTACATTGATAGTCTTTCTCTAATATTGACAAATATTATACAGATAGCACTAAGATGGATATACCAGCAAGGGGTAATTCCCATTGTGAAGAGCTTCAACAAGGAGAGGATGAAACAAAACATTGAAATATTTGATTGGGAATTGAACCAAGAAGAATTAGACAAAATTAATCAGATTCCTCAATGCAGACTGCTAAAGGCAGAAATGTTTGTATCTGATAATGGACCTTACAAGTCATTGGAAGAGCTGTGGGATGGCGACCCTTAAGCGAATTCTTGTTTAAATATTCGATGGGATCAATGCAAGTGTATGTACTGAA

>Medtr4g072360 ATGGAAGCAAAGAAAGTTCCAGAGGTGATACTAAATTCAGGGAAAAAGATGCCAATGATAGGTTTTGGAACATCAGAAAATCCCTCTCCACCACATGAAGTCCTCACCTCAATTTTGGTTGATGCCATTGAAATAGGCTACAGGCATTTTGATACTGCTTCTGTCTATAATACTGAAGAACCTCTAGGCCAAGCTGTGTCAAAAGCTTTAGAGCTAGGCCTCGTTAAAAATCGCGATGAACTGTTCGTTACTTCCAAGTTATGGTGTACTGATGCTCACCATGACCTTGTTCTCCCATCTCTCAAAACTACCATCAAGCAAGCATATTTCTCTTAACTTCTTTTTTTCCGCTTCTTTTACGTTTGTTATTTTTATTACTTGTCTACTTTTATTTTTGGGTTGAAATTGTACAGAAAACTGAAGTTGGACTATGTGGATCTCTATTTGATTCACTTTCCAGTGAGGTTGAAACAAGATGTTGAAGGCTATAACATTAAAAGTGAGGATATAATTCCTTTTGATATAAAAGGAACATGGGAAGCTATGGAATATTGTTATAGATTGGGCTTAGCAAAGTCTATTGGTGTTAGCAACTTTGGTATCAAAAAGCTCTCCATGCTCTTCGAAAGTGCCAAAATCTATCCTGCAGTTAATCAGGTATGACTTAAGAGAATTTAGTGCTAGTTGATTTGAGGAATTATGACGTAGAGATCAAATGCACTTATTGGTTGCGTCTGTTATTTCAAAATGGAAATGCATCATACACGTATCCGATACTCATTCACTTGTTAGTTGGATTTAGGTGGAAATGAACTCATCATGGCATCAGGGGAAACTCAGAGAATTCTGCAAGCAGAAAGGAATTCATGTGAGTGCATGGTCACCACTAGGAGGATACAAACTAAGTTTTGGTTCGCCTGCAGTGATGGAGAATTCAATTCTGCGAGAAATTGCTGAGGCTAGAAAGAAAAGTGTAGCCCAGGTGCAGCTCATTATTTTATTTCAAACTATTTTCAATGACACCTATGTTTAGCCAATTAAAATTTTAGTGAAGTTTTTAAAACTTACAGCCTGTTTTTCTAATTCTTTTTTTTTCTTTCTCAATGGTCAAACTTTAGACTTGGTCATAGAAGTGGCTAATACCACATCTTGAACCAACTCTTTATATTATATCAAAATGTCAAACTTCATAAAGTCGTACCTATACTACTGTTACATTGAAAGTCTATTTCTAATGTGGATATATATTATGCAGATAGCACTAAGATGGATATACCAGCAAGGGGCAATTCCCATTGTGAAAAGCTTCAACAAGGAAAGGATGAAACTAAACACTGAAATATTTGATTGGGAATTGAACCAAGAAGAATTCGACAAAATCAATCAGATTCCGCAATGCAGACTGCAAAAGGTAGAAATGTTTGTATCTGATAGTGGACCTTACAAAACCTTGGAAGAGCTGTGGGACGGTGACGCTTGAGCAAATTCTTGTTTATATATTCGATGTGATCATGGAAAGTTAATGTACTGAATAAATTATTGTTTAAATTCCTTATGCAATTGAAATTTAACTTTTATATAATATCTTTGATTCCCTAGCTGACATTAAATAAGAGAAAACACTAAGAAGAATACTTGGATTTATTTACTATTCCTCGACTAGAGAATCACATATTTGTAGTAAAGTATAAATTGTTTTTCATGAATTATGGAACAAGAGAAAAATTAAGCATATGCCAGAC

>Medtr4g092750 GTTACTTGCTGTTGGGGGTAAAATCATCATTTACTTGCGTTAATAGGAATGGATGACATGAATCATGATGATAGTTATCAAGAAGAAGCTCTGAAAAAGAGAGAGAGAGAGAGAGAGAGTCGAAAATGGAGTTACGTGAACTTGGAAGAACTGGACTTAAACTAAGCTCCGTCGGGTTCGGAGCTTCACCTCTGGGCAACGTCTTCGGTTCCGTTTCAGAAGAACAAGCAAACGCCTCTGTTCGTATCGCTTTTCAATCCGGCATCAACTTCTTCGACACTTCTCCGTACGCTATTTCCTCTCTCTCTTATTTCCTTTTCATTCTCTTCTTCAATTTTATTAACAGTTTTTCCAAATTTGAAGATACTACGGAGGAACACTGTCTGAGAAAGTGCTCGGAAAGGCGTTGAAAGCGTTGAACGTTCCGAGAAGTGAATACATTGTAGCAACTAAGTGCGGAAGGTACAAAGAGGGTTTTGATTTTAGTGCAGAGAGAGTGACCAGAAGTGTAGACGAGAGTTTGGAAAGGTTGCAACTTGATTACGTTGATATTCTTCAGTGTCATGATATTGAATTTGGATCGTTAGATCAGGTTTGTTACAAAAATAGCTTATACATAAGTGCTTAATTAAGTTGTTTATCCGAAGAGGATCTAATTTCAGTTGTAACTTAACTGGTAACTGTATTTCAAGGGGTGCTAACTGTTTGGCTTTGTTCAGATTGTTAACGAGACAATTCCTGCACTCCAGAAATTGAAGGAAGCAGGGAAGACTCGTTTCATTGGGATTACAGGACTTCCTTTGGAGGTATTTACTTATGTTCTTGATAGGGTTCCGCCTGGAACATTGGATGTGATACTTTCCTATTGCCATCACTCTATCAATGATTCGACTTTGGAGGATATAGTGCCCTATCTGAAGTCCAAGGGTGTTGGCATTATCAGTGCTTCTCCGTTGGCGATGGGTCTGCTCACTGAGGCTGGCCCTCCTGAATGGCATCCAGCTTCACCGGAACTTAAGGTCCTGTCTTATTAATTACGGATAAACTAATGAAAATTTGATAATACTGTATTTGTTAGATTGAGACCTGATTTTAGTTCATTTGGTAGCAAACATTAAATAAGAACCTTAATTTGCATGTTACAATCACACCTAAGTGTTGTAAGCTAGTTTTGCGAATGATTGCAAAAAACAAATCATGCTTGTTTGTGTATGTTTAAGTGAGTGAGAGAGAGTAAATAAGATCTTGCACCAAATCATCTTTTGCACACAAAAATAGTAATTTTAATTTGCTACTATATTTATTGGGCACTGCTATTTTTCTTATACAAAAGGATAACGTTAGTGGGATAGGGCTTGATTGTTTATGTTGTTGTTGTAAAAGGATAACATTTCCGGTGTATAAGAACTTAGTTCTGAGAGTATTTTATATATGTAAATCAGAAAGGAAATAAGTTCTGCCAGAGAAAGACAGAAAATTGAGCATGGAAATTTGAAATAGAATTCACATGTAGCACAGAAACTCAAATAACTGTATGTATAAGATCTGAGAGTAGTTTCATAGAAGACAGAAAACAATGAAAGAAAATATAAAATTTTAATTTGAGAGTGCACGCATATTAAGAAAAGAAGACTCAAATCAAATTAATTAGCCAAACTGCTGATTTCCCCCATATGATATATTGAACGAATGATCACTGCATTCAATATTTTTGGCAGATAAGAGAGTTGCCTCCTAGTACTCAATGATGTTTGTGGACATTTTGAACAATGTCTCTCATGAATCACTGTTTTCTGCTTATGGTCTGGTACATTGCATATTTCATCAAAACTAAACTAGTTCTATGTGTTTTGTTTGGAATATGCAGTCTGCTTGTAGAGCTGCTGCAACTTATTGCAAAGAAAATGGAAAAAACGTTTCAAAGTTAGCAATGCAGTACAGCTTGTTAAATAAAGAAATCACATCGGTGCTTGTTGGCATGAGATCTGTCGAACAGGTTCTGTTTCATCTTCATAATTTTTCATATTTAATTGCATACATACATCTATGTTCTGAAAATCTGTGATTTGGTGAATAGTGCATTAAGCCTTGCTCTAAAGCTTTGGTATCATTGTATAGCCTGGTCTATCAGATTGTTGCCTCTCTCTTTGTTGTTTTCATTAAAACATATATGAGCACTTTCCTCTGTAAAATATGTACTTGTTTAGCTTGGTCTATCAGATTGTTACATCTCTCTTTGTTGTTTTCATTAAAACATACATGAGCACTTTCCTCTGTAAAATATGTACTTGTTTAGCTTGGTCTATCAGATTGTTACCTCTCTCTTTGTTGTTTTCATTGAAACATATATGAGCAATTTCCTCTGTAAAATATGTACTTGTTTTTTCTTCTATCTTTCTCATACTAAACTAATCAAAGAGCAAGTCAAAAATTTAAAGAAATGTATGCCAATGCAATATTTAAGCAAGTGGGCTATCATCAGGAACCTAGACGAAGAACCTATCACATAATTTGTGGAGTTTTGTGCCGTAAACAAGTGGGCTATGATCTACTCCATAATTTAGTGACATGTATTGATCCGTTTGAAGGTCCTTTGTTAAGAGATTGACATTTGCATGCATTGGCTTTAAAATTTTCTATAATTTAGCTGTAAGATCGTTTTTAAGTTTTTGCAAGCAGCAGAAGTTTTTTAGATCCAAATGATTTGCAAGTAGTTCTATTGTTCATGCTGACATAGTTATATTACACTATAATAAGCAAACGTGGTTGTTTACTCTTGTCCTTCCTCATACTCTCTACCAACAGGAGTCTGAAAATGGAAAAATTCCTCGCATAAACATAGTGCACTCAGATCAATAATGTACTGTTGACACATTATTAATTATTGATAGACAGTCACAATTGATCAATGATTAACCACTATAATAATATATGTCAATAATGGAGTTCTTGGGTGCACTAGGCTTCTGCAAGAATTTTATACGAGTTTCTCATATCTCAAGTACAATTTCTCTCATATCTCAAGAACAATTTGTTAGACCAGTCACTTGATAAAGATGAACTGTTCTAAATTGAGAAAATACAGGTGGAGGAAAATGTTGCTGCCGCAAGAGAACTTGCAGCTTCTGGAATCGACGAAGCGGCTCTGTCAGAAGTCAGAACCATTCTAAAGCCTGTCAAAAATCAGTCATGGCCTAGCGGAATCCAGCAAAGTTGACCCCTTGCTGCCTGCATTGCCTATCAAGACTCAGTTTTCTTATTCCGGTTAAGAATGTTCATTATTCCTATTCAATAAGAGAAGTTACATCCACTATCCAAATTATTTGGTTTGTTGTTTATAGTACACGCGGTGTTTTTTGTGCTCATATCCTCCAGCTACTTGGGAGCAAGGTCAACTAGTATATATAGTCAATACAGCCCATCTTCTACTGCTGTTGTAACTTGGCATATAATGTGTTATAACATTTGTGTTATTTGAACATTAGATTTGAGATTTTGTAACGTCTGTGTTATTTGAACATAAGATTTGAGATACTGTATGGGACACTGTAGTTATCTCTATATCTTGCATTTTATGTTATACACCGGCCTCATATTTTATAAATACTCTGCAAAAACGACTTTGATATCTCG

>Medtr5g097910 CTTAGAAACACTATAGGTTCAAAATTGGATTACTCAAACAAAAACAAATAGCAATAATACAAGTGTCAATTTTTTCATAAGACTCTACATTTTTAGCTATAAAAAGGTTGCAAGCAAAGCCTACTCAATCATCATTCATCAAGCTATAGCATAATTCACTCAAAGTGTAACAATCTTAACATTGAACCTTAGTCCAACCCAAAAATAACAACATGGGCAGTGTTGAAATCCCAACAAAGGTTCTTACAAACACATCTAGTCAATTGAAGATGCCAGTGGTTGGAATGGGATCAGCCCCTGACTTCACATGTAAGAAAGACACAAAAGATGCAATCATTGAAGCCATCAAACAAGGTTATAGACACTTTGATACTGCTGCTGCATATGGCTCAGAACAAGCTCTTGGTGAAGCTTTGAAAGAAGCAATTGAACTTGGTCTTGTCACTAGACAAGACCTTTTTGTTACTTCTAAACTTTGGGTTACTGAAAATCATCCTCATCTTGTTATTCCTGCTCTTCAAAAATCTCTCAAGTAAGTAAATTTTAATTCATATCAATATTACATTCATTTGGAGGATGTTTGATTCTACAATGATAAAAATTAATTTTGATCTTGATTTGTTAATTTCATGTAGGACTCTTCAATTGGACTACTTGGATTTGTATTTGATTCATTGGCCACTTAGCTCTCAGCCTGGAAAGTTTACATTTCCAATTGATGTGGCAGATCTCTTACCATTTGATGTGAAGGGTGTTTGGGAATCCATGGAAGAAGGATTGAAACTTGGACTCACCAAAGCTATTGGAGTTAGTAACTTCTCTGTCAAGAAACTTGAAAATCTTCTCTCTGTTGCCACTATTCTTCCTGCAGTCAATCAAGTAAGTCATCAACTAAACCTAAGTTTTGATTAACTTATAGTAAACTTCTTAATTGTTAATATGTACTTATACAATGAAATCGTATATTTATAGGTGGAGATGAACCTTGCATGGCAACAAAAGAAGCTTAGAGAGTTTTGCAACGCAAACGGAATAGTGTTAACTGCATTTTCACCATTGAGGAAAGGTGCAAGCAGAGGACCAAATGAAGTTATGGAGAATGATATGCTTAAAGAGATTGCAGATGCACATGGAAAGTCTGTTGCACAAATTTCTCTAAGATGGTTATATGAACAAGGAGTCACTTTTGTTCCCAAGAGTTATGATAAGGAGAGAATGAACCAAAATTTGTGTATCTTTGATTGGTCATTGACAAAGGAGGATCATGAGAAGATTGATCAAATTAAGCAAAATCGTTTGATCCCTGGACCAACCAAACCAGGCATCAATGACCTCTATGATGATTAAAAAAAATGTTAAAGTCCTTTTAAATTTGCCATAAAATCTATCTTTTCGATTTACTTATTGTATACTTGTTGTTTCTTTTGAGCTTAGGATTACCCTTGTTTTTTAATCGAGTTTGAATAATCAATTTTACATTTCATTTTTCTCCAA

>Medtr6g073110 ATGGAAACAAAATCAATTCCAGAAGTTGTGCTTAATTCAGGCCACAAAATGCCAATGCTAGGGTTTGGCACTGGAACAGTACCTTTACCACCACATCATGAACTCATTCCAGCATTCATCAATGCAATAAAAGTTGGCTATAGACATTTTGATACTGCAGCTTATTATGGTTCAGAGGAATCTCTTGGTCAAGCCATAGCACAAGCATTAGAACAAGGTCTTATTAAAAATCGTAGTGACATTTTTGTCACTACTAAACTATGGTGTACTGAGTCACACCCTGGTCTTGTTTTCCCTGCACTGAAAAATTCATTGAAGTAAATCAATTACTCTCTTTCATTTCATTGGTGCAATTTTTAATATGAAATTTGAATAGTTTGGATAATTTTTTTACAGAAAGTTAATTTAGTTTTTATTGGAGCTTCTTTTCTCACATCCATGATTATTAAAAAACCGCACTTTTGGATTTATTCGTCCGGACAGGAAAAACACTGTTTTGTCCAAATTTACTCATCAGGTTGGAAGAGAAAACTTTGTTTTTCCTTTCCGTTTGGATGAATAAATCCAAAGGCTTGTGATTTTTTCTTGAATAGGGTAAAAATGGGAATAAATTCTCTTTGTTAAAACCATCTTTATTTTTCTTAATTCCTACATCTCAACCATTAAACACGAGAAAAAATTAAAAGAGGTATATTATAAAGAAAAGATGTGCTTGAGTTTGATGATTGAGATGTAACAATAAAAAGAAATGGAAAAGATTCATTGATTCTAACAACTGATTCCTGAGTGCATCATTCGGATGTTTCAATTCATACATGTGGATCTCACACCTATGAACGAGAGTTTTTTTATTTTTCTCTTAATATTTGGCATGTGTTGTATTTGATTATGTCTAGCTTGACTTAATTGTTTTATATATATATTTTTTTTGTGATGATTCATTCCATAAGGAGGTTGGGCCTAGAATATGTGGATCTATACCTAATCCATTTTCCAGTGAGGTTGAGACAAGGGGTAAAAGCGATCAACTACACCAATGAAGACATTCTTCCTTTTGATATGAAAGGGACATGGAAAGATATGGAACAATGTGCTAATCTGGGCTTGGCTAAGTCTATTGGTCTTAGCAATTTTGGTGTGAAAAAAATTTCAGAAATTCTAGAATATGCAACTATTCCTCCTGCTCTTGTCCAGGTACAATGCATTGATTTTTTTTCTTCCCTTTTTCATTTTCTTTTTCTTTTTCATTTTTGGGAAACCATTTAAAAATTTAGCTACTGATATAACCATTTAAAAATGTAGCTACTGATATACCTAATGTATTTGATATATATTATAGATCAAATACATTAGGTGTTGAATGAACCTAAAAAGTGATTTTTTGCTTATGGATATGACTGGAGGGAGTACAACGATTGGATTCGTTGTATTTTGAGATTAAGAAAAACATAATTAATTTTCAAAAATATCATCAATGTTCTGCCAATAACCCATTAGAAATATTCATTGGTTTCTTTGCCAGGTGGAAATGAATGCAGCATGGCATCAAGAATATCTGAGAAAGTTCTGCAAAGAGAAAGGGATTCATGTGAGTGCATGGTCCCCTTTGGGTGCTAATGGAGCACCGTGGGGTTCACTTGCTGTTATGGACAATCCAATAATAAAAGACATTGCAATCTCATCAGGAAAAGCTATAGCTCAGGTGAATTTTCATACTAATCAAATTTCTTTGCATTGTTAGGAATTTAGAGGTTCTTATAATAAATCATGGCACTAAAATTTATTTTTTGCATTATACAAGTTTTCAGTTATAGGTGTGATTGCTTTTACATAGTTATGTTACAACTTATGATATAGTAGAAAATAGTGAATAAATGTAACTAAAATCAAATATTATGATGAAGGAGAAATCTACCATTAATAATTTTCTTATTAAAAGCTGAGACTATTTATTTTGTCTCTTAATTAAATTGATTTTTTTTTTTTTAATCAAATAGTCTAGTGACTGAAGATCACACATTTTAAATGTGGAGAAATGGGTGTCCGGGTTCGAATCTCATCCCCTGCATATGTTATAAAAAAATAAAAAATACTCAAACACCCTTTATTTTTCTTTCGCCATAAAGGTAAATAAACACCCTAACACATTTTCAATTTCAGGTTGCATTAAGATGGCTAATAGAGCAAGGTGCTACTCCAATTGTGAAGAGCTTCAACAAGGAGAGAATGAAAAAAAATATTGAACTATTTGATTGGGAGCTAAGTGAGGTTGATTTGGAGAAGATTAAACAAGTATCACAATGCAGGGGTTTTAAAGGAGAACGTTTTATCACTGAAAATGGACCTTACAAAACTACTGAAGATCTTTTTGACTGA

>Medtr7g021670 ATGGCAACAGTAGGAAGAATGAAGTTAGGATCACAAGGGATGGAAGTATCCTTACAAGGACTTGGTTGCATGAGCATGTCAGCTTTCTATGGTCCTCCTAAGCCTGAATCTGATATGATTTCTCTCATCCACCATGCTATACAATCTGGTGTCACTTTTCTTGACACTTCTGATATTTATGGCCCTCACACCAATGAAGTTCTTCTTGGAAAGGTTACCACTTACTCATTCCCTTTTAGTTTTTCTTTTGATTAAAATTTCTGTGTATGCATTATTTTGATATTTCAATTTGTCTTTTCCTGCATGAAAATATATTTGATTGTTAGTTTTTGATGAAGTTGAAAATTGTGGAATAGGCTTTGAAGGTAGTTAGAGAGAAAGTTGAATTGGCTACAAAATTTGGAGTCAGAGCTGGTGATGGAAAAGTTGAGATCTGTGGTGATCCAGGCTATGTGAGAGTAGCTTGTGAGGGCAGCTTGAAGAGACTTGATATTGATTGTATTGATCTCTATTATCAACATCGTATTGAAAGCGAAGAGAAAGTGGTAACCTTTCCAAGAAGAACTTCATTGGTGTGAGGTCCATAGAAGTCAGAAGTATCAAGAAAAGTGACACCAGATTGTATGGCATGGTGAATGAGAGCAATCATGTCAGGTTCAGGTTTAGGAGGACCATAGAAAGCAGACATGCTCATGCAACCAAGTCCTTGTAAGGACACTTCCATGCCTTGTGATCCTAACTTCATTCTTCCTACTTTTGTCATATTCTGTCTTTTAAGCATGTTGGTAAAATATGTATTTGTGTGTCTTGTATCTTATTATCTATATAAATTAGGGAAGTTGCCATATTCTGTCTTTTAAGCATGTTGGTAAAATATGTTGCAGGATTTGCCTAGATTTCAACCTGAGAACCTGCAACAGAATCAGACCATTTTTGACAAAGTTAATGAACTGGCCACAAAGAAGGGATGTACTCCATCCCAGCTTGCATTGGCATGGCTTCATCACCAAGGAAACGACGTGTGCCCGATACCTGGAACCACCAAAATTGAGAACTTTAATCAAAACATCGGTGCTCTATCTGTGAAACTAACACAAGAAGAAATGGTAGAACTTGAGTCTTTAGCAGATTCTGTTAAGGGTGGTAGATATGTAGAGGATAAAAGTACATGGAAGTACTCTGATACTCCACCACTCTCTTTTTGTAAAGCTGCACAATGA

>Medtr7g021680 AAGATATCCTCTAAAAAAATAATTTAAGAAGGAAAGATATGCTAATTTGGTAAATAGAAGGTCAACTTATAAGCGTGTATTACAACATTATCTTTGTCTGTCTTTACATAGTATACATCACAGGACATTTGTTCTGTGTTACATCAGACTCAGAGCAATGGCAACAGTAGGAAGAATGAAGTTAGGATCACAAGGGATGGAAGTGTCCTTACAAGGACTTGGTTGCATGAGCATGTCTGCTTTCTATGGTCCTCCTAAACCTGAACCTGATATGATTTCTCTCATCCACCATGCTATACAATCTGGTGTCACTTTTCTTGACACTTCTGACATTTATGGCCCTCACACCAATGAAGTTCTTCTTGGAAAGGTTACCACTTTCTCTTACCCTTTTAGTTTTTCTTTTGATTAGAATTTCTGGGTTTGTATTATTTTTATATTTTGATTTGGTTTTTGTTGCACGATGTGATATGAGCATGAATGGAATTGTATATGACTGTGAATTTTTTATGAATAGGCTTTGAAGGGTGTGAGAGAGAAGGTTGAATTGGCTACTAAGTTTGGAGTCAGAGCTGGTGATGGAAAATTTGAGATCTGTGGTGATCCAGGTTATGTGAGAGAAGCTTGTGAGGGTAGCTTGAAGAGACTTGATATTGATTGTATTGATCTCTATTATCAACATCGTATTGATACTCGTCTTCCGATTGAAGTCACGGTAAAATTCTCTCTGATTTGATTTGGTAGCTTAAACTTTTGCATCAGCATATGTGATATTTTCCTAATTTATTATACCTTATGGTTTAGTAGCATATTTTCCTAATTTAAGATACGAAATAAACTGCTTGATGACAATATAAAATGACAATGTGTGGTGACTTCAACTTGGTTTTCTTTGTAAGATCGGAGAGCTTAAAAAACTTGTTGAAGAGGGAAAAATAAAATACATTGGTTTGTCTGAGGCCTCAGCTGCAACAATCAGAAGAGCACATGCAGTTCATCCAATAACAGCTGTGCAGTTGGAGTGGTCACTATGGTCAAGAGATGTCGAGGAAGACATAATTCCAACTTGCAGGTGGAATTTAGTTTGCAAACATTGTGTTTTGTTATCTAGTACTTATCTTTCTGGTATGAAGCACTTACATAGACACTGGACACGACACTGGCATGTGAAACCGTAACAATTTTAGAAGATAGAAGTAATTGAATGTAATTACATATGTTGGTGTCGTCTTAGTGTCGGAGACCAAACGCGCCTTTAATCGGTATTGTTTGTGCTACAAAGATATGAAGTCTAACTTCATGATTTATGATTTTCTTTCTTAGGGAACTGGGTATTGGAATAGTTGCATATAGTCCTCTTGGGCGAGGATTCTTTTCAACAGGAACAAAGTTACTCGACAACTTGCCACAGGATGATTACCGGAAGGTTTGTGGAAAGACTGCTTTTACAGAAATTCATGTGAATTACTTCTTCGTTTTGTTAATGATCATATTAGTCGTTTTGAGGAATTATGTATACTTATTTTCTGTAGCGATCTTCATTACACAATAATAGTGTTCACCGACTGATTCGCCATGATCAGAATTACCAAATTTGCTTATGTATATTCAGTTGACTGTAGTTCAAAATAAATTATAGTCGGTGTACATTTTTAGTCCATAAAATAGTTTGGGAATAGGCGAACAGAAGATGACACTGTAAGTTGATGCTAGTGAACACAATTCAGAACTTTATAATACAAACAGGGTCCATGGGATCAATGCGTGTTGATAACTCCTACCATTGACCCAAGAATTCCACTGTACTTACTATCCTTTCACTTCCCCTAATTAACCACTTAGTTCTAACATTGATATCTGAACCGCGAAGAGATATTTGATAACAAAAAAAATAAAAATTAAAAGAGAAAAAAAGTAGTGTGGTTCATAAATAAAAACGTTGGATCGTTTTATAGAAGGAAAATATGATGTATTTTTCATGAGTCCATGCATTTTGATCCTTTACTTCATGCATGATATATTTATCACATCTTCTTAATGGTGACTAAACACAAATATTATGGCATGAGAGAATTTTATATGATCAGACTGCACTATGTGTGAGTGTGCGTGCATGTGATTCCTTAACATACCATATTCTGTAGCTGCATTTTAGGATTTGTTACTACCTGACATCTTGTATCCTATGAAGTACGAACACAGACACTGGATACAACACTGATACTGAGAAAATGAATGCAGCTGAAAAATACAGACACACATAAATCAATCATGGTCACACATTTAGATCATAAATCCCTATAACTTACACCATTGATCAGGGAACTTATCTCATAATTGCCTCTTTGTTTGGTCTATAACAATATTAACTTTTCATACTGATATATTTCATAATCAATTATATTGAATGTAATCACATGAGTCAGAGAAATTGCAAATTCTCAAATTCTGTCTTTTAATGTCTATCAGTGATACCTTAATATAATCTTATATGTAAATTTGTTGCAGCATTTGCCTCGATTTCAAACTGAAAACCTGCAGCAGAATCAGACTATATTTGACAAAGTTAATGAATTGGCTACAAAGAAGGGATGTACTCCACCTCAGCTTGCACTAGCCTGGCTTCATCACCAAGGAAATGATGTGTGCCCAATACCTGGAACCACCAAAATTGAGAACTTGAATCAAAACATCGGTGCTCTATCTGTGAAACTAACACAAGAAGAAATGGTAGAACTTGAGTCTTTAGCAGATGCTGTTAAGGGTGGTAGATATGGGGATGAAATAAGTACATGGAAGAATTCTGATACTCCACCACTCTCTTCTTGGAAAGCTGTGTAATGAAGTGATTTTCTTGCTTAATGCTCATACTAGTCTTTTACCTTTAACAAATAAATCAGTATGAGTTTCCTTTTGAATGGATTTGGTTGTATAAAACTATGCTTTATGAATTGAACTAGACAGTTCAACCGGTTGAACTGTAAACCGGCACTGTCTATGGTTCATTGAACGGTTCGACTGTGGTTTTGCCAGTTCAATGTTTAAAACATTGGTATAAAAACTTAATAGTTTAAAGTATTTGATGCGGATTTTTAAGTTCGATTGAATTCTATCGATTACTCCACATAATTTGTTACATGTTTAAGGGCATGTAACTTAATTAACCGCCTGTTTGATAAACTATTGTATAGATGATTTCATCTATTACTTTTGGCCTCTAGTATTCAACTTCATCTTGAACTTTTGAGATATATTTCAATTATTTTTGTTAGGAAATATAATTTATAAGCTTAATTAATGTGATTATTCTTATTATTATTCTTTAAAGTGACAAAATTTCAATACACAGCTCATGTGTGAACAAGAAGATCGTAAGGTTGCTTAATTATACCAGATAATAATCTTTTCATCTGTATAAGGTCCTCAAGCCTTTCATTATTTGAGACAACCTGAATAGTTCATTATGATTATCAACATTTTGTGGAAAAGGCTTGGAAGCAAAATAAATGCATGCCTATTTTTGCCTTCAGCAAAGTCAGAAAACTACAATGATTTTAACCATGAAGTTTTGGGAAACACATTTAAGAAAGCTAACTATGGAGATTTGCATCAATGGCATTCAGAAATTTCTAAGTGTGGAAGGTTTCCCTTTGGGGTGCCAACCTAGTTGGGGTGTCAAGCTATCCCCTGATGCCTTCCTTTGCTTTTGATTC

>Medtr7g021850 CACAACTTCAATTTTGTTTGGCCTTTATACATAGTCCTAGGATAGAAGGTTTATTGAGTGTTGTGCAGTGAGACTAAGAACAATGGCAACAGTAGGAAGAATGAAGTTAGGATCACAAGGCATGGAAGTATCCTTACAAGGACTTGGTTGCATGAGCATGTCTGCTTTCTATGGCCCTCCTAAACCTCAAACTGACATGATTGCTCTCATCCACCATGCCATACAATCTGGTGTCACTTTTCTTGATACCTCTGACATATATGGCCCTCACACCAACGAACTCCTTCTTGGAAAGGTTACCACTTTCTCTTCGCTTTCATTTAGTTTTGCGATAACTACAATTGATTTTGACATGATCTATATGAAATGAACTTTTAATTGATGATGAGTTTTGAAATACAAATATACTACACAAAGTTATTGCCTAGCGTCGAGCTAAGTGACTTTGTAACCTTTGCGTAGCATAGTAGAAATGTGATGAAATGTGTATGTGTGAAATTGTGGACAGGCTTTGAAAGGAGGAGTTAGAGAGAAGGTTGAATTGGCTACAAAGTTTGGAGCCAAATATACTGAAGGGAAATTTGAGATTTGTGGTGATCCAGCTTATGTGAGAGAAGCTTGTGAAGCTAGCTTGAAGAGACTTGATATTGATTGCATCGATCTCTATTATCAACATCGTATTGATACTCGTCTTCCAATTGAAATCACGGTAAGATTCTCACAAAATTTCTTTGATTTGATTTTGGTAGCTTAAACTTTTGCATCAGGATATGTGATATATTCCTAATTTATTATACCTTTTGGTTTAGTTTGCATATTTTCCTAATTTAAGTTATGAAATGAACAACTTGATGACATATATAAAATGACAATGTGTGATGACTTCAACTTTTGGTTTTTTTCTAAGATTGGGGAGCTTAAGAAACTCGTTGAAGAGGGAAAAATAAAATACATTGGTTTGTCTGAAGCCTCAGCTTCAACAATCAGAAGAGCACATGCAGTTCATCCAATAACAGCTGTGCAATTGGAGTGGTCGCTATGGTCAAGAGATGTGGAAGAAGAAATAATTCCAACTTGCAGGTTTGATTTAGTTTACAAACATTATGTTATGTTATCTCGCATTTCTCTTTGTGGTACTTTATTAAAATTGCAATGATACGAAGGTATAAAACACGATAGCGATTATAATTTAAGAAAATGGAAGTGATTGAATGTACTTACATGTATTGGTGACATATGTCGATGCACCAACACGTGTCTGACACCAAACACACATCTTCAATCGTTGGTGCTGCATAGATACGAAGTCTAACCTCATGAGTTATGATTTTTTTGTTAGGGAACTTGGTATTGGAATTGTTGCATATAGTCCTCTTGGGAGAGGATTCTTTTCATCAGGAACAAAGATTGTTGAGAACTTTACAAAGGATGATTACCGCCAGGTTTGTCGAAAAGCATGCTTTTACAGAAATTCATATCAATTACTTGTTCATTATGTTCATCCACTCAACTAACAAGTATGAACTACCGGCACAAACATGGATACCTGACATGATATTGTCACTGACACATAGACATCGGTAATAATTTAAGAAAAACATAACTAGCTATTGTGTTGGTGTCAAACACAGAACACACCTTGAACTTGAAGGTTTCGCGTTATATAGATTCTAACCTTGGCACCCCAAAATGTTCTATTTTAATAAATATTCATGATTATTTTCTGCGGTGATCTCCACTTGCTATATTTTCACTATACCAGAACCGTGTTCACCTACTGATAGTGATAACAGTAAATTAGTGTCTTGAAATCCCAAGATCAGATCAAACTTTGTGTAGTGTGCATGGTTATAAAGTCATTGCTGGTGAACATAATCTATGTAGTTCAGATCAAAGGTGTGTCCAATGTCAAACGTGTCAATGTCTGACACGACTCTGATTTTATTACTTTCTAAAATTATTATCACTCACTTTGTGTTAGTATCAATGTCGTGTCCGTATATGTGTCATTGATTCATAGAGTTGTGTTACTTTTCCTTGCTGACACCTTGTAACATCAGGGAAATATCTTTATTATCTCATTGAATGTTCATCACTGTTGCCTAAATATAATCTTCTATTTGTGTATATGTGCTGCAGTATATGCCGAGATTTCAACCTGAAAACCTGCAGCAAAATCAGACTATATTTGAGAGGGTTAATGAACTGGCTGCTAAGAAGGGATGTACTCCATCTCAGCTTGCATTGGCATGGCTTCATCACCAAGGAAACGACGTGTGCCCGATACCTGGAACCACCAAAATAGAGAACTTTAATCAAAACATTGGTGCTTTATCTGTTAAACTAACACAAGAAGAAATGGCAGAAATTGAATCCTTGGCAGATCTTGTTGAGGGTGACAGAACCGGAAAGGAACCTACATGGAAGGAATCTGACACTCCACCACTTTCGTCTTGGAAAACTGCGTAATTAAGTGGTTTGAATGCTTAATGCTCATATCAACATAGTTTTAAATTGCGATTGTGGTTAGACTGCGTACTTTTATATTGTTGTAAAATAGTGACAAATGTGGCTGCAATTGCGGACCACACTTTAGAACCATGCATTATGCCATATGTATTTGCTACTACCTTAAGAAATAAATTGGCGTGAGAAAAATGATTTTTGCTGTGTAAACATCTCTAGTTTCTAGCATTTGTGTGTCTTTTTAAGCGGTTTGAACTCTATTGAAGACATACACTACAAGAAAAATGTTATCATGTAAAGCAACTTTATATTTTTCGACAAATTCCTTTAATGTAGTTTTCTTACATACATATTTGTCAAAAAAAGAATTAATGCTCTCTGATCTTTGAGTTGTTGACAAG

>Medtr7g063580 TCCGAAAAATTTGTTAAGAGAATCATGCAATGATCCACCTTTTATGCCGAACCTTTTGCTTTATTTCACATTTATATTTATCTTCAACAATTTTACTATGCAACATTTATAGCAAAATTATCTCAAGAAGGGTGTGTATTATTTGTTCAAAGTAAATTATTTTCATTCTTGTTTTTTATCTTTAGATTAATTATTGTTTTTTGTATCTTGATTTGAAATTTATGTAGGAGAAGATGGAAAAAGAAAATATACATGGTCCTCAACATTTTGATCTGAATACTGGTGCAAATATACCATCAGTTGGTCTTGGAACATGGAAAGCTTCTCCTGGTGTTGTTGGTGATGCTGTTGTTGCTGCAGTCAAGGTGCTTTCTCTGCTTTTTGTTTCTTAATTTCTTGGTTTCTTTATATGAAGCACATATACGGACACTGATACGCCGACACCGCTAATAATTTGAGAAAATCACATATTTCAGTGTAATTACATGTTTCAGTGTCAGACACGCACGTGTCCAACACAGGGACACGTTTAATCGGAGGAGTGTCTGTGACTCTGTGTTTCATACATTTCTTTTGAATTTGTTGTGTTAATCTATGAAACATTGACGCAGACACGCGGATGCTGTTAATAATTTAATAAACTAAAGTATTCAAATGTAACTACATGTGCTGTTGTATCGGTGCATTGACACATGTTGGACATCAGTCACGTCTTCAATCTGAAGTATCAGTGCTATGCTAGGTGCTAATTTGTGTTTTGAATGTATTGTCTCAATGCTTTGTTCAGGCTGGCTACAGACATATCGATTGTGCTCGGGTATATGATAATGAAAAAGAGGTAACCAAAATTATAAAGTTTATTTGAAATAAATAGTTTTGTATACTAATATTCTAGGTGGTCAAGAAAAAATTGTTTTTATTGTTCTGGTTTTCTTGATGCTAGGAAAAATGTATTTGCTGCAGATAGGCGAGGCGTTGAAGACACTGTTTTCTGCTGGGGTTGTACAGCGTGGTGAAATGTTCATCACATCAAAGCTATGGTATGTGTTTGGACTAATAATTCTTATTATGATAAAAGATAATTGACCTTTTTTTTTCTTCTTTCTGAATCAAGCTTTTGATGTATATGAGAGTGTGTGATATGTAGTATTTCATTTATTAGTTGATTATAAAATATGAGTATGTAGAAGAGGATGAAAAGTGGATCTGAAGATTAGGGTGTTGCCAGATTGAGCCTTCTGTAAACGAATTAGTTGCTAAAAACTATACAATGGTCACCAAATTCCTCCAGGAAAAGGAATAAATTGGTGATATAAAAGGACTAGTTTGTTGACAATTTTATGTATACTGGGACTAAGGGGTCATGGCTCAAATATTTAGAAGACACATATGATGACATTATAACATTGAGGGACTGATTTTGCTACAAAACTCCACATATGATGACATTATAACATTGTAATGAATTGTCTTCATATATGGCTTGCCTTTTTTTAATTTATCCATACAGGATTAGTGACTGTGCACCTGAAGATGTCTCGAAGGCACTGGCTAGGACTCTAGAAGACCTGCAGCTTGACTACATCGATTTATATCTGGTACGGTTCTTGATTTTAAACCCGTTTTCAATCCTGATGCAGAGACTGAAGATGGTTGTATGAAAAATTGATGTTTTCTTGGTAAATTATTTTGTCACAGATACACTGGCCGTTTAGGACGAAGTCGGGATCAAGGGGTTGGGACCCTGAGGTCATGGTTCCCTTATGTCTTTCAGAGACATGGAATGCAATGGAAGGTTTATTCGCCTCAGGTCAAGCACGTGCGATTGGTGTCAGCAACTTTTCAACTAAGAAGCTTCAAGACTTACTCGGATATGCTAAGATTCCGCCAGCAGTTAACCAAGTTGAATGCCATCCTGTTTGGCAACAACCAGCTCTTCATAATTTGTGCAATTCTACTGGTGTTCATCTCACGGTAATACCCTGACTAAGATTGATATAGTATAGTCCGAACGAGTTTTGGTCTTTAATAGATCAAATTTTAATAACAGTTGATATAGTATACAGAATTTAATCTGTATGCATCATAAGTGAAAAAATATTTATTACATTTATTGAGTATTGTCATTACAAAAACTCATTAAATGACATGACAACATACCATTGAATACATGTATAAATTGAATTCTATTCAGTGCTGCAAATAGCACTATAGGCATAGGAGGATTTAAACAAATTGCTATTGTTCTGTAATATGCTAGTTCTTACAAAGTGTTGTCAAATAATAGTTATAGCAGTACTATCTATAGCACTGTAGCATAGTGGCAACCCTTTATTTTTCTTAATCTGCGATTGAGAATACTGATTCTGTTGTAATATTTTATATGAATTTTGATAGAGTTTGTCTATCATAACCAAAACTTATCTAGACCATACTATACATGTCCATCTGAGTAATTTAAATGATATTTTTTGGTCCTGCATTTATGTATTTGAGGTTCTTTTTTGCTTACATTTTGTTAACAACAAATGCTTCCTTATAGGCATATTCTCCTCTCGGTTCTCCAGGATCATGGGTTAAGGGAGAAATCTTGAAGGAACCAATTTTGATTGAAATTGCTGAAAAACTTAACAAGTCTCCGGCACAAGTGGCTCTAAGGTGGGGTATCCAAAGTGGTCACAGTGTCCTTCCAAAGAGTGTAAATGAATCTAGGATCAAAGAGAATCTTAGCTTATTTGATTGGTGTATCCCTCCAGAACTCTTCTCAAAATTCTCACAGATTCACCAGGTTTGATCATTGCCTTTGTTGAAACTTGAAAACATAAAAACTAATCATTCTTTCCCACATCTATGCATAATTTTTTTATTCTTCTTCAGTTTAATCATTTATTTTGTTGTATAATGCAGCAAAGGCTACTTAGAGGGGACTTTGCAGTCCATGAAAGTTGTAGTCCATACAAAAGTCTTGAAGATTTGTGGGATGGAGAAATATGAAATGGAGATAATTTGCATTGCTATTTTGAC

>Medtr7g070500 ATGGCACAAACAGTTAAGCCACATGAACCAAAGACAAAGTCATTTGATCTGTTGAGTGGACATAGCATTCCTGCTATTGGATTAGGCACATGGAAATCTGGTTCACAAGCTATCAATTCTGTCTTCACAGCCATTACTGAGGTAACTTATGTAACATATTGAGATTGAGATCAGTTTTTGAGAATCATTATATTATTCATGATGAATATTAATAGTTTTTGTAAAATGAAAATTTTGAATTTTGTCTTTAGGCTGGATATAGACATATTGACACTGCTGCTCAGTATGGAGTTCAAGAAGAGGTATATATATCATATGCTTTCTGTTTCATATTAAGTTTTAATTTGTTGATTCTGGATATAAATTAAAAAATAAATAAATCTGAATTAATACGTTACTACAAGATTATAGTATATTAAAGTAAAGTGATTAGTTTCATATAAAGAGATAGATGATGAACCAACAATAGAATGATCCAAGTGGTTTAATACGTTAATACAAGATTATCGAACTTCTGACCACCACATGCTAAGAGCCCTAGGTCTAAGTGGTAACGGACTTGGCCCCTTAGCATGCAATGGGCAGGAGTTCGATTGTTGGCTCATGTGTACCGAAAAAATTCGGTTGAGAGTAGAGAATCAACCTTGTGTGTCCTACATATTCTCCGTTAACAACATTGTGTGTCCCACATATTCTCCAATGGAGATTAGTCGCTGTCACTTAGCAATAGAAACTTCTCTGTGTCAATATTATGGTAAAATAAAAAAGAGATAGGTGAGGATTGTTTACCTAGAGTTTAAGAAGGATTGATGAGAGTGAACTACTCTCTAATTAATGCATACAAACAATTGATTAATTTATTTATATAACACTTCTTACTTGTCCTGCTAAATAGGTTGGACATGCACTTCAATCTGCCATGCAAGCAGGAGTGGAAAGGAAGGATCTATTCATCACCTCCAAGATATGGTAATAATGTAATTACATATAGAGACATGTTAATACATATAATCTTTAGGTCTTTATGTTGATTCAATTGAATTTGATTGTGTGAAATTTTATGGCTGCTATTCTAGGTGCACTGACTTGACCCCTGAAAGGGTAAGACCTGCCCTAAACAACACCCTTCAAGAACTCCAACTTGACTACCTTGATCTTTACTTGGTAAATAATTAACAACCTTTCTATTAATCTTAAAATCTATACCTTTCTTGTTTCTAATTCTTTCATTCATGATGAATGTGATGCAGGTTCACTGGCCATTTCTATTGAAAGATGGGGCAAGCAGGCCTCCTAAAGCAGGAGAAGTGTCGGAGTTCGACATGGAAGGAGTTTGGAGAGAAATGGAGAAGCTTGTCAAGGAAAATCTTGTTAGAGACATTGGAATATGCAACTTCACTCTTACTAAACTGGATAAGCTAGTCAATATTGCTCAAGTTATGCCTTCTGTATGCCAGGTACAATTATGTTTACTCTACGAAGATTCATTTAATGTCTCAAGTTCTATGCTCTTGCTTGGATAACCAACTTAATTAAACATTTATAGCATAAGCACTTATCATATAAGTACTTATTTATATTTATAACAACAGATAAAATAAAGTCACAAGTGCATATGAAAGTAGTTAGGTTCAAATAGATTCAAAGTTAAATTAATTTATGTATTAATTACAAAAAAGAACTACTCCAATGATTTGTGTATATGGTGTTTTGTGATAGATGGAGATGCATCCTGGGTGGAGAAATGATAAGATGCTCGAGGCTTGCAAGAAGAATGGCATCCATGTCACGGTAATTTACACGAAGGTTTTAAATAAAGGGCGTGATAACGGTTTTGTCGTGATCTTTGGTACCGCGGTACCACAGAGAATTGCAATCAAATGAAATTAATGTGGCTTCAATTGCTGCCGCGTTATGTTGCTCAGATCGCGTACACAAACCTTTGTTTAAAACCTTGATTACTACACTCCTAATTTGATTTTCTTCATTTGTTTATATGGTTTTGTGATTGAATTTATGATTAGAATTGTCAAAATTGCTGTTATCTAGGCCTATTCACCACTTGGATCACAAGATGGTGGGAGAGATCTCATCCATGATCAAACGGTTGATAGGATAGCCAAGAAGCTGAACAAGAGTCCAGGGCAAGTGTTGGTGAAGTGGGCCATGCAGAGAGGGACAAGTGTCATTCCCAAATCAACCAACCCAAATAGGATCAAAGAGAATGTGGTTGTCTTCAATTGGGAACTTCCAGATAATGACTTCAACAAACTTAGCAAAATACCAGATCAGGTAGCTCACAAGCATTGGTCGAAATAGTTCCGATTTGATAAATAGATCTTTAAAATTGTTAATTTCATCCAAAATGGTCCATCCGTTGACTTCTACTATTGGAGCTATGACGTGGAACATTAACCAAATATGCTACATTGCATGTTCATCCCACGTTGACGTCATCCCATTAGTGCCTATGAAATTCAGGTCTTCTTGGGGACAAATTTGTCAGTAATTATTGGTAAATCAGTCGGTAAGAAACCTAAACTTGATAGACACGTCAATTCGCAATTTGATGACATTTATGTGGGATCAATGTGGAAGGGCCATGTATTTAATAAACGTGTCACATCATACTAATTGGAACTCATTTTTCGGGCGAATGATCACTTTCATTGATTATTGAAACATGCAGAGGAGAGTCCTCGACGGTGAAGACCTCTTTGTGAACAAGAGTGAAGGGCCATTCAAGAGTGTAGAAGATATCTGGGACCATGAAGATTAGACAATAACACTTGCATCAAGTTACTGTGTTTATCTTTTATTTCTTCTTTCCTTTGTTTGCAAGGCACTTGGTAGCATTAGTTTTAATTTACTAGTAGTTTATAAAAATGAGTTAAGATAGCTTTT

>Medtr7g114970 TTACTCTATTAGTGCTACTTTTTTTTTGTTTTTGAGGTAAATGTTATTTTTTTTATAAAGTGGAAACACTTAAATGATAATAAATATAAGATAAAATTTTAGTGAATAAAATTTAATTTAATTTATAATACATAGGATATATTAAATTAATAAATTTAAAGTATTTTTTTAAAGAAAAATTAGTTTACAAAATTACAAATACTTAAATTAATGATATAATTTTTATTATGAATTAAGAATACCTGTTATGTCATTTTACATTTATCAGCTAGTTGAACCGCTATTTTTACCAAACATTTCAGTTAATTTATTAGCTAAAAGCTATCAGCTAGCTTATCAGCTATCCGCTATTTTTACCAAACATAGCCTTAAAAATAAGATTGATGATGACTTGAATACACAACACCTGGAATAAGGACAGGGACTTACATGCCTAGACAATCAAATAAGCCAGCATTAAAATTAAAAAAGTCAATTAAGATTATTTAATGAAATAAAAAAGGAAAATGGTGGCATTATGACACTGCCCCAAGAGTTAGGTAACGCTCACCTACCGGCTGCCCCTATGAATACATTTTTGAAGAGGAACATACAAACAACAACACAACAGAGAAAAAGAGATAGAGTGGAAAATTTTGGAGTGGAGCAGAGAAACAGAAAGTGAGAAACACAAAAAAAATAAAAATGGGAGATACTATTCATATTCCTCGAGTGAAGCTTGGAAGCCAAGGCCTAGAAGTAAGTAACTTTTGTCCTTCCTTGAATTTTCACACTTCCTTGTTCCTGAGAATCATTGATAATATTTATTGTTGCTTTCAGGTTTCTAAGCTTGGATATGGATGTATGGGCCTCACTGGAGTATACAACGCTGCTGTTCCAGAAGATGTTGCCATATCTTTGATCAAACATGCTTTCTCCAAAGGAATCACTTTCTTTGACACTGCTGATTTTTATGCTGCACATACCAATGAAGTTTTTGTCGGAAAGGTACTACTTTCATTCACATATTTATTTTATTTTATTATGTTTTAGCATAACAAAAATGATGTAACATAAACATTTTAACAATTCTCAGGCACTTAAGGACATACCACGAGATCAAATTCAGATTGCTACAAAGTTTGGGATTGTCAAAATGGAATCTGGTAACGTTGTAGTAAATGGTAGTCCTGAATATGTTCGATCATGTTGTGAGGGTAGTCTTCAACGTCTTGGGGTGGATTACATTGATCTCTATTATCAGCACCGTATTGACACCACTGTTCCCATTGAGGACACTGTAAGTAATAGAATCTATTCTTAAAATTTGTTAGGAAGTGGTAGGTAGGTACTCGTGTTTTTGATTTGGTGTTTTGAATGAATCACAGATGGGAGAGCTTAAGAAGTTGGTTGAAGAGGGAAAGATTAAGTACATAGGATTATCTGAGGCTAGTACTGATACAATCAGAAGGGCACATGCTGTTCATCCCATTACTGCTGTTCAAATGGAATGGTCTCTTTGGACTCGTGAAATTGAGCCAGATATCATTCCCCTTTGCAGGTACCTAACTGATTGATTTTCTTTTTCTGTCTAACTTGCGGATTATCCATGATTATGACTGCAAAAGTATGTTTGGATTTACTGTGAATTTGCGCTGCCTTAATTTTGGCAAAATCGAAATTCACTCTCAATCCAAATATGCACCAAGTCGAAAATTTCAATTTTTGTTATGACTGTTTCAGTTATGAGCCATCTTAAATTTTACATTAGGCCTAACTCATCTTTAAAATACCGGCTTATAAGGTGGTGAATTTCATTCTATATAAACTTATTTCAGACCTTATCCTCTAAAAGGTGGGACTTCAATTTTTCACAATACACTCTCTCACGCTCAACACTATTGGGCTTGGTGTGTGAATAATTTGGTGGATTACTTGAAAGAATAGCCACTAATACCATGTTAAGAAGCATGATTTTTTGCCCAAAAAAGAGAGAATAAATTGAGAGAAGGATCGGTTATTTGGAGTTGATAGAATGAAAATGGTTGACTTTAATTAATGATCCTCCTAACTAAGCTAACAAATCTTAACTAACTTGCAATTAATACTAAACAACTTGTAACCATTATATCCAAGTCCAACAAATTCTAACAGAGGTCATTAGCATTTCTTCATGAAAGAGTTAAGTCATTTCCTTTTTATGCAGAACTTAGAAAAACTTCGGTCTTATTTCACGATTTCATAACTTCGGTTTGATAACTCTCAAAATTCAAATAAATAACTTCAAGTAAAACAAACATTAAGGGTTGAGTGGTTACTTCCTACAACTTTCTTTGTGTTATGTCACCTTTGTAATCTGTTTATAATTTTCATCTTGATGTAATTGTTGAGACAATTGAAAATGACAATAGTTCCTTGAAACAATGGCTTTTACATTGTTAGTGGTCTTAGAAATGGCAACAAAACACACCTTCTGTTTCATGTGATAGTGCTTAAACAAAGAAAGTTGTTTGATTTCTAGAGAAGAGATATACCTTTGATCGAATAGTTTTACTTCCTTGATTTGGAACCTTTTGATTTGAGTATGAATACCAAGTTCTTTCTTACAATTCCTAACTTAATGATTTTTGATATCCTTTACAATTCTTTCTCGAAATGACTTCAGTTGTTTGGTGACAACTCTTACAGGGAACTTGGCATTGGAATAGTACCGTACAGTCCCCTTGGCCGTGGATTTTTTGGAGGCAAGGCTATTACAGAAAGTGTACCTGCAGACAGTTTTCTGGTATGGACTCGACCCGATTTGTTTCTGATGACAGTCACTGTTAGCGCATATCATTTATAAGTGTCTAAATATAAGCTATTTCTATAATAAGAGATAATTAAAAGTCCAACTGTTTTTATATAAACAATAAGTACTTTTCGTAAGCTATCCTAGAGTCCTAAACAGTCTCATATATGTTTATACCAGTAAATAAATCCGAATGAACAAATCCAAACAGGTCCTTGTTCATTGTTCACCAACATCTCTTTTTTCACCAGGCAATCCAACCAAGGTTACAAGGGGAAAACTTTGACAAGAACAAGATCTTTTATCATCGGATGGAAAAGTTGGCACAAGAGAAGCATGAATGTACATCTTCACAACTTGCTCTTGCATGGATTCTTCATCAAGGAGACGATGTAGTGCCCATCCCTGGTAAGTAACTTTCCACTTTGTGACCTCAACAAGTTAGGCATCTATGGAGCTTACTCCCGTGTATAATTTGAATGTTCAGGGAAGGATACTAATCAATCTGTTATGACAAATTCACTACATGAAATTATTTGAAATTTTTGAAACCTCAATTATGATTCCTTTTGTTGTTGCAAATATGACTTTGCAGACTGTCATGTGTTGTAGGAACAACTAAGATAAAAAATCTTGAAAGTAATATCAGTTCGTTTAAAGTGAAACTCAACAAAAATGATTTGAAGGAGATTGAAGATGCTGTGCCAATATCTGAGGTGTCAGGGGATCGGACAACTGGTGCTTTTGTTCAATGTTCTTGGAAGTTTGCTAATACTCCAACAAAATCATAGCTTGATGCCAGTTTTGTACCTCAGTGTTTGAGACTCAAACTAGTTTCTGGTCTCTTTGAATGTTGTTAAAAATTTAATCCCATATAATAAAACCGATTGTCATTGGTCGAATGCGGATAACTCAGCAAAATTTGTGTTTCATGCTTAGTTGTGTCCATGTTATGTGATTAAACCAATTTGCTTATAATTAATGTGAAATCATGTTCTACTAATATATTCCTTGTCTGGAGAGAGATGTGAAATTGTGATTATTTTTCTCTCTGCCTGTTGTTCAAATTTTTAAAATAAACTAGGGCTAGGTTAATTTATACATCCA

>Medtr7g114980 ACCCGCTGACATACCGAGTGACGATATCTCACAAATCGAAGAAAGCGTAATGAGTAGGATGCGCTCTTCGTATATATATATATAGTCCGTGCAATGCAATTAGGAGTTTGTAATTATTCATCAATTCAACCAAAATGGCCACAACACAAACTGAATTAATTCCTCCTCCTCAGGTCCCACTTGGAACCCAAGGCTTTCAGGTTTCAAAATTGGGTTTCGGGTGTATGGGACTCACTGGAGCTTACAATGATCCTCTTCCTGAACAAGATGGTATTTCCGTAATTAACTATGCTTTCAGTAAAGGCGTTACTTTTTTTGATACTGCTGATATCTATGGAGGTAGTGGTGCTAATGAAATTTTACTTGGAAAGGTAATTTAATCATGTTTCAATTCAATTCAAACTTTATTATGCTAATTTGATTTTGTTGACTAGGCATTAAAGCAATTGCCCAGAGAAAAGATCCAGTTGGCTACAAAATTTGGTATATCTAGAAGAGACGTTTCTCGTCTCGCTGATGTGACCATCAAGGGTTCACCTGAGTACGTGCGCTCGTGTTGTGAAGCTAGCTTGAAACGTCTCGATGTTGAATACATTGATCTCTATTATCAGCACAGAATTGATACTTCTGTGTCTATTGAGGATACAGTTAAGGATATTTCTTCCTAGTACTCGACTACGCGTTACGTATATATGCTTACATTATTTGTTGAGAGTCCCAAATTATAAATGGGAGACTCCCATACCTTGATAAGCTGGTTTTCTAGGGTTGAGTTATTGACAATTCAAATTTGAAGATTATTTGGCTTATTTCACTAGATGTGTTGTTAACAAGTTTGTTGTGTTATAGGTAGGTGAACTTAAGAAACTGGTGGAAGAGGGAAAAGTTAAGTATATTGGACTATCTGAAGCCAGCCCTGATACAATAAGGAGAGCGCATGCTGTTCATCCCATCACTGCTGTACAAATAGAGTGGTCTCTTTGGACTCGCGACATTGAGGAGGAGATAGTTCCTCTCTGCAGGTTAGTTTGAGTTCCTCTCCAAGTTCATTGGTTAAGTGTTACTTGAAAAGAGGGTTAAGGAATAGGAACCATTTGCTCCTGGTTGCTTGTTTGTGTTAAATGTGACTTCACTAATTTAAAGCTTTTGTTGATACAGAGAGCTTGGTATTGGAATTGTACCATATAGTCCTCTTGGTCGTGGTTTCTTTGGTGGCAAAGGGGTTACGGAAAATGTGCCTGCTGTTAGCTCCCTGGTATAGTAGCATTTCAGAACTTAAATTATATGTCTGTTTCCGTTGCTTTCCATGGAATTGAACTATTACTAAGACATGCTAATTTATGTAATTGATCAAATCTTAATCATGTATTAAATATTGGTTGGTTTGTTGTCTTATTTTGTAAACAAACAAATGCATGCTTTTTTAAACTAGAGTAATGATACAGGGAATATCATCCTTATCTGTTTTTTTCTCACATAAATGATGCAAAGTCTTTTATAAAATTAATGCCCTTTCCTTTTTTAAAAGTGTATGTTATTTTCTGATAAATATTTCGCTTATCAGACTTCTCATCCCCGCTTCCAAGCTGAGAACTTGAACAAGAACAAAAACATATATGATAAGATCGAAAGTCTTGGCAAAAAGCATGGGTGCACTCCTGCTCAGTTAGCATTAGCATGGGTACTCCAACAAGGAAAGGATGTTGTGCCTATTCCCGGTAAGTGAAAAATATGCATATTTTTTTCGAAAATAGTATTATTGCAAAAACCTTTGATGCTAGCTACTCTTAGCTGGTATCCTAACAGTATCAGAAATATGAGATTTCGTCTTATTTGTTAATCTGAAGCCTGAACAAATTAGTATGCTCTGTATACTTCTATCGTTGTGCACTGATCAATCAAGAGTAGAACTATGTACACTCTTGTCTTTACACCAAAAAACTATGTATACACTTGTTAAAAAAGAAAACTTAACTCTAAAGAAAACAAATAAATAAATGCACAGAAGAACATAAATGATTTATCACGTAATCTGGTCAATTGTTCCTACGTCTCCGACAGCAAAGTGACTGCTGTAATTTTTCGATTATGTAATACTTAAGATTATAGAGTATAATGCAATGTTTAACTAGTGCGGTTTAGATTACAACTTTACAAGTTTACCCCTTAACCATGCAGTCTTTGGAGGGTTCAATACACTTGAGCATCTCCTCGCTTATTTATTCAACAACTTTCGTTTGCTACATATTGGCATTATGTGGCCTTAAATTTCATATATTATGAGTGTTATCTGATGTATTTGGGGTTATTGAAGGTTCTTCTAAATTTTTAAGTGCTGATAATGCACTATACAAGTCATGCATTCTTTTTTAAGCGCTGATAATGCACTATACAAGTCATGCATCTAAGTGTTTATTTAAGTTTTTATTTTCAGTTTTTTTATTCTTGAAAATATTGGGGACTTCTTTTTTCTCAGGAACAACGAAGATTAAGAATCTGGATCAAAACCTTGGTGCCTTAGCAGTGAAACTATCAGAAGAGGACCTGAGAGAAATTTCTGCAGCGGTTCCTGTGGATGATATAGCAGGTAGTAGATACTACAATGGATTTGATCATATTTCCTGGAAGTTTGCTAACACACCTCCAAAAGTTTGAAGTGTCTCAACCTGAAGTATAAATCCCTTTTAGTTGGAGAATGCAGTCAGTCATGAATAAAGCGCAGCAATGATTTCTTTATTTGTATTTAACTCTTTTATGACCTGGCTGAACAAATCAAGACCGGCTGTTCACAGTTGTTCTGCTGTTTTGATTAGTAAATAAATAATAAGGGATTTCAATCATATTCTCATAGGATTCCATGTTGGATGAATGTGAATGAAATGTTTTTTTTTCTATTTTTTTTTTCAGGCAATGTGAATGAAATGTTAAAACAAACTAATAGAAACCAAAATGTAGAAAAAGAAATAAAAAACAATGAAATTCGTGTTCATGTTGTTTCGATAGCTTGAATAAAAAATTGCTTAGGAATGTATATAATTGCGAAAATGGGTTGACATTGACCCAAGTTGTGTGACTGCACTCAGATTGACCCAACCCCAACTTGAGTGATTTAAATGAAAATATTCTTATTTTTATTTTAATTTGCACCCTGCTTTTTAAAGTAGGCTACCC

>Medtr7g114990 AAGCAATTACTATTGAACAACCAAAATGGCCGCAACACAAACACAAACACAAACTGAACTAATTCCTCATGTCTCACTTGGAACCCAAGGCTTTCAGGTATCATCACTTCACTCACTTGTACACTTACACTTACATATACAAATGTCATTTATATTCTTCTCTCTATACTATACTATACTAATTTACTACTACTACCTATTAATATTATTGTTTTTATTTTAGGTTTCAAAGTTCGGTTTAGGGTGTATGGCCCTCAGTGGAGGATACAATGATCCTCTTCCTGAAGAAATTGGCATTTCCGTAATTAATCATGCATTCAGTAAAGGCATCACTTTTTTTGACACCGCTGATGTTTATGGACTCGATGGTGGGAATGAAATTTTGGTTGGAAAGGTATATACATATTAATAATAACTACAAGAATTGGTACTTTGCCAAGAGGTCCTGGGTTTAAATCTGGACAGACGAGTAAATAGTAATCTCACAACTAACTAACTAGTAACATTTATCATTAAAAAAAATGCCAAGAATTTCTTTTATGTTCTAAATTTAATACCGCAATTGAAACTTATGCCAATTTGAGCTTTTTTTTTTCTTTCTTTTCATTATTTGATTTTGTTGACTAGGCTTTAAAGCAACTGCCCAGAGAAAAGATCCAGGTGGCTACAAAATTTGGTATATCCAGAAGTGGCGGTGGTATGGGCATCAAAGGTTCACCGGAGTATGTGCGTTCAAGCTGCGAAGCTAGCTTGAAACGTCTCAATGTTGAATACATTGATCTCTATTATCAGCACAGAGTCGATACAACCGTGCCTATTGAGGATACAGTTAAGGATATTTCTTCCTATGTAAAACTAGTATTTGATTTGAGCATAGTACACGGCAACAAGTTACATATACATGTTGATATGGTTAGATTATTTGTTGAGAGTCTCAAATTATAAATGGGAGACACCCCTACCTTTATAAACTGGTCTTGTAGGGTTGAGTTATTGACAATTCAAATTTGAAGATTATTTGGCTTATTTCACTAGATGTGTTGTTAACAAGTTTGTTGTGCTATAGGTAGGTGAACTTAAGAAACTGGTGGAAGAGGGAAAAGTTAAATATATTGGACTATCTGAAGCCAGCCCCGATACAATAAGGAGAGCACATGCTGTTCATCCTATCACAGCTTTACAAATAGAGTGGTCTCTCTGGACTCGCGACATTGAGAACGAGATAGTTCCTCTCTGCAGGTTAGTTTGAGTTCCTCTCCAAGTTCACATGATAAGATTACAACACTCATGATTGTTTGTTCATTAAATAATAATTCGAACAACTTTCACTAATTACGAGTTATTCAGTGGAACCGATTGCTCTTTGCTGTATTGAATTGCATGTTTGTGTTAATGAGATTTTACTTATTTCAAGCTTTTGTTGATATAGAGAGCTTGGTATTGGAATTGTACCATATAGTCCACTTGGTAAAGGTTTTTTTGCTGGCAAAGGAGTTATCGAAGATGTGCCGAGCTTCATGGTATAGCATTTTAGTAGTTGAATTGAAAGTGTGTTTCTCTTGCTTGCCATAGAATTACTATTAAGACATGCCAATTATGCAATAGCATTGATCAAATCTTAATCATGAATTAAATATTGATTGCTTTGTTGTCTTATTTTGTAAACAAACAAATGCTTGCTTTTTTAAACTTGAGTAATGATACAGGGGATATCATCCTTATCTGTTTTTTCTCACATGAATGATGTTTAAAGTCTTCTATAAAATTAAGGACCTTTCCTTTTTAAAAAATTTATGTTGTTTTCTGATGATTATTTCACTTATCAGACTTCTTTTCCCCGCTTCCAAGCTGAGAACTTGGTCAAGAACAAGGTTATATATGATAGGATTGAAAGTCTTAGCAAGAGGCATGGATGCACTACTGCTCAATTAGCATTAGCATGGGTACTCCAACAAGGCAAGGATGTTGTGCCTATTCCTGGTGAGTGACAAAATATGCACAATTTTTCGAAAACAGTATTCTCATAAAAATCTTTGATGGTAGGCTCTTAACTGGTATCCTAACAGTATCAGAAATATGAGTATTCCTCTTATTTGTGAATCTGAACAATTAAGAGAAGAACCATGTATCCTAACAATTAAGAATGCACCATATACTTCTTCTATCGTTGTGTACTGATCAATTAAGAAAAGAACTATGTACACTCGTATTTACACCAAAAAATACACTTTTGTTAGATACGAAAACCTAACTATAATAAAAACAAATAAATGCACACAAGAACATAAACAATTTATCACATAGTTTGGTTAATTGGTCCTACGTCTCCGACAACAGAGTGATTGTTGTAGTTTTTCGATTATGCAATACTTCAGATTATAGAGTACAATGTAACGTTATAAATCGTACGGTTCAGATTACAAGTTTACCCCTTATTGGTGAATATGAGTCATATTCAACAACTTTCCTTGCATTTCATATATTAAGAGTGTCATCTGATGTACTCAGGTTACTAACCAAAGGTTCTTCTAAATTTTTAAGTGCTGATAATGTACTATAGAAGCCATGCATGTTATGCTTTCCACCTAGTTTTATTTTCAGCTTTTTTTTATTCTTGAAAATATTATGGACTTCTTTTGTTCTCAGGAACAACCAAGATTGAGAATCTGGATCAAAACCTTGGTGCATTAGCAGTGAAACTATCAGAAGAGGACATGCGGGAAATTTCTGCTGCAGTTCCTGAAGATGATATAGCAGGTAGTAGATACTACAATGGAATGGATAGTTTATCCTGGAAGTTTGCTAACACACCTCCAAAAGTTTCAACGGTCTCAACATGAAGTAAAATCTCTTTTAGTTGGAGAATGCACTCGATCATGAATAAGGCGCAAACACAGCGATCATTTCTATATTTGTAGTTGCCTCAATTATGACATGCTTGATGAGAAAATCAAGACTGAGCTTTTGCATTTGTTCTGCTGTTTTCATTCCATAAATAATAATAAGGGATTTGAATCAAATTCTCATAGGATTCCATGTTAGATGAATGTGAATGAAATGGTTTTTTTGAGGCAATGTGAGTGAAATGTAAAAATTCAAAGAATTAATAGAAATAAAAAATTAAAACAAACAAA

>Medtr7g115010 ATGGCAACAATGCAAACCGAGCTAATTCCTCATGTTACACTTGGAACCCAAGGCTTTCAGGTTTCAAAAATGGGATTTGGGTGTATGGGACTCGGTGGAGCCTACAGTGATCTTCTTCCTGAGCAAGATGGTATTTCCATAATTAAGTATGCATTTAGTAAAGGCATCACTTTATTTGATACTGCTGATGTTTATGGAGTCGATGGTGGTAATGAAATATTGGTTGGAAAGGTACATGCATACAAATAACTACGTACCAAGAATTTCTTTAATGTTCTAAATTTTATATTGCAAATGAAACTTCATGCTAATTTTAGTTTATAATTATTTGGTTTTGTTGACTAGGCTTTAAAGCAACTACCTAGAGAAAAGGTCCAGGTGGCTACAAAATTCGGTATAGCCAGAAGCGATGATTCTGGTATGGTAATCAAGGTTCACCGGATTATGTGCGTTCATGCTGCGAAGCTAGCTTGAAACGTCTCGATGTCGAATACATTGATCTGTATTATCAGCACAGAGTGGATACATCTGTGCCTATAGAGGATACGGTAAGGTTTTCTATTTTGAGCATATAGTGCATCACATATGTTATTTCTTATTGATATACATTATCTGACTTATTACACTAGATGCATTGTTAACAAGTTTCTTCTATTACAGGTAGGTGAACTTATGAAGTTGGTGGAAGAAGGAAAAGTGAAGTATATAGGGCTATCTGAAGCTAGCCCAGATACAATAAGGAGAGCGCACACCATTCATCCCATCACAGCTGTACAAATGGAGTGGTCTCTTTGGACTCGTGACATTGAGGATGAGATAGTTCCACTATGCCGGTTAGTTTGAGTTCCTGTCCAAGTTCTTTGATTAAGTTACTTGAAAAGAGGGTTAAAGAAATAGAACTGTTTGCTCTTTTGGATGTTTGTGTTAAATGTTGTACTACTTCACTTATTTAAAGCTTTTGTTGATACAGAGAGCTTGGTATTGGAATTGTAACATATAGTCCTCTTGGTCGTGGTTTCTTTGGTGGCAAAGGAGTTACGGAAAATGTTTCAGCAGTTAGCTCCCTGGTAAAGCATTTTAGCACTTAAATTATATGTGTAGGATATACTTAATTATGTAATAGCATTGATCAAATCTTAATCATGAATTAAATATTGGTTGCTCTGTTGGCGTATTTTGTAAAATATGTATATGTCCATTCCATTGCAAATATATACTTGTTTTGTTGAACAAGAGAAACGATGAGGGGGATAACATCCTTATCTGTTTTTCTCACATGAATGATGTTTAAATTCTTGTATAAAATTAAGGCTTTTATGCTGTTTTCTGATATGATCATTTCACTATTCAGGCTACTCATCCTCGCTTCCAAGCCGAGAACTTGGACAAGAACAAAAGTTTATATGATCGAATTGAAAGTCTTGCCAAGAAACATGAGTGCGCTTCTGCTCAGTTAGCATTAGCATGGATACTCCAACAAGGCCATGATGTTGTGCCTATTCCTGGTGAGTGACAAATCAAAATAGTATATCACAAGAAACTTAATACTACTAGCTATGTATCCTATCAGTATCAGAAATATGATTTTTTTCCCCTTATTTGTCAATCTGCATAATATATTTATATCTTTGTGCTCGTGCATTGCTCAAATAAGTGAAAAACTATATACACTTTTGTTAGAATGACAAACCTGACTCTAGAGAAAACAGATAAATAAACACATATAAGTAATTAAACATTTGACCACCGAGTTCAGTCAATTGTGCCTACGTCTTTTATAGTTGAGTGGCTGCTGTACATTGTGGTGTTTATATTTATCTATTTACCTATGTTTCGTTTTGGAAATGCATATCTATTTACTATTCATTATTTCACACGTTTTCTTTGAATGTCAGGAACAACTAAGATTAAGAATCTTGATCAAAACCTTGGTGCCTTAGCCGTGAAACTATCAGAAGAGGACATGAGAGAAATTTCTACAGTGTTTTCCATTGATGATATAGCTGGTGGTAGACACTATGATGGATTGGATCAATCATCCTGGACCTGGCAATCTGCTAACACACCTCCAAAAGTTTAA

>Medtr8g070095 ATGTCCAAGGCAATTCAATTCTTTGATCTCAACACCGGAGCCAAGATCCCTTCCGTTGGCTTGGGAACTTGGCAAGCCGAGGACGATCCTGGCCTCGTTGCTGAAGCCGTCGCTACTGCCATCAAGGTAAATTTTCATTATCATTCATCCTTCCACTTGAGTTTTGGAAATCTCTTTGTATATAATATAATGAATAATGATATTTTCTTTGATACATACAGGCCGGTTACCGTCACATTGATTGTGCTCAATTATATGGCAATCAGAAGGAGGTAGCTATTTCATTTTTTGTTTTCTTAATGTTAATGCATATAATATGTGAAGGAAATGATTCATTGATTTCTTCTCTTGTTTGCAGATTGGCTTGGTGTTGAAGAAGTTGTTCGATGAGGGCGTAGTGAAGCGCGAAGATTTGTGGATTACCTCTAAACTCTGGTTTGTATGAATGTTTTAGCTAATTTATGAATGTGTTAGTCTTGTTCACTGTTTAGTAGTATCTAATAAGATGCTGTGCACTGCAGGAATACTGATCATGCTCCAGAAGATGTACCACTAGCATTGGAAAAGACTTTGGCAGAATTCCAGCTTGATTATGTTGATTTGTATCTTGTATGTAACAATTCTATCACTCAATCTTTTCATCTATGAAACACGGATACATGTTGTGTCCGACACTGACTTAACACCGACATATAATTATACTGAATTACGTGATTTTCTCAAATTATTAGCAGTGTCGACTTGTCAATGTCCGTGTCGTGACCGTATTCGTGCTTCATATATTTTCGTATATTCTCCTACTGCTTGGTTCTGATCATTACTTTCTTTCAAATGCGAAATCTGTTGTACATTGCTTGGTTAATAGACTGCATGTGCGTTATTATATAATTTGGTCTTTATATTTATATTTGTGAAGTTTTTATATCATATTGTATATTCCCTCAAAAAGATATCATATTGTATATGTAATGTGCTTTTCTTACTCAATATATTGGGTCTTAAACATTTTAGTTTTTAGGTTTAATTGAATTAATTCTTAATTAGAGTCCTTCATTATGAAAAGGAAAGTGAGATTTTGGTCTACTATGAACCCTATTCTTTAATTACCAAAAGATTCACTGTCCAATTCAAAATTTCACCATGAATTCATCCTCATACGTTGCATTTTTGTTATTTCATAATAAAAGATTCATTGTCCAATTCACAATTTTACATAGGATTGATGACTATAAGAATAAAATATTAAGAATAGATGACGGTTTATTTTTTTTGAAAGGATGGTTTATTTTGTTCAAATTAGCAATTGTTAATGTTACAAATAAAATTACTGAAACTATAATTTTGGGTGAAATAGTACATAATCTTTAATTTTATCAGACGAGTTTTTTGACACTATTATTTTAGTCACGCCAAAAATTTAGTCATGGATCCGGCCTAGGGTTTATTTCATCTAATTTCAAAATTTGATTTTTTAACAGTTTTATTTCAGACATAGACATACTCAATTTAACTGATGGTTCTTCCTTTTCAGATCCACTGGCCAGTTGCGATAAAGAAAGGACCAATAGGCGCCGAATTCACGGCTAAGGCTGAAGATCTTTTGATACCTAACTTAGCCGGCACATGGAAGGCAATGGAAGCATTGTATGATTCTGGCAAGGCAAGGGCTATAGGTGTAAGCAATTTCTCTATCAAGAAGTTGGGAGATTTGCTTGAGGTTGCTCGTGTTCCACCTGCTGTCAATCAAGTGGAATGCCATCCTTCCTGGAGGCAGGACAAACTCCGCGATTTCTGCAATTCCAAAGGTGTTCACTTCTCTGTAAGTTTCCGACTCTTAAATGATCATTATTTTGTTTCCGCACATTACGTTTACTATGTTCATGAAACCTTTTCGCTTTCTTAAGTCATTATATTTTTGCATGATTTTCTGTAATGTTTAGGGTTATTCACCTTTGGGCTCCCCAACCTGGCTTCACACTGATGTCCTTAAGCATCCAATTCTAAATGAGGTTGCTGAGAAACTAGGCAAGACTCCTGCTCAGGTAGCTCTTCGTTGGGGATTACAAATGGGTCATAGTGTGCTTCCCAAGAGTGCAAATGAAAAAAGGATAAAAGAAAACTTTGATGTTTTTGATTGGTCTATACCTGAACACTTGTTTGCTAAATTTGCTGAGATTGAACAGGTAAGTAGTATTTGATGGTTGCCCTAAGTCTACACCTATTGCTAGTCATTTTATATGTATGAAGCTATATCTGATTAAAGGAGTTGAGGAATGAAAAATCTAGGGTTAAATCCTGGTGAAGGAGATTTTTTTATTTTTTTTTTGTGGTGTACGAGGTTCGAACTTCAGACCTTGCATATATTATATTATGCATTGTCCTTTCTTATTGAGCTAAGCTCGCAGGGACGAAGGAGAAAAATACTAACGTAACAACTAACATTTTCCATTAAAAAAAAAACCTATAGTGTTGAAGTTCTTCTTTATTTTCATGGAAATATTGTCTAGTTTTATGACAATGGCTTTTATTGTTACAGGCAAGATTACTGAGGGGTGACATATTTGTTCATGAAACCTATGGTGCCTACAGATCTGTTGAAGAACTTTGGGATGGTGAAATCTAAGCGGTCTTCTCATAGCTGATTTGGTTGGAAGATGGAAGAAGAGAAGACCTTTTGTCTTGGTGTTGTGTTCTTTTTTTAAATAATAATATT

>Medtr8g070115 TTCCATTCTCATTCTCATTCTCATTTTCGCCCCCATTGAAGCCGACAAACCATTTTTCTCTTTTTCACCTAACAATGTCAAACGAGATTCGATTCTTCAGTCTCAACACCGGAGCCAAGATCCCTTCCGTCGGCTTGGGTACTTGGCAGTCTGATCCTGGCCTCGTCGCTCAAGCCGTTGCCGCCGCGATCAAGGTTTTCCTCTTCATCGATTTTCACACGCACATTCTCTAAATCTCATTTTACACAATCATATTTGATTTTGATTTTGATCAATCAATTTGATATATTACAGGCTGGTTACCGTCACATTGATTGTGCTCAAGTCTATGGCAATGAGAAGGAGGTAATTCTTTCGTCTTTTGTTTTCGCAATGATAGATAGTACAATTTCATGTGTGAATGAAATGATGGATTCATTCTTTCTGTTGTAGATTGGCTCTATTTTGAAGAAATTGTTTGCCGAAGGCGTAGTGAAACGTGAAGATTTGTGGATTACCTCTAAACTCTGGTTTGTTTCATCAATTAGTTTTGTTTCCATGTTTTAGCTAATTTTCTGTAAAAAAAACAATGTTTTAACTAATTTAATTATTTAATAATGCTCGTAGAATAGGTTTTTCAACTCTATAATGAATTGAATACATTGAATTGTGTCATTTTTTTCCGAATTATTATTGGTGTTGACGTGTCTGTGTTTATGATTCATAATTTTGAATCCATTAGTCTTCTTTAATGGTTAGACTTAGAACCTAAGGGCTTAAGATGATGTGCACTGCAGGAATACTGATCATGCTCCAGAAGATGTGCCGCTAGCATTGGACAGGACTTTGACAGACTTACAGCTTGATTATGTTGATTTGTATCTTGTATGTAATAATATTTAACTCAATCTTTAGATATTCTTCAACTACTTTGTTAAAATTTTATGCATAATTATGATTTTACTGCCCTCAATTCTCTGATCATTAATTGAAAACGAAATCTGAAATATGAATATCCAAGAAACATCCTTTACATGTAGTCTTTTAGTTATGAAATTGTGAGGTTTATTGTAGGAAAAATCCTATTGTACACGGCTTGGATAATAACTTTATTGTAGAATGTATGATTGTTTGGAAAATGCTATGTGTTTATCTTTTTCAATTCTAAACTTTTCTTACATAGCAACATGAAAATACTCCCGTCTCATATTAAGTGTCACGTTTGTAGTATTTGCTAATCTCATATTATTTATCACTTTAGAATATCAATGAAACTTTAATTACTTGTTTCACTAACAAACCTTCTAATTAGAGTATCTCAATTAACCTAACTACTCCATCTATATGGGTACTTTACTGAATGAAGTTCATTTTTATGATAGAAACTAGTACACTACATGATTTTCTTATGAAATGTGTGTAATCTTAAACGACATTTAAGATGAGATGGAGGGAGTAGTTGTTAAGCTATGCGTCTTGCAGACTTGACGACCATAAGTGCTTTTAATTCTAAGGAATTTAATTTGCACTTCTGGCATATGCTGCATTGCTTGGGTGCTATTATTTTATTCATTTTTTTATGCTTCCTTCTGATGCATGTAAGATTTATTTCATCTCTGATTGCAAAATTTGGTGGTTTAATATATTATTTAGTACATTCGTAATTGTTTATTGATGATTCTTCTTTTCCAGATCCACTGGCCTGCTCCGATGAAGAAAGGATCAGTAGGCTTCAAAGCTGAAAATCTAGTGCAACCTAACTTAGCCAGCACATGGAAGGCAATGGAAGCACTCTATGACTCAGGCAAGGCACGGGCTATAGGTGTAAGCAATTTCTCTTCCAAGAAGTTGGGGGATTTGCTCGAGGTTGCCCGTGTTCCTCCTGCTGTCAATCAAGTAGAATGCCATCCTTCCTGGAGGCAGGACAAGCTGCGTGATTTCTGTAATTCCAAAGGTGTCCACCTCTCTGTAAGTTTTCAAGTCTTGAATGCTCATTATTTTGTTTCTGCACATTACATTGGCTATTTTCATGATTTTTTTTTTTAGAGGATCTTTTCCGTTTGTTAAAACTTGAAATGAACCATTATTATTATATTTTTGAAGGATTTTCTCTAATTTAAAATGTGGTACTTTTAATGTTTAGGGATATTCACCTTTGGGTTCCCCTGGAACAACCTGGCTTCAAAGTGATGTCATTAAGCATCCAGTTCTTAACATGATTGCTGGGAAACTAGGCAAGACTCCTGCTCAGGTATCTCTTCGCTGGGGATTGCAAATGGGTCATAGTGTACTTCCCAAGAGTACAAATGAAGCAAGGATAAATGAAAATTTTGATGTTTTTGATTGGGCTATACCTGAAGACTTGTTTGCTAAATTTTCTGAGATTCAACAGGTAAAAATGCTTGATGGATTCCCTTGGTCTTTCTGGTTTAAGAACAGAATATATTAACTATATTGATATTCAGCTCTAGTTGTGAAGCAGCATAGCTAAAAATATTTGTTAGTCATCTCCTTTCATGTTATCAGCTATATATAAGAATAAGACTTGATTTTGGATTCTTAATTTCCTATTAATATTGCTAACCAAAACCTATTTTCCCTCTAATGTTTTGGGGATGGAGAGTTGGAATTCGTTTTTAGTTGAAGTATGAATCTATAAGGCTGAAGTTCTTCTTTACTTCCATGGAAACATTGTCCAGTTTTATGACAGTGGCTTTTATTGTTTCAGGCAAGATTACTCCGGGGTGCCTCATTTGTTCATGAAACTTATGGTGGCTACAGATCTGTTGAAGAACTTTGGGATGGTGAAATCTAAGCAGTCTTCTCAGTAGCAGATTTCGTCAGTGGATGGAATAAGAGAAAGCCTTTGTCTTTGTGTTGTAGGTGTTTGTGTGTGTGTGTGTTGTTAGATTGTTGACTTCATGAAATTTTACAAGGTGCTTATTTTGAGACTTTCTGTATCTCTGTTATTACCCAATCCATAAGAATATATGAACTTATGGAAACTATCTACCACCACCTCAGCATCTAACTCAAAATTACGGTGTCCAAGCCGAATCCACCAATTCAATAAATACCTTGAAGAATAGCGAAAGCTTCCCCCTCCTTAGATGTAAAAGGGGAGCTGAAGGTCCCTGTTCTTAAATGAACAAAACACCATCATCATGTAAATACATGTCAATGCCC

>Medtr8g088160 ATGTTGGTTACGTGGCTAAACCTAATTCGTAGATATTTTAACCATAGCCTCTCATTCATTCATCTCTCTCAAAGAAGTATAGAGAAAGTTGAATTGATGATGTCAGGTGGAGGAGGAGTTCCTGTCTTCAACCTTGCTCCTAATCTCAATGTTTCAAGGCTGTGTTTGGGAACCATGACATTTGGGGAACAGAACACGTTGTCTCAGTCATTTCAGCTTCTCGATGAAGCCTACCATGCTGGAATCAACTTCTTTGACTCCGCAGAAATGTAGTAACTGATAATAACAATGCAGTAGCACTACCTTCCCTTTTTCTATTTATTTATCTTCTTTTTTGTGTTGTGTTATGCAGGTATCCAGTGCCTCAACGTGCTCAGACTTGGGGAATGAGTGAGGAGTATTTTGGCCATTGGATTAAACACCGGAATATCCCCAGGGACCGTCTCGTTATTGCAACTAAGGTTTTCTTTCCATCAAACTACTATCTCTTTTAACTTATCAATTCTTCTCATCATTTAACATTTTGGAACTAGTTAGTTAGTTAGTTTGTCTAAAGGGATTATGTTGAGTATAGCACATATTTTATTCATAAGTGGACATGTTACTCGAGTCGGACCATGGTATTTAAATACAATACAAGTATTTTGAGATAGGAAAATGGATTTTCAGGGATTAACCGAGAAATTGCATACTCAAACACGGGGACTGAATAAGTTCCGGTGCTTAAGAATAAATGTAGAAATGCTAGAGAGAATTTTCATTTTCATATAGAAAAGGATAAGTTTTGTGAAATATGAGTTACCTTACCTTCATTCATGAATCATGATGGTTTTCCAAGCTATATAGCTCAGGCAGTTACAAAGTTTGGTTTATTGGAAAATTCTAGTATCCTTTCACTCACAAAGGACACCTCGCCAAAACAAATAACAACCAAAAGTGAAATTATGCTTATAGAGCAGACTAAGAAGGGCTAAACCTTCAATTAGGCAAATAAAACTATAAAATAGAGCAAATTTATTACATCATAAATTCCTGTAACATTGTAATATGCAACCCTAAGTTTGAATAATAGAGAGGCACTGCAACCATTATACGGTCGGAGATGTATGCAAAATTGACCGAACTTCGTGATCAAATCCAGTGTGTTCTCTGTTTGTGTTTTTTCGGTATCTAATCTGTTTTCTTTTGAAAAGGGTCGATGTTCCAACAGATTATAAGTTACTCCTTGTCTTTGCCTTATTGAATTTATTACCTATATCAATTTTGTCCCTTAGTCATTACCGAAATTGTAAATCCCCACCACATTAGTTATTTTGGATTGTTCAATTTTGACTCTAAAACTGTCACAATAGAACTATGCTATTAGGGACTTTCAATTTAAGGGTTCGTTTTGAAATTTCATTAATTTCAGGGACCATACTGTTTGGCGCTTACAAAGTCACGGATTAAATTGGTGATTCGCTCAATATTGGTTAAAATATTCCTTGTGATGTAACACTTGTATTTGGGACTAATTGTTAGATTCGACTTGATATTTACATGGATATATATCACTACTACCTTCTAGCATATTGTTAATTTTATTTCGATGTGTGTTGCATTTTTCAGTAAAGTCTTGTTTAAACCAAATTAGTATTCTTGGAGCATTGATGTTAATCGCAATACTACTATGGACAGGTTGCTGGACCATCTGGGCAGATGACTTGGATTAGAGGTGGTCCTAAATCTTTGGATGCCACCAATATTAGTCAAGCTATAGATAATAGGTTACTTACTCCTCCTTCCCAATTCACGTTTACATTACATCTTCTATCTAGTAGAATTCAATACTCATAATAGCTTTGCTTATTCAATTTATTTATGCAGTTTGTTGCGGATGCAATTGGATTATATAGATCTTTATCAAATTCATTGGCCTGATCGGTTTGTACCATTTCTTATTTTCATATTTTTTATATTGCATGTTTGCCAGCTTTCTTATTTTTGTCCTCTTAATGATCTTGTAGGTATGTTCCAATGTTTGGAGAAACCGAATATGATCCAGTCCAACAATACTCTTCAATTAGTATAGATGAACAACTTGACGCTCTTAGCAGAGCAGTGAATGACGGGAAGGTACAGTACTGTTAAATAGTTTCAACCATCAACCTATAAATAATATAGTTATTTGATTCCTGAAACCAAGAGTATGAAAATATCTTTGCAGATCAGATACATTGGTCTTAGTAATGAAACACCGTATGGCTTGATGAAGTTTATTCAGGTTGCAGAAAAAAGTTCTTCCTACCCAAAGATAGTTTCTTTGCAGGTTTTTTCACCTACTGCCATTTCGACTCCTGCAATTCTCTTTATAGATGTTTGCTTCAGGCATTAATTTTCTTCTGTTTAGTGACAGAATTCATATAGCTTGCTATGTAGGACTTTTGATTCTGCAATGGCTGAGTGCTGTCATCAGGAAAGGTAACCAAGATTTTGTTATAAACTATCATCTTTGGTTCTAATGCATTATTAAAATCAATTTTGCTTCTAAGATCACATCTGATCTGACTAGAAATGAAAAATTGTAGCTTTTGTGGTAGGTTGAGAATTTCATATTAAGATCTATTATAAGCTTTCGTTTGAGATAACGATTTCCAAATACAAATCAATTTATATCACACTTAATTTTAACCAAAATCAACTTTGCAAAATCAAGATCATCTAACATCAAGTTTGAAAATGCACACCCAAACATACACCAAGTGGTTGACCGTTTGATCATTGTCGCCTCCATTCTTCTAAATATGAATTGACTTTCAGTATAAGATAGGGTGTACTGGTGCTTTGTCCATGAGGGGACATTTAGATACCAAATTTCTATGCTTTCAACAACTTAAGCTTTTGGCATAATTGGTTCACAACAATTAGTAAAATATATTACTTGGGACAGTAAGGAGGCTAGAAAAGTTCATTGGATCAAATGGGATAAGGTTGTGTATCTAGCAAGAGATAGATGAGAGCGGATAGAAATGGTTTGTGGTTCGATAAAAGAAAAGTATGGAGTGCTTGATGGCAGGGATATAGGTAGTCCAAAACGGGGTTCAATCTGGTGTATAGATACTTAGGTTAACTGGTTTGCTAGAATAAGGTAGTTGTGGGAGAGGTTTGGTAGGTTGTATGGTTTCGCTCTTAACAAACAGGTTACAGTTGCCAAAATGGTGAACGGTGTTGTTGAGGTTGGGGGGAAGGAGTGGCTTTGGAGGAGAAATATGTTTCAGTCGGAGAGGCTATTACTGATTTGGAGGAGAAATATGTTTCAGTCAAATTTATATTATTCTGCAGAGTGGTGAAATTCTGTTTTCTTTGGATGCATGAACAATGAAAGCATAGTGCATGAAAATTTCATTATGACTTATAAGTGAATTTGAATATGATACTCAAGAACAAAATAATTTTCCTGTGTTTTCAGTGAAAGCTTCATTCTTGGATAATTGATTGATTTATCTTTTTCAGTATTAGCCTGTTGGCCTACAGTCCTCTAGCAATGGGTATTCTTTCAGGCAAATATTTTTCCCATGGTAATGGTCCAGCAGATGCTCGTTTAAATCTTTTCAAAGGTTTGTTGACACACTGTAACATATGCTTTGCTTTTATGTGTTTTCACCCCTTCCAGCATAAAAGGGAGAAAATATTAAATATGTTCCTAGTAGATCTAAATTTATCTTAAATACGCCATCACAGGAAAGTATTCAGAAGGAGAATCCAGATACAACTTGTCCAATAAAGCTATACAAGCAGCTGCTAGGGTAAGTAGAATTTCCAAAACATTGTTGATGATAATATTTTGATTATAGCTTATTGGTATGTAA

>Medtr8g088170 ATGGGAATGCTATTGTTACACCAAGATTGGTTAGTTTTCCCATTTTTCTTCTTCTTCCTTGCAATTTACCTAATCGGTTACTCCATTATCTTCCGTAACAAAAACCCCAAAATCCGATCCGAATTCTCCAGCTGTTTAATCTCCCTCTTTCACGGCACACCCGCCGCAATCTTCGGCGCCATCTCCATCTTCTCCGACCCCAACAGCGGCTTCGCATCCCTCAACACCGCTTTCCAGAAAACCGTTCTTGATTACAGCATCGCTTACTTCGTAACCGATCTATTACACTACGTCGTTTTCTTCCCAAGCGACGTTCTCTTCATCGCTCATCATTTAGCCACGCTTTTCGTTATCGTCACGTGTCGTCACGTCGTTTCTCATGGCTCTTTCTCCGTCGTCGTTTTGCTTGTTCTCGCTGAAGTCACCAGCGCGTGTCAGAATACATGGACGCTCACCGGTGCTTGCCGGAAGGAAAATCGCTTCGCCGCTAGGGTTTACGATGTTCTGTCCCCGCCGTTTTACGTGGTGTATTCTATTGTGAGGGGCTTTGTGGGCCCATACTTTGTGTTTAAAATGGTGGTTTTCTATGCCAGTGGGCTTGCGTATGGGCTTGTTCCTACGTGGATTTGGGTTTCTTGGGCTGTCGTGGTTTTTTCAGCTATTGGTGTTAGTATTTTGTGGGTATACACTCGTTGGGTTGAACTCATTAGTGAAAGAAGAACTGGTGCAAAATTAGAGGAAAAAATTAGATAGGCTTTGTTTTTTATTTTGTATTTGTAACCATTTATAGTTAGCATTGATAATTTTTTCCCTGTACTGTTGTAACCATTGATAGTTAACATTGTTCATTGCTTCCACATAAGAATTGATATGGTTTTGTTGGAAAGTAAATGTCATGTGTTGCTGAGTTTTTAACACAATCTTTTTTGCATGTTTTTATTGTCATATTGATTGCAATTTGGCTTGATTATTAGTTGATTTAAAGAATCTGATTTTATTTGAATGTGGAAAATTGTTTACTTTTGACTAACAGAGAGCATTGTATCTGATGTTGAAATCTTGAAAATGTACAATAGTGGGGTAGCATTCACCTGCATATGAAATTTGAGTGAGAAATTTTGTGCACTCTTGATGGCAAATGTTTGGATGTGTCTTATTTTATACTGGCCTAAGAATATCTTCCATCTTTTTTATGTCTTGTTTATTGTATCATAGATGAAGATGTTTAAAGCAGAAACTGATAGAACCTTGTGTTCCAAGTTGTTATTTTAATGTGAATACCCCTATCACGTCTTTTCCATTTTGCAGGAATACCTTGATATTGCAAAAACATATGGTCTTCATCCTGTATCACTTGCTATAGGTTTGAATGCCTCTCCCTCTTTTGATTGAATTTGGATGGGTCTTTGTTGTGTGATTATTTTATAGCTCAGTTATCTGCAAAGTTTGCACTCCTATTTTTTGTTTAGGCCTTACGTAACAAGCTTGTAGATAATCAATTTTTAACTAATCTAGTAGTATCAGCGTTGCTAAAGGACTGTGTTATTATCGTAGGTCATTTGCTGTTACATGAGTTAATTGGAGCACTTTTAGCTTCTCTTTTTCATTTGCTATTAGCTCAGGATTTTACATTTGTGTCCACGAGCTGCTGAGTTCGTCCTTTTGATGCTGCTGGTTAATAGTTTATTTAGCTTTTGTATATCAGATTTCCTGTTTTATTATATCCTTATTTTCTTTGCTGCTGTTCTTTGTGTTTTGAGCAGCTTTTGTTTTGCAACACCCTCTTGTTGCTAGTGTTGTTTTTGGGGCTACCAAATCATGGCAGCTCCGGGAAGTTATAAATGCATGCAAGATCAAGCTTACATCTGAAGTTATTGAAGAAATTAACAAGGTTCATTCAAGGTTTCCAAATCCATGTCCTTGA
