# Supplementary figures and images for: Analysis of Aldo–Keto Reductase Gene Family and Their Responses to Salt, Drought, and Abscisic Acid Stresses in Medicago truncatula
Source: Int J Mol Sci. 2020 Jan 23;21(3):754. doi: 10.3390/ijms21030754 (PMC7037683; doi:10.3390/ijms21030754)

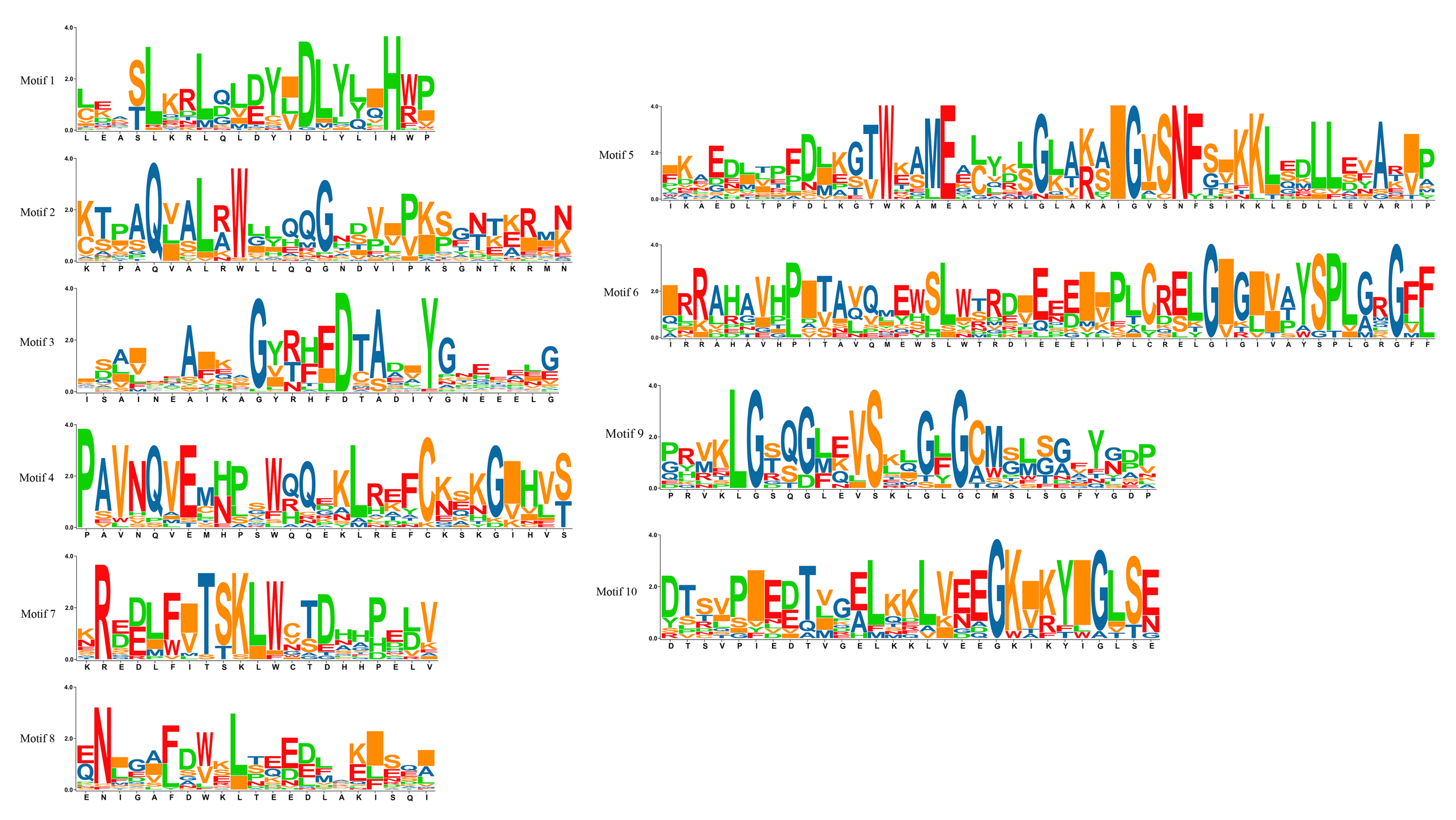

Supplement: Supplementary file 1 [file ijms-21-00754-s001.zip › Figure S1.tif]
